# Angelica sinensis polysaccharide as potential protectants against recurrent spontaneous abortion: focus on autophagy regulation

**DOI:** 10.3389/fmed.2025.1522503

**Published:** 2025-01-15

**Authors:** Yeli Sun, Guohua Li, Mengwen Kong, Junyuan Li, Shuyun Wang, Yuan Tan

**Affiliations:** ^1^Shanghai Key Laboratory of Maternal Fetal Medicine, Shanghai Institute of Maternal-Fetal Medicine and Gynecologic Oncology, Shanghai First Maternity and Infant Hospital, School of Medicine, Tongji University, Shanghai, China; ^2^Shanghai Key Laboratory of Maternal Fetal Medicine, Department of Reproductive Immunology, Shanghai Institute of Maternal-Fetal Medicine and Gynecologic Oncology, Shanghai First Maternity and Infant Hospital, School of Medicine, Tongji University, Shanghai, China; ^3^Department of Integrated Traditional Chinese Medicine (TCM) and Western Medicine, Shanghai First Maternity and Infant Hospital, School of Medicine, Tongji University, Shanghai, China

**Keywords:** recurrent spontaneous abortion, Angelica sinensis polysaccharide, autophagy, metabolomics, Beclin 1

## Abstract

**Introduction:**

Recurrent spontaneous abortion (RSA) represents a significant clinical challenge, with its underlying mechanisms yet to be fully elucidated. Despite advances in understanding, the precise pathophysiology driving RSA remains unclear. Angelica sinensis, a traditional herbal remedy, is frequently used as an adjunctive treatment for miscarriage. However, it remains uncertain whether its primary active component, Angelica sinensis polysaccharide (ASP), plays a definitive role in its therapeutic effects. The specific function and mechanism of ASP in the context of RSA require further investigation.

**Methods:**

In this study, we sought to evaluate autophagy levels at the maternal-fetal interface in RSA patients and in an RSA mouse model treated with ASP, complemented by a comprehensive metabolomic analysis. Autophagy flux in the decidua was compared between eight RSA patients and eight healthy pregnant women. Additionally, changes in autophagy flux were assessed in an RSA mouse model following ASP treatment, with embryos and placental tissues collected for subsequent metabolomic profiling.

**Results:**

Our results revealed a significant reduction in Beclin 1 protein levels in the decidua of RSA patients compared to the normal pregnancy group. Conversely, ASP treatment in the RSA mouse model restored autophagy-related protein expression, including ATG7, ATG16L, and Beclin 1, to levels higher than those observed in the untreated RSA group. Metabolomic analyses further identified significant changes in phosphatidylethanolamine levels between ASP-treated and control groups, with differential metabolites enriched in pathways related to glycolysis/gluconeogenesis, glycerolipid metabolism, and glycine, serine, and threonine metabolism. Functional assays revealed that ASP enhances trophoblast cell proliferation, migration, and invasion.

**Conclusion:**

In summary, our findings demonstrate diminished autophagy activity in RSA patients, while ASP appears to restore autophagy and regulate key metabolic pathways, including glycolysis/gluconeogenesis. These results provide new insights into the protective mechanisms of ASP in RSA, suggesting its potential as a therapeutic intervention for this condition.

## 1 Introduction

Recurrent spontaneous abortion (RSA) is a prevalent and clinically significant obstetric complication, defined by the occurrence of two or more consecutive pregnancy losses prior to the 28th week of gestation (1). The known causes of RSA are diverse, including infectious agents, chromosomal abnormalities, hormonal and metabolic disorders, antiphospholipid syndrome, and structural abnormalities of the uterus (2–6). However, approximately 50% of RSA cases are classified as idiopathic, with no clearly identifiable underlying cause (7). Emerging evidence suggests that autophagy levels in trophoblast cells of RSA patients are dysregulated (8–11). However, whether RSA is driven by autophagy deficiency or excessive autophagy activation remains poorly understood, necessitating further investigation.

Autophagy is a fundamental cellular process that mediates the transport of intracellular components to lysosomes for degradation and recycling, ensuring cellular homeostasis and adaptation to stress (12). This highly orchestrated pathway is regulated by a network of autophagy-related genes (ATGs) and their associated proteins. Beyond its role in cellular maintenance, autophagy plays a pivotal role in early embryonic development and implantation (11). Dysregulated autophagy has been implicated in various pregnancy complications, including preeclampsia and fetal growth restriction (13, 14), However, investigations into the role of autophagy in RSA remain scarce, and existing findings are often inconsistent, highlighting the need for further focused research.

Angelica sinensis, a cornerstone of traditional Chinese medicine, has been historically employed for the treatment of gynecological disorders (15). Its primary bioactive components include Angelica sinensis polysaccharide (ASP), along with sugars such as xylose, galactose, glucose, arabinose, rhamnose, fucose, and galacturonic acid, with ASP recognized as the most significant therapeutic constituent (16). A growing body of research has highlighted the diverse pharmacological properties of ASP, including hepatoprotective effects (17, 18), anti-cancer activity (19, 20), anti-aging benefits (21–23), antioxidant capacity (24), and immune modulation (25).

Emerging evidence further supports the role of Angelica sinensis extracts in modulating autophagy (26, 27) and restoring immune balance in abortion-prone models (28, 29). Specifically, ASP have been shown to significantly suppress the expression of autophagy-related proteins, including microtubule-associated protein 1 light chain 3 (LC3)II/LC3I, thereby mitigating excessive mitochondrial autophagy (30, 31). Despite these promising findings, the precise regulatory effects of ASP on autophagy, particularly within the context of RSA, and its underlying mechanisms remain inadequately understood.

Thus, this study aims to elucidate whether ASP contributes to the adjunctive treatment of RSA through modulation of autophagy pathways and to identify the associated signaling pathways. Our findings demonstrate a significant reduction in Beclin 1 expression at the maternal-fetal interface in RSA patients, indicating impaired autophagy activity. In contrast, ASP treatment in RSA mice led to a marked upregulation of autophagy-related proteins, including ATG7, ATG16L, and Beclin 1, accompanied by alterations in the glycolysis/gluconeogenesis metabolic pathway. Furthermore, ASP was shown to enhance the proliferation, migration, and invasion of HTR-8/SVneo trophoblast cells, highlighting its potential to support trophoblast function.

## 2 Materials and methods

### 2.1 RSA animal model

The CBA/J and DBA/2 mouse strains are well-established models for investigating immune-mediated RSA. In this study, female CBA/J mice were mated with male DBA/2 mice (CBA/J × DBA/2) to induce RSA-like phenotypes, as previously described (1). A total of 30 female CBA/J mice (20 ± 2 g), 10 male DBA/2 mice (22 ± 2 g), and 5 male BALB/c mice (20 ± 2 g), all aged 9 weeks, were obtained from Beijing Huafukang Biotechnology Co., Ltd. During the experimental procedures, female mice were paired with male mice at a 2:1 mating ratio. During the experimental procedures, female mice were paired with male mice in a 2:1 mating ratio. The detection of a vaginal plug was designated as embryonic day 1 of pregnancy. The pregnant females were randomly divided into three experimental groups: a normal control group, a RSA control group and an ASP intervention group. The ASP group received daily oral gavage of ASP at a dose of 400 mg/kg (32), while the control groups were administered an equivalent volume of saline. Treatments commenced on the first day of pregnancy and continued for 2 weeks. At the end of the treatment period, all mice were anesthetized and euthanized in accordance with humane protocols. All experimental procedures were conducted under the approval of the Ethics Committee of Shanghai First Maternity and Infant Hospital.

### 2.2 Sample collection

Embryos and placentas were carefully harvested from the experimental mice and immediately rinsed with ice-cold phosphate-buffered saline (PBS) to remove residual blood and debris. The cleaned samples were then subjected to rapid flash-freezing in liquid nitrogen to preserve molecular integrity and stored at −80°C until further analysis.

### 2.3 Sample preparation

Frozen embryo and placenta samples, each weighing 15 milligrams, were carefully transferred into 1.5 mL Eppendorf tubes. To optimize the extraction process, two small steel beads were added to each tube, along with 0.3 mL of a methanol-to-water solution (4:1, vol/vol). Additionally, a reference solution containing 0.3 mg of L-2-chlorophenylalanine dissolved in methanol was included in each tube. The samples were subsequently incubated at −20°C for 30 min to enhance the extraction efficiency.

Following the initial storage period, the samples were subjected to ultrasonic extraction in an ice-water bath for 10 min to ensure thorough processing. They were then briefly stored at −20°C for 2 min before being ground at 60 Hz for 2 min to achieve a uniform mixture. Subsequently, the samples were centrifuged at 4°C and 13,000 rpm for 10 min, facilitating the separation of the supernatant. The collected supernatant was concentrated and dried using a freeze-drying centrifuge, yielding a final volume of 250 μL.

Each dried sample was processed with 300 μL of a methanol–water mixture (1:4, vol/vol). The mixture was vortexed for 30 s and subjected to ultrasonic extraction in an ice-water bath for 3 min. The samples were then incubated at −20°C for 2 h to ensure thorough extraction. Following incubation, the samples were centrifuged at 13,000 rpm for 10 min at 4°C. A 150 μL aliquot of the supernatant was carefully collected with a crystal syringe, passed through a 0.22 μm microfilter, and transferred to LC vials. These vials were subsequently stored at −80°C to maintain sample integrity prior to liquid chromatography-mass spectrometry (LC–MS) analysis.

To ensure the reliability and consistency of the LC–MS analysis, a pooled sample derived from equal portions of each individual sample was prepared and utilized as a quality control (QC) sample.

### 2.4 LC–MS analysis

Metabolic profiling of the collected samples was conducted using a cutting-edge UPLC i-Series system (Waters Corporation, Milford, United States) coupled with the advanced VION IMS QTOF mass spectrometer (Waters Corporation, Milford, United States). Chromatographic separation was achieved on a high-resolution UPLC BEH C18 column (1.7 μm, 2.1 × 100 mm) in both positive and negative ionization modes. The mobile phase comprised water with 0.1% formic acid as solvent A and a 2:3 (vol/vol) acetonitrile/methanol mixture containing 0.1% formic acid as solvent B. A linear gradient elution program was employed as follows: 0 min, 1% B; 1 min, 30% B; 2.5 min, 60% B; 6.5 min, 90% B; 8.5 min, 100% B; 10.7 min, 100% B; 10.8 min, 1% B; and 13 min, 1% B. The column was maintained at a constant temperature of 45°C, with a flow rate of 0.4 mL/min. To ensure sample integrity, all samples were stored at 4°C throughout the analysis, with an injection volume precisely set to 1 μLs.

Mass spectrometry data acquisition was conducted using both full-scan mode (m/z range: 50–1,000) and MSE mode to ensure comprehensive coverage and enhanced fragmentation information. In MSE mode, alternating low- and high-energy scans were performed, enabling simultaneous acquisition of precursor and fragment ion data. The low-energy scans were conducted with a fixed collision energy of 4 eV, while high-energy scans employed a collision energy ramp ranging from 20 to 45 eV. Collision-induced dissociation was facilitated using high-purity argon gas (99.999%), with optimized instrument settings as follows: source temperature set to 115°C, desolvation gas temperature maintained at 450°C, cone voltage at 40 V, desolvation gas flow rate at 900 L/h, a scan interdelay of 0.02 s, and a scan time of 0.2 s.

To ensure data reproducibility and evaluate analytical repeatability, QC samples were systematically injected at regular intervals throughout the analysis, typically after every three sample injections. The QC samples, prepared as pooled extracts from all experimental samples, were used to monitor the relative standard deviations of both retention times and peak areas.

The specifications and details of the primary instruments utilized in this study are available in Supplementary material S1.

### 2.5 Data preprocessing

The raw LC–MS data were processed using Progenesis QI V2.3 software (Nonlinear Dynamics, Newcastle, United Kingdom), incorporating a comprehensive workflow that included baseline filtering, peak detection, integration, retention time correction, peak alignment, and normalization. The data processing pipeline utilized stringent parameters, including a 5% production threshold, 10 ppm product tolerance, and 5 ppm precursor tolerance, to ensure high fidelity and reproducibility. Compound identification was performed through a qualitative analysis using multiple reference databases, including the Human Metabolome Database (HMDB), LipidMaps (V2.3), Metlin, EMDB, PMDB, and a custom in-house database. Accurate mass-to-charge ratios (m/z), secondary fragment patterns, and isotopic distributions were employed as definitive criteria for compound annotation, ensuring precise and reliable metabolite identification.

### 2.6 Statistical analysis

The acquired data underwent rigorous preprocessing to ensure reliability and accuracy. Peaks with more than 50% missing values across groups (ion intensity = 0) were excluded. Zero values were imputed with half of the minimum detected value, and compounds were filtered based on qualitative criteria. Specifically, compounds scoring fewer than 36 points on a 60-point scale were deemed invalid and subsequently removed. Data from both positive and negative ion modes were integrated into a unified data matrix. To evaluate the overall distribution and confirm the stability of the analytical workflow, the consolidated matrix was subjected to principal component analysis (PCA) using the R programming environment.

To identify differential metabolites between experimental groups, we applied orthogonal partial least squares discriminant analysis (OPLS-DA) and partial least squares discriminant analysis (PLS-DA). Model quality was rigorously evaluated through 7-fold cross-validation and 200 response permutation tests to mitigate the risk of overfitting. The variable importance in projection (VIP) scores derived from the OPLS-DA model were utilized to quantify each variable’s contribution to group separation. Metabolites were considered differentially expressed if they met the criteria of a VIP score greater than 1.0 and a *p*-value less than 0.05, determined using a two-tailed Student’s *t*-test.

### 2.7 Kyoto encyclopedia of genes and genomes enrichment analysis

Pathway enrichment analysis of differential metabolites was conducted using their KEGG IDs, leveraging the KEGG database^1^ and the analytical platform developed by Shanghai Oebiotech Co., Ltd.^2^ Enrichment of metabolic pathways was determined using a hypergeometric test, with a significance threshold set at *p* ≤ 0.05. A lower *p*-value indicated a higher degree of significance in the differences observed across metabolic pathways. Detailed calculation formulas and methodologies are provided in Supplementary material S2.

### 2.8 Ethical approval and clinical sample collection

This study was conducted with the approval of the Ethics Committee of the Obstetrics and Gynecology Hospital Affiliated to Tongji University (Ethical Approval Number: 22Y11922400). Clinical samples, including villi and decidual tissues, were collected from January 2024 to May 2024 at Shanghai First Maternity and Infant Hospital (also known as the Obstetrics and Gynecology Hospital Affiliated to Tongji University). The study population consisted of 8 patients diagnosed with RSA (RSA group) and 8 women with normal pregnancies (NC group).

The inclusion criteria for the RSA group comprised patients with a history of two or more consecutive unexplained spontaneous miscarriages occurring prior to 28 weeks of gestation. For the control group, participants were individuals undergoing elective termination of normal pregnancies, carefully matched to the RSA group based on baseline characteristics and with no prior history of spontaneous miscarriage.

Exclusion criteria encompassed any history of infections, reproductive tract abnormalities, endocrine disorders, or other identified causes of miscarriage. Baseline clinical characteristics for both groups are presented in Table 1, with additional details available in Supplementary material S3.

**Table 1:** Clinical characteristics of the RSA group and NC group.

	NC group (Mean ± SEM)	RSA group (Mean ± SEM)	*p*-value	95% CI	
				Down	Up
Count	8	8			
Age (year)	32.25 ± 1.61	32.75 ± 1.16	0.805	−4.761	3.761
BMI (kg/m^2^)	21.53 ± 1.00	22.79 ± 1.18	0.443	−4.738	2.222
Gestational age (week)	7.75 ± 0.53	9.13 ± 0.61	0.110	−3.104	0.354

### 2.9 Western blotting

Total protein was extracted using RIPA lysis buffer (WB6001, Shanghai Wayo Biotechnology, Shanghai, China), and protein concentrations were quantified using the bicinchoninic acid (BCA) method (23,235, Thermo Scientific, Waltham, United States). Equal amounts of protein samples were resolved on SDS-PAGE gels and subsequently transferred onto PVDF membranes (IPVH00010, Millipore, Massachusetts, United States). Membranes were blocked with 5% non-fat milk at room temperature for 1 h, followed by overnight incubation at 4°C with primary antibodies (42,867, Cell Signaling Technology, Boston, United States). The following day, membranes were incubated with secondary antibodies for 1 h at room temperature. Immunoreactive proteins were visualized using the Tanon 5,200 imaging system (Tanon, Shanghai, China).

Grayscale intensities of protein bands were quantified using ImageJ software (NIH, Manassas, MD, United States). The relative expression of target proteins was normalized to internal controls, and mean values along with standard deviations were calculated for each group. Statistical comparisons were performed using two-tailed *t*-tests, with statistical significance defined as *p* < 0.05. All antibodies used in this experiment were obtained from the autophagy antibody kit supplied by Cell Signaling Technology.

### 2.10 Cell culture

HTR8-Svneo cells, a human chorionic trophoblast-derived cell line, were procured from the cell bank of Shanghai First Maternity and Infant Hospital. The cells were maintained in DMEM/F12 medium (C3130-0500, Biological Industries, Kibbutz Beit Haemek, Israel) supplemented with 10% fetal bovine serum and 1% penicillin–streptomycin (15140-122, Grand Island Biological Company, Montana, United States). Cultures were incubated in a humidified atmosphere of 95% air and 5% carbon dioxide at 37°C to ensure optimal growth conditions.

### 2.11 Drug preparation

ASP (Yuanye, Shanghai, China) was dissolved in complete culture medium to prepare a series of concentrations: 0 μg/mL, 0.001 μg/mL, 0.01 μg/mL, 0.1 μg/mL, 1 μg/mL, and 10 μg/mL. The solutions were then sterilized by filtration through a 0.22 μm pore-sized membrane filter to ensure sterility prior to subsequent experiments.

### 2.12 Assessment of cell proliferation capacity

HTR8 cells were seeded into 96-well plates at a density of 3,000 cells per well, with 100 μL of culture medium supplemented with specified concentrations of ASP. Each group included six replicates. After cell adhesion, the Cell Counting Kit-8 (CCK8, MedChemExpress, New Jersey, United States) reagent was added to the wells following the manufacturer’s protocol. A blank control, containing culture medium and CCK8 reagent without cells, was included to account for background absorbance. The optical density (OD) at 450 nm was measured using a microplate reader, with the first measurement recorded as Day 1. Subsequent measurements were performed at 24 h intervals to monitor cell proliferation dynamics.

The net OD was determined by subtracting the OD value of the blank control from that of the experimental wells. Comparative analysis of OD values across groups was conducted, and proliferation curves were generated using GraphPad Prism (version 8.0.2). Statistical significance was assessed via repeated measures analysis, followed by the least significant difference (LSD) method for post-hoc comparisons.

### 2.13 Wound healing assay

To evaluate cell migration, a wound healing assay was performed. Cells were seeded into 6-well plates at a density of 600,000 cells per well and cultured in serum-free medium containing varying concentrations of the tested drug. Once the cell monolayer reached approximately 90% confluence, a sterile 200-μL pipette tip was used to create a uniform, vertical scratch across the well. Detached cells and debris were carefully removed by washing the wells 2–3 times with PBS. Images of the wound area were captured using an inverted microscope (Leica, Wetzlar, Germany) at predefined time intervals. At each time point, the culture medium was replenished to maintain optimal conditions. The wound area was quantified using ImageJ software to assess the rate of wound closure over time.

### 2.14 Transwell migration assay

To assess cell migration, 800 μL of medium containing 20% serum and the respective drug treatment was added to the lower chamber of the Transwell system, which was then placed in a 24-well plate. A total of 200 μL of cell suspension (containing 100,000 cells per well) was seeded into the upper chamber. The system was incubated at 37°C in a humidified incubator with 5% CO_2_ for 16 h. Following incubation, cells that had migrated to the lower surface of the membrane were fixed with 4% paraformaldehyde at room temperature for 20 min. The fixed cells were stained with 0.1% crystal violet solution for 30 min. Non-migrated cells on the upper surface of the membrane were carefully removed using a cotton swab. The membranes were then rinsed with PBS to eliminate excess stain. Migrated cells were visualized and imaged under an inverted microscope for quantitative analysis.

### 2.15 Transwell invasion assay

In contrast to the migration assay, the invasion assay incorporates an additional step to assess cell invasive capabilities. The upper chamber is pre-coated with 100 μL of medium containing 10% Corning matrigel matrix (Corning, New York, United States) to mimic the extracellular matrix. The chamber is incubated at 37°C for 1 h to allow the Matrigel to solidify, after which the supernatant is carefully removed. The subsequent procedures, including cell seeding and incubation, follow the same protocol as described for the migration assay.

### 2.16 Data acquisition and statistical analysis

Image analysis was conducted using ImageJ software, while data visualization and statistical evaluations were performed with GraphPad Prism (version 8.0.2). Results are presented as the mean ± standard error of the mean. Statistical comparisons between groups were performed using one-way analysis of variance. *Post hoc* analyses were carried out using either the LSD or Bonferroni multiple comparison tests to assess the significance of intergroup differences. Statistical significance was defined as follows: *p* < 0.05 (*), *p* < 0.01 (**), or *p* < 0.001 (***).

### 2.17 Transmission electron microscopy

Cells were harvested via centrifugation and promptly fixed in a 2.5% glutaraldehyde solution at 4°C for a minimum of 3 h to preserve cellular structures. Following fixation, samples were washed three times with 0.1 M phosphate buffer and subsequently post-fixed in 1% osmium tetroxide at 4°C for 3 h. The specimens were then subjected to three additional washes with phosphate buffer, sequentially dehydrated in graded ethanol, and embedded in Epon 812 resin to ensure optimal preservation and sectioning quality.

Ultrathin sections, approximately 70 nm in thickness, were prepared using an ultramicrotome (Leica UC6) and carefully mounted onto copper grids coated with formvar support films. The sections were stained with uranyl acetate for 30 min to enhance contrast, followed by counterstaining with lead citrate for 15 min. Finally, the stained sections were visualized and imaged using a transmission electron microscope (Thermo Fisher Talos 120) operated at 120 kV.

## 3 Results

### 3.1 Clinical characteristics of the RSA and NC groups

The diagnostic criteria for RSA were defined according to the ESHRE guidelines (33, 34). The clinical characteristics of participants in the RSA group and the NC group are summarized in Table 1 and Supplementary material S3. No significant differences were observed between the two groups in terms of age (30–34 years), body mass index (BMI, 20–24), or gestational age (7–10 weeks) (*p* > 0.05).

### 3.2 Reduced autophagy levels at the maternal-fetal interface of RSA patients

**Figure 1:**
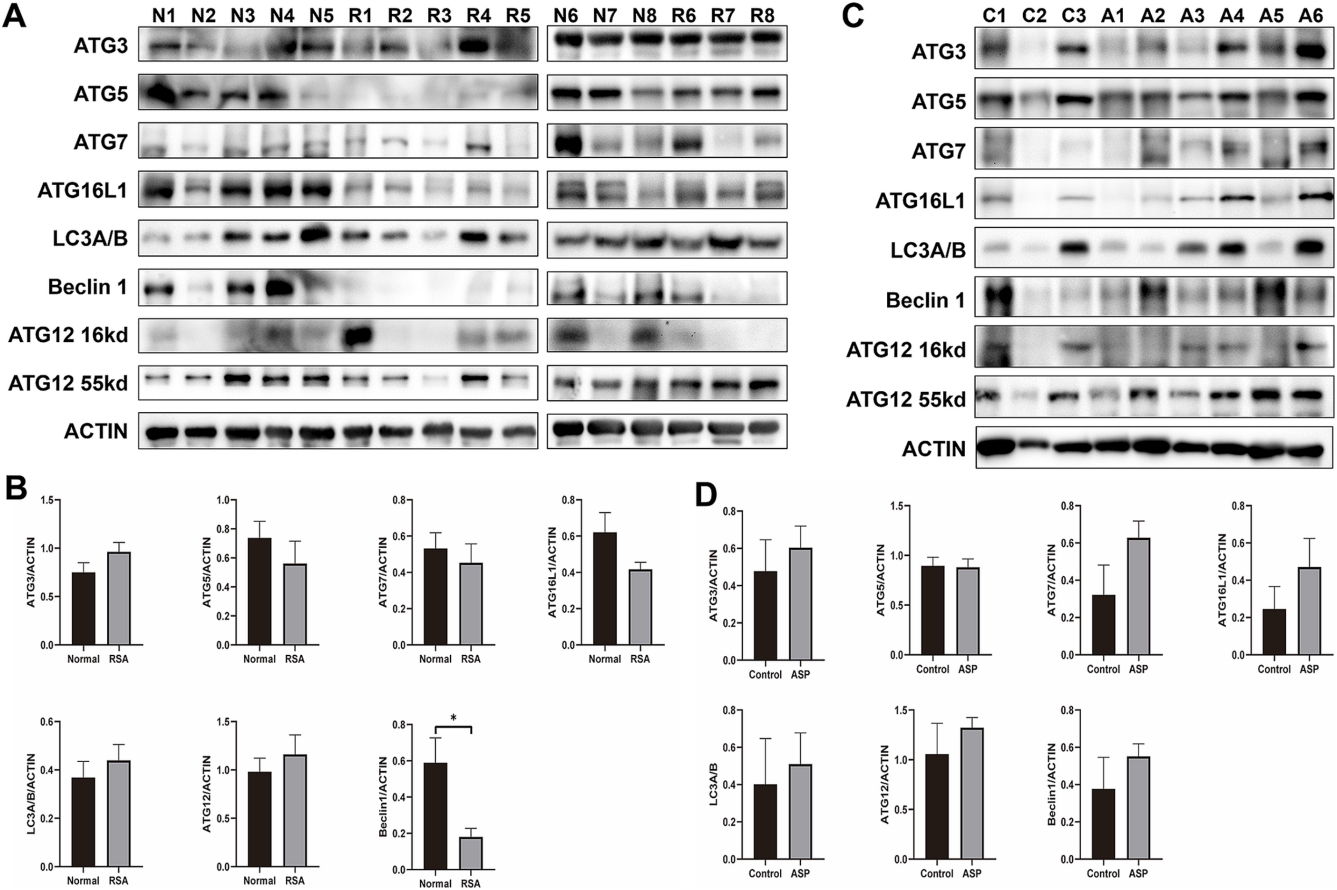
Western blot analysis of autophagy level changes in clinical samples from the RSA group and RSA model mice following ASP intervention. **(A)** Western blot analysis of autophagy-related proteins in samples from the RSA group compared to the Normal group. **(B)** Histogram showing the quantification of Western blot band intensities from **(A)**. **(C)** Western blot analysis of autophagy-related proteins in RSA model mice, comparing the ASP intervention group to the control group. **(D)** Histogram illustrating the quantification of Western blot band intensities from **(C)**.

To investigate autophagy activity at the maternal-fetal interface, we examined decidual tissues from RSA patients and compared them to those of the NC group. As illustrated in Figure 1, autophagy flux was assessed across 8 samples from the NC group and 8 samples from the RSA group. Western blot analysis revealed a significant reduction in the expression levels of autophagy-related proteins, including ATG5, ATG7, and ATG16L, in the RSA group. Notably, Beclin 1 levels were significantly decreased (*p*-value <0.05). Although the expression of several other proteins implicated in the autophagy pathway did not show significant differences, the overall autophagy levels in the RSA group displayed a clear downward trend.

### 3.3 ASP elevate autophagy levels at the maternal-fetal Interface in RSA model mice

Previous studies have demonstrated that ASP promotes autophagy activation (35) and improves outcomes in RSA animal models (36). Furthermore, ASP has been reported to confer protective effects during pregnancy (32). Based on these findings, we hypothesize that ASP may mitigate RSA by activating protective autophagy pathways. Data presented in Supplementary material S4 illustrate the miscarriage status of mice in the RSA model. WB analysis revealed that the expression levels of autophagy-related proteins, including ATG7, ATG16L, and Beclin 1, were significantly elevated in the ASP-treated group compared to the untreated RSA group (Figures 1C,D). Additionally, our previous metabolomic analysis highlighted enrichment of differential metabolites in the autophagy pathway, with pathway activity upregulated in the ASP-treated group relative to controls (Supplementary material S5).

### 3.4 Metabolite profiling of ASP and control groups

**Figure 2:**
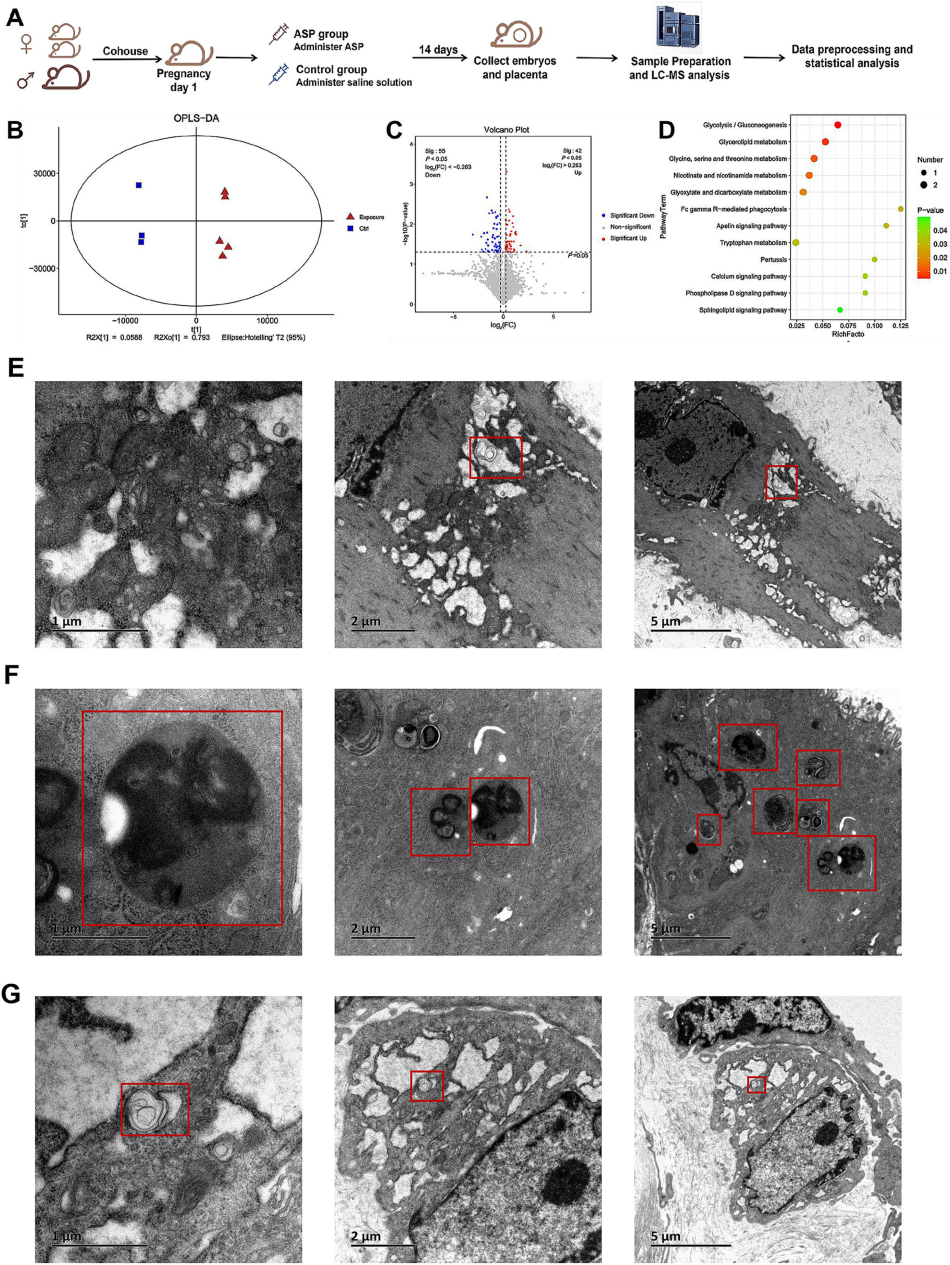
Establishment of the mouse model, metabolomics analysis, and transmission electron microscopy (TEM) images of autophagosomes. **(A)** workflow for constructing the mouse model. Female CBA/J mice and male DBA/2 mice were paired in a 2:1 ratio to establish the RSA model. On the first day of pregnancy, female mice were randomly assigned to either the ASP group or the control group, receiving ASP or an equivalent volume of saline, respectively. Samples were collected after 14 days for subsequent LC–MS analysis and data processing. **(B)** OPLS-DA analysis demonstrates a clear separation between the ASP and control groups. **(C)** Volcano plot of differential metabolites between the ASP and control groups. Each point in the figure represents a metabolite. The x-axis represents the log_2_(FC) value of the comparison between the two groups, while the y-axis represents the −log_10_(*p*-value). Red points indicate metabolites with *p* < 0.05 and fold change (FC) > 1, and blue points indicate metabolites with *p* < 0.05 and FC < 1. Gray points indicate non-significant differences (*p* > 0.05). **(D)** Bubble chart showing KEGG enrichment analysis of selected differential metabolites. **(E)** TEM revealed autophagosomes in the decidual tissues of mice. Autophagosomes in the decidua of mice from the normal control group. **(F)** Autophagosomes in the decidua of mice from the RSA control group. **(G)** Autophagosomes in the decidua of mice from the ASP intervention RSA group.

To investigate the metabolic alterations induced by ASP in the context of RSA, a metabolomic analysis was conducted comparing samples from the ASP-treated and control groups (Figure 2A). An OPLS-DA model revealed a clear and optimized class separation, demonstrating robust model fitting and effectively capturing the metabolic changes induced by ASP exposure (Figure 2B). Among the identified metabolites, 55 were significantly downregulated, and 42 were upregulated in the ASP group compared to the control group (Figure 2C; Supplementary materials S6, S7). Supporting our hypothesis, phosphatidylethanolamine (PE) was prominently altered between the two groups. The differential metabolites identified belong to several chemical classes, including benzene and substituted derivatives, carboxylic acids and derivatives, and fatty acyls, etc. (Supplementary materials S8, S9). Pathway enrichment analysis using the KEGG database highlighted significant enrichment of these metabolites in pathways such as glycolysis/gluconeogenesis, glycerolipid metabolism, glycine, serine, and threonine metabolism, nicotinate and nicotinamide metabolism, glyoxylate and dicarboxylate metabolism, Fc gamma R-mediated phagocytosis, and the Apelin signaling pathway (Figure 2D; Supplementary material S10). Subsequently, autophagosomes were observed by TEM. TEM further provided direct evidence of autophagic activity. Autophagosomes were visualized in the decidual tissues of the normal mouse model (Figure 2E), the RSA mouse model (Figure 2F), and the ASP-treated RSA mouse model (Figure 2G).

### 3.5 ASP enhances proliferation, migration and invasion of human chorionic trophoblast cells

**Figure 3:**
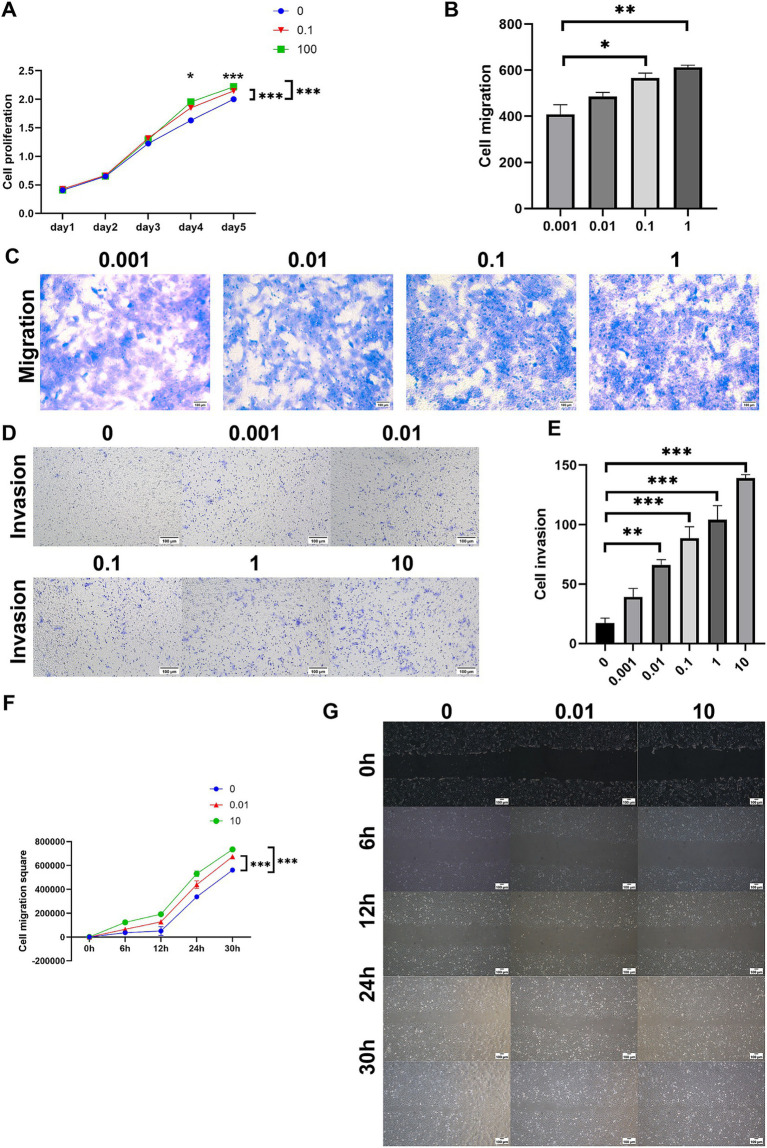
The effects of different concentrations of ASP (0, 0.001, 0.01, 0.1, 1, 10, 100 μg/mL) on the phenotype of HTR8 cells. **(A)** CCK8 assay showing the effect of ASP treatment at concentrations of 0, 0.1, and 100 μg/mL on the proliferation of HTR8 cells. **(B,C)** Transwell migration assay evaluating the effect of ASP treatment at concentrations of 0.001, 0.01, 0.1, and 1 μg/mL on the migration ability of HTR8 cells. **(D,E)** Transwell invasion assay showing the effect of ASP treatment at concentrations of 0, 0.001, 0.01, 0.1, 1, and 10 μg/mL on the invasion ability of HTR8 cells. **(F,G)** Scratch wound assay evaluating the effect of ASP treatment at concentrations of 0, 0.01, and 10 μg/mL on the migration ability of HTR8 cells.

The impact of ASP on the proliferation of HTR8 cells was assessed using the CCK-8 assay, revealing that ASP significantly promoted cell proliferation in a dose-dependent manner (Figure 3A). Consistently, transwell migration and scratch wound healing assays demonstrated a marked enhancement in the migratory capacity of HTR8 cells upon ASP treatment (Figures 3B,C,F,G). Additionally, the transwell invasion assay further confirmed that ASP significantly facilitated the invasive ability of HTR8 cells (Figures 3D,E). Collectively, these findings indicate that ASP serves as a potent enhancer of trophoblast cell proliferation, migration, and invasion, underscoring its potential role in promoting trophoblast function in a dose-dependent manner.

## 4 Discussion

The relationship between RSA and autophagy remains an area of limited investigation, with findings to date presenting inconsistencies. Most research has predominantly focused on autophagy and its upstream and downstream signaling pathways, while metabolic aspects remain underexplored. Some studies have reported elevated autophagy levels in the chorionic tissues of RSA patients (11). Conversely, other investigations have observed a downregulation of autophagy-related genes in the chorion of RSA patients (9, 37, 38), suggesting that suppressed autophagy may lead to aberrant alterations in decidual natural killer (dNK) cell phenotypes, potentially contributing to pregnancy loss (10). Further studies investigating the immune microenvironment at the maternal-fetal interface have highlighted significant upregulation of autophagy-related proteins, such as Beclin 1, LC3B II/I, and BNIP3, in decidual macrophages of RSA patients (8). Impaired decidualization, a key factor influencing RSA, has been associated with reduced autophagy levels and disrupted uterine decidualization in RSA patients (39). Moreover, preclinical research indicates that hypericin, a bioactive compound, exerts protective effects against abortion in a rat model by enhancing autophagy (40). These findings collectively underscore the complex and multifaceted role of autophagy in RSA. However, the precise interplay between autophagy, immune regulation, and metabolism at the maternal-fetal interface remains to be fully elucidated. Further studies are warranted to clarify the mechanistic links between autophagy and RSA pathogenesis, which may pave the way for novel therapeutic strategies targeting this pathway.

Our experimental findings reveal a significant reduction in Beclin-1 levels in RSA patients compared to those with normal pregnancies, accompanied by a decreasing trend in ATG5, ATG7, and ATG16L expression. The autophagy pathway is initiated by the unc-51-like autophagy-activating kinase (ULK) complex, which orchestrates upstream signals to activate downstream processes. Beclin-1, a pivotal component of the autophagy-specific vacuolar protein sorting 34 (VPS34) complex I, plays a critical role in catalyzing the production of phosphatidylinositol-3-phosphate (PI3P) (PI3P). The generation of PI3P facilitates the recruitment of autophagy-related machinery, including the ATG16L1-ATG5-ATG12 complex, ATG3, and ATG7. These components work synergistically to conjugate ATG8 family members—encompassing the LC3 and GABARAP subfamilies—with PE, a key step in promoting autophagosome maturation and subsequent autophagic flux (12, 41).

Therefore, our findings indicate that autophagy levels at the maternal-fetal interface are diminished in RSA patients compared to those with normal pregnancies, aligning with previously reported observations (9, 37, 38). Importantly, in the RSA mouse model, treatment with ASP partially restored autophagy activity, suggesting a potential mechanism by which ASP confers protective effects in RSA. Beyond its influence on autophagy, ASP has demonstrated broader benefits in pregnancy-related contexts. For example, ASP has been shown to mitigate iron-deficiency anemia in pregnant rats by modulating the hepcidin-FPN1 axis (32). Furthermore, Angelica sinensis extracts, such as Ligustilide, have been reported to enhance pregnancy outcomes by improving endometrial receptivity and promoting angiogenesis within the endometrium (42). Additionally, Angelica sinensis has been implicated in alleviating metabolic disturbances in abortion-prone mice through the regulation of glycerolipid metabolism and has been shown to exert immunomodulatory effects (29, 43).

Simultaneously, ASP has demonstrated the ability to regulate autophagy through diverse signaling pathways. In the context of osteoarthritis, ASP has been reported to induce autophagy via activation of the ERK1/2 pathway (35). Similarly, ASP can mitigate chemotherapy-induced hepatotoxicity by enhancing autophagy through the MEK/ERK signaling cascade (44). Additionally, studies in a rat model of idiopathic pulmonary fibrosis revealed that Angelica sinensis exerts its autophagy-inducing effects via modulation of the mammalian target of rapamycin (mTOR) pathway (45).

In summary, ASP provide a degree of protection during pregnancy and can influence autophagy levels through different signaling pathways. Despite extensive evidence supporting the protective role of Angelica sinensis in pregnancy, research specifically investigating the contribution of ASP in miscarriage remains limited. The phytochemical composition of Angelica sinensis is highly complex, encompassing various bioactive compounds such as ASP, ligustrazine, laurene, ferulic acid, and vanillic acid (46). This complexity underscores the need for targeted studies to identify whether ASP represents the primary active component responsible for its therapeutic effects and to elucidate the molecular mechanisms involved. Our findings provide preliminary evidence suggesting that ASP may mitigate RSA by activating autophagy. However, further comprehensive investigations are required to validate these observations and explore the precise mechanisms underlying this protective effect.

Metabolomics has emerged as a robust and unbiased analytical approach, offering a comprehensive overview of an individual’s metabolic profile (47). In this study, a metabolomic analysis was performed to compare the metabolic profiles of the ASP-treated group and the control group, revealing significant differences in PE levels between the two. PE plays a critical role in the autophagy pathway, serving as an essential lipid for the conjugation of LC3-I, facilitating its conversion into the autophagosome-associated form, LC3-II (48). These findings highlight the potential mechanistic link between ASP treatment and autophagy regulation.

KEGG pathway analysis highlighted significant alterations in metabolic pathways, including Glycolysis/Gluconeogenesis, Glycerolipid metabolism, and Glycine, serine, and threonine metabolism. Glycolysis/Gluconeogenesis has been implicated in impaired decidualization in pregnant rats (49) and is associated with defective trophoblast invasion in preeclampsia patients, as previously reported (50). Similarly, disruptions in Glycerolipid metabolism have been identified as potential biomarkers for idiopathic infertility in *in vitro* fertilization (IVF) patients (51) and are linked to an increased risk of gestational diabetes in pregnant women (52). Consistent with our findings, metabolomic analyses of plasma from RSA patients have also revealed changes in Glycolysis/Gluconeogenesis and Glycerolipid metabolism (53). Both pathways have been further associated with preterm birth (54) and gestational diabetes risk (55). These studies collectively underscore the critical impact of metabolic dysregulation on pregnancy, highlighting the intricate connection between altered metabolic states and pregnancy complications.

However, there is a lack of relevant research when it comes to trends in the levels of specific metabolites within these pathways or whether intervention leads to reversals of these trends, particularly in RSA. Our findings reflect the possible metabolic mechanisms through which ASP exerts protective effects against RSA by regulating autophagy, providing a comprehensive overview of the metabolic profile changes induced by ASP at the maternal-fetal interface. Although these discoveries do not delve into deeper metabolic explorations, they lay a foundation and perspective for further investigation into the pathogenesis of RSA and its treatment.

However, this study has some limitations. Firstly, although our study initially found that ASP exerts protective effects on RSA by regulating autophagy, we did not further conduct dynamic validation to comprehensively observe autophagic flux. Instead, we focused only on autophagy levels at specific time points. This verification is crucial for establishing the reliability and applicability of the research findings. In future projects, we plan to perform additional experiments to observe autophagic flux in RSA and ASP intervention. These experiments may include observing LC3-labeled cells under a fluorescence microscope, using lysosome-specific fluorescent dyes to assess lysosomal function, measuring sequestosome 1 levels at different time points after intervention, and combining lysosomal inhibitors to validate autophagic flux activity. Secondly, the sample size in our study is relatively small, with only 8 participants in each clinical group, and the mouse experiments included only 6 and 3 samples from the ASP and control groups, respectively. This limitation restricts the generalizability of the findings and may affect the applicability of the results. Before ASP can be considered a protective factor for RSA, more in-depth validation in larger independent cohorts is necessary. Although our current metabolomics sequencing results indicate that PE is an important differential metabolite and that ASP affects key metabolic pathways, we have not yet conducted further experiments to explore how the metabolic changes induced by ASP specifically contribute to the pathological mechanisms of RSA. In future studies, we plan to exogenously add PE and other key metabolites to evaluate their effects on cell functions. Furthermore, in an RSA animal model with ASP intervention, we aim to measure the activity of key metabolic enzymes and lipid metabolic enzymes in critical pathways to investigate whether ASP improves RSA by regulating these enzyme activities and influencing metabolic pathways. At the same time, we will utilize specific inhibitors or gene knockout or knockdown methods to study the expression of key metabolic enzymes in cell models and assess their effects on cellular functions and the autophagy pathway.

## 5 Conclusion

In summary, we studied the levels of autophagy in the maternal-fetal interface of RSA patients and healthy pregnant individuals, and examined the changes in autophagy levels in ASP-treated RSA model mice, followed by a metabolomic analysis and cell phenotype assays. Our findings suggest that ASP may exert protective effects against RSA by activating autophagy while influencing pathways such as Glycolysis/Gluconeogenesis, Glycerolipid metabolism, and Glycine, serine, and threonine metabolism. However, further research and validation are necessary. Our results may provide insights for exploring the pathogenesis of RSA and offer evidence for the therapeutic effects of ASP in treating RSA.

## Data Availability

The original contributions presented in the study are included in the article/Supplementary material, further inquiries can be directed to the corresponding authors.

## Footnotes

^1^https://www.kegg.jp/

^2^https://cloud.oebiotech.cn/task/

## Supplementary material

Supplementary material S1 The main instruments used during the LC-MS process, along with their models/specifications and manufacturers.

Supplementary material S2 Calculation method for *P*-value in KEGG analysis.

Supplementary material S3 Detailed clinical information of RSA group and NC group.

Supplementary material S4 Miscarriage status in normal control group and RSA model group.

Supplementary material S5 Bubble chart and bar chart of metabolic pathway KEGG enrichment analysis. **(A)** and **(C)** Bubble chart and bar chart of pathways with *P*-values less than 0.05. **(B)** and **(D)** Bubble chart and bar chart of top 20 pathways ranked by -lg(*P*-value). **(E)** and **(G)** Bubble chart and bar chart of pathways with *P*-values less than 0.05 and upregulated. **(F)** and **(H)** Bubble chart and bar chart of top 20 pathways ranked by -lg(*P*-value) and upregulated. **(I)** and **(J)** Bubble chart and bar chart of top 20 pathways ranked by -lg(*P*-value), downregulated and with *P*-values less than 0.05.

Supplementary material S6 Summary of differential metabolites.

Supplementary material S7 Heatmap of hierarchical clustering of the top 50 significant differential metabolites ranked by variable importance in projection.

Supplementary material S8 Pie charts showing the classification of metabolites. **(A)** Pie chart of metabolite classes. **(B)** Pie chart of metabolite subclasses. **(C)** Pie chart of metabolite superclasses.

Supplementary material S9 Data matrix of classification pie charts.

Supplementary material S10 Summary table of metabolite pathways.
