# Supplementary material for: Angelica sinensis polysaccharide as potential protectants against recurrent spontaneous abortion: focus on autophagy regulation
Source: Front Med (Lausanne). 2025 Jan 15;12:1522503. doi: 10.3389/fmed.2025.1522503 (PMC11774876; doi:10.3389/fmed.2025.1522503)
Supplement: Supplementary material S1 — The main instruments used during the LC-MS process, along with their models/specifications and manufacturers. [file Supplementary_file_1.zip › Metabolomics sequencing data FC1.2/3.数据矩阵/Pie_Graph/piechart-Class1.pdf]

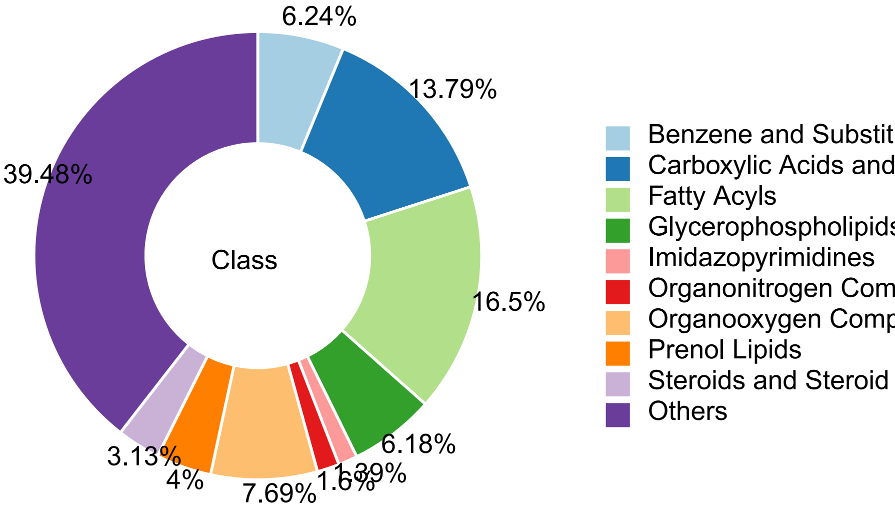
6.24%
13.79%
16.5%
6.18%
1.39%
1.6%
7.69%
4%
3.13%
39.48%
Class
Benzene and Substit
Carboxylic Acids and
Fatty Acyls
Glycerophospholipids
Imidazopyrimidines
Organonitrogen Com
Organooxygen Comp
Prenol Lipids
Steroids and Steroid
Others
