# Supplementary figures and images for: Angelica sinensis polysaccharide as potential protectants against recurrent spontaneous abortion: focus on autophagy regulation
Source: Front Med (Lausanne). 2025 Jan 15;12:1522503. doi: 10.3389/fmed.2025.1522503 (PMC11774876; doi:10.3389/fmed.2025.1522503)

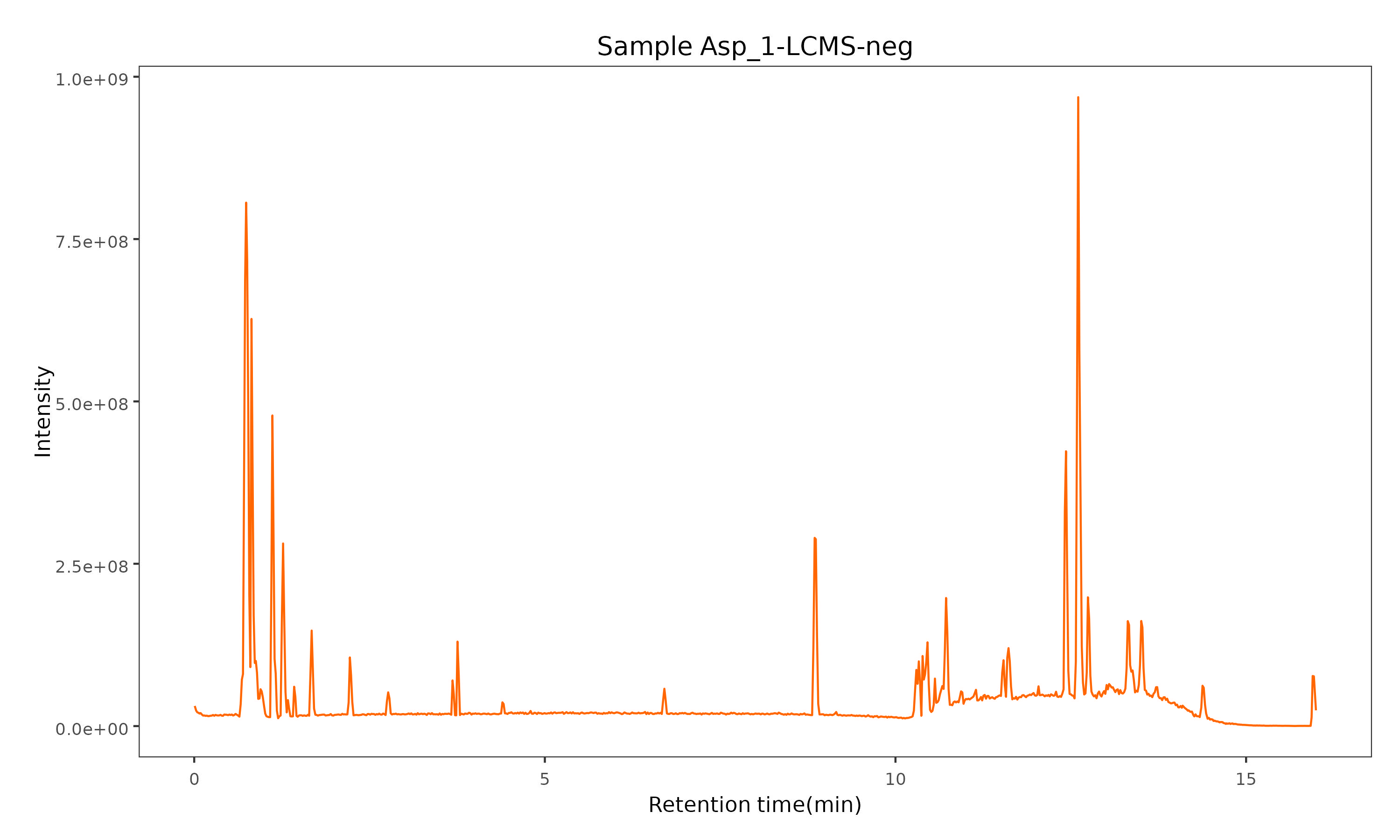

Supplement: Supplementary material S1 — The main instruments used during the LC-MS process, along with their models/specifications and manufacturers. [file Supplementary_file_1.zip › Metabolomics sequencing data FC1.2/1.基峰图/Asp_1-LCMS-neg-BPC.jpg]

Sample Asp\_1-LCMS-neg

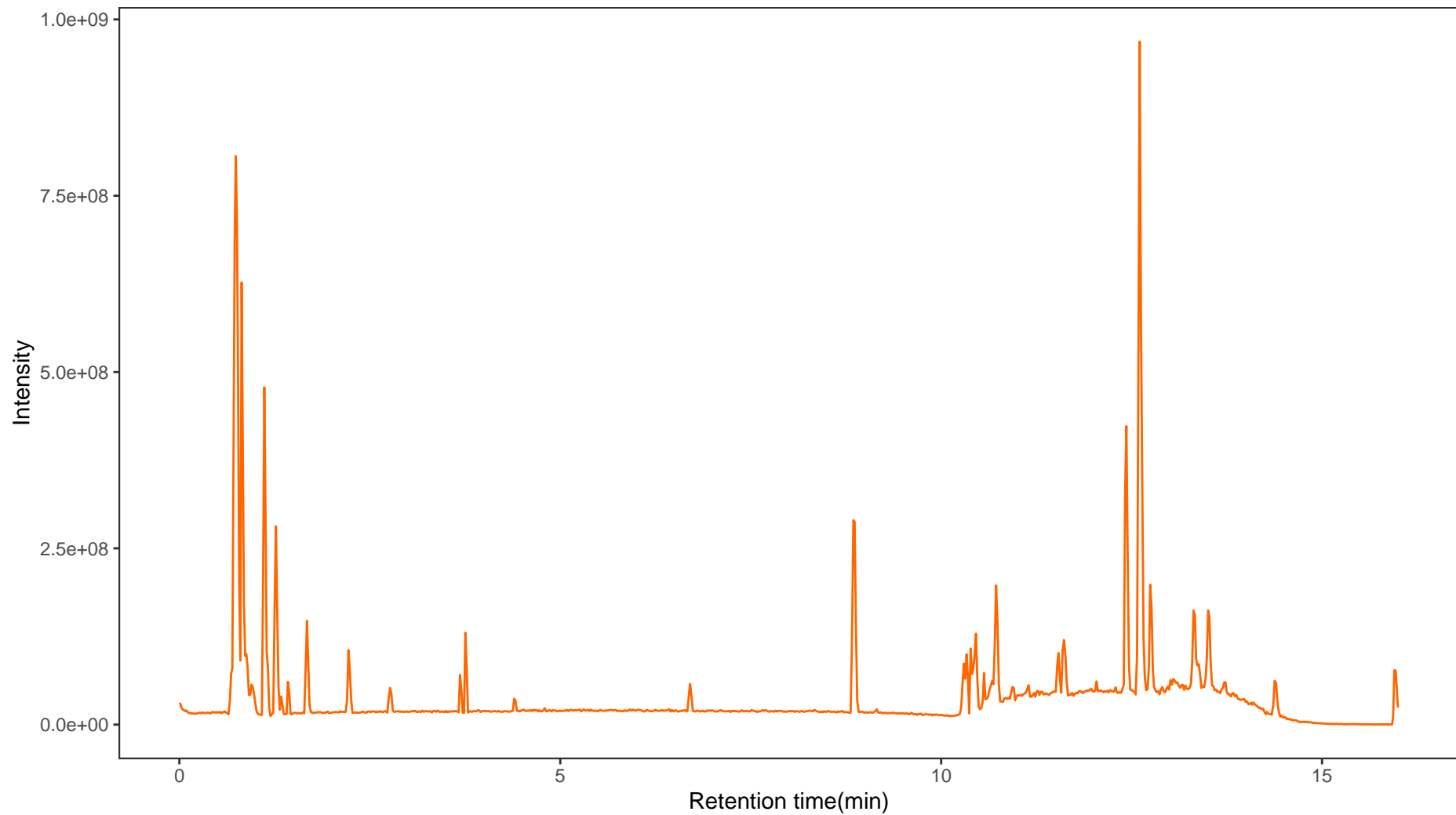

Supplement: Supplementary material S1 — The main instruments used during the LC-MS process, along with their models/specifications and manufacturers. [file Supplementary_file_1.zip › Metabolomics sequencing data FC1.2/1.基峰图/Asp_1-LCMS-neg-BPC.pdf]

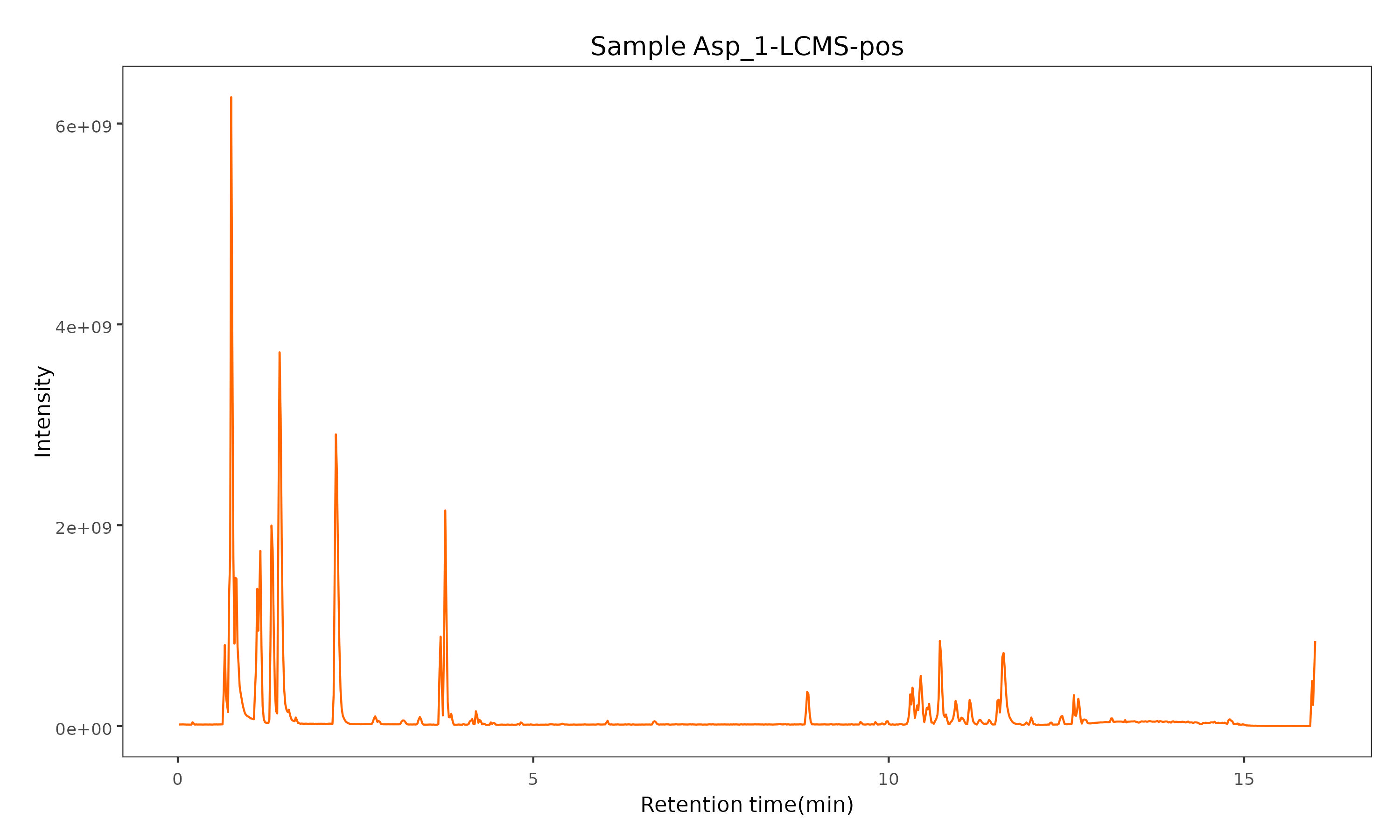

Supplement: Supplementary material S1 — The main instruments used during the LC-MS process, along with their models/specifications and manufacturers. [file Supplementary_file_1.zip › Metabolomics sequencing data FC1.2/1.基峰图/Asp_1-LCMS-pos-BPC.jpg]

Sample Asp\_1-LCMS-pos

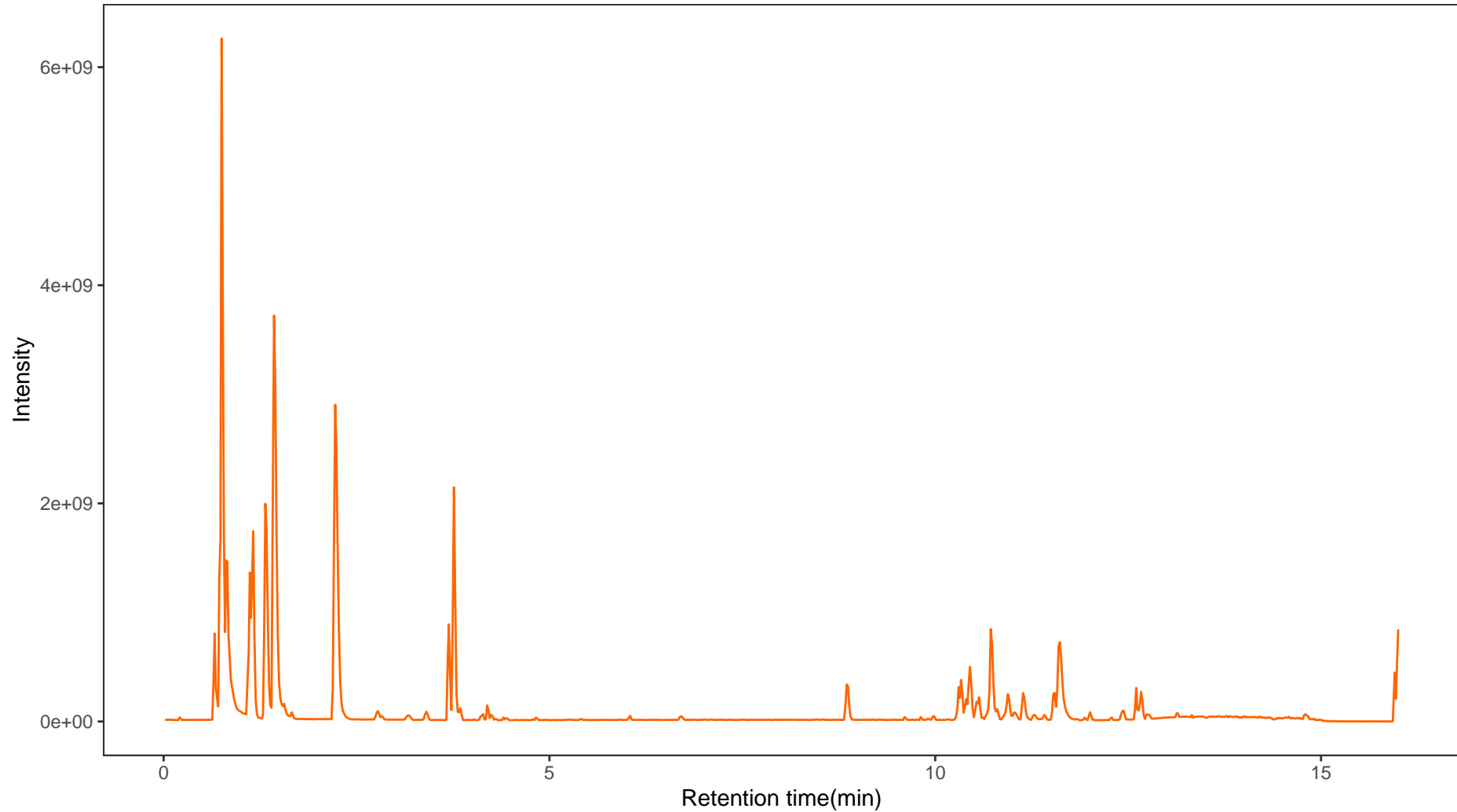

Supplement: Supplementary material S1 — The main instruments used during the LC-MS process, along with their models/specifications and manufacturers. [file Supplementary_file_1.zip › Metabolomics sequencing data FC1.2/1.基峰图/Asp_1-LCMS-pos-BPC.pdf]

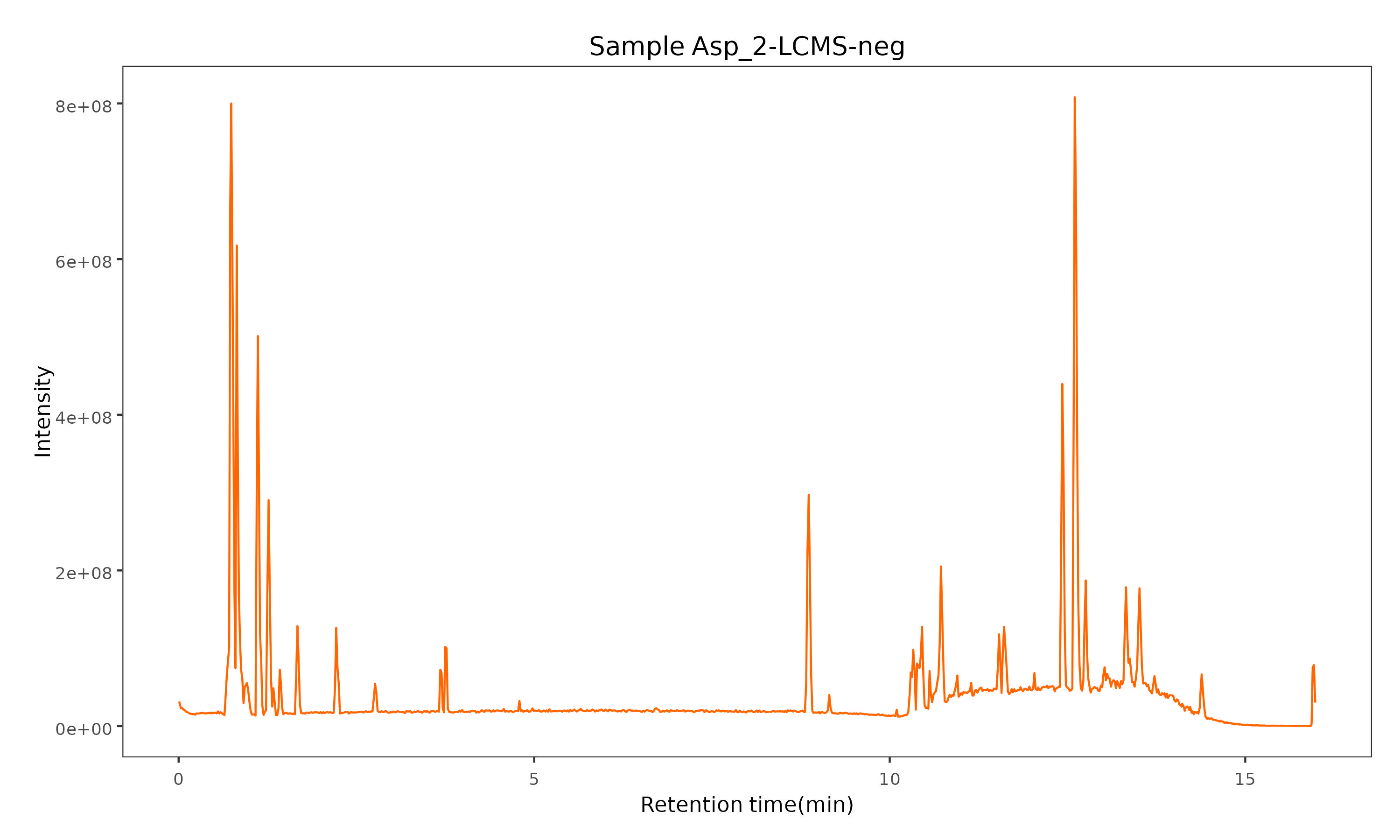

Supplement: Supplementary material S1 — The main instruments used during the LC-MS process, along with their models/specifications and manufacturers. [file Supplementary_file_1.zip › Metabolomics sequencing data FC1.2/1.基峰图/Asp_2-LCMS-neg-BPC.jpg]

Sample Asp\_2-LCMS-neg

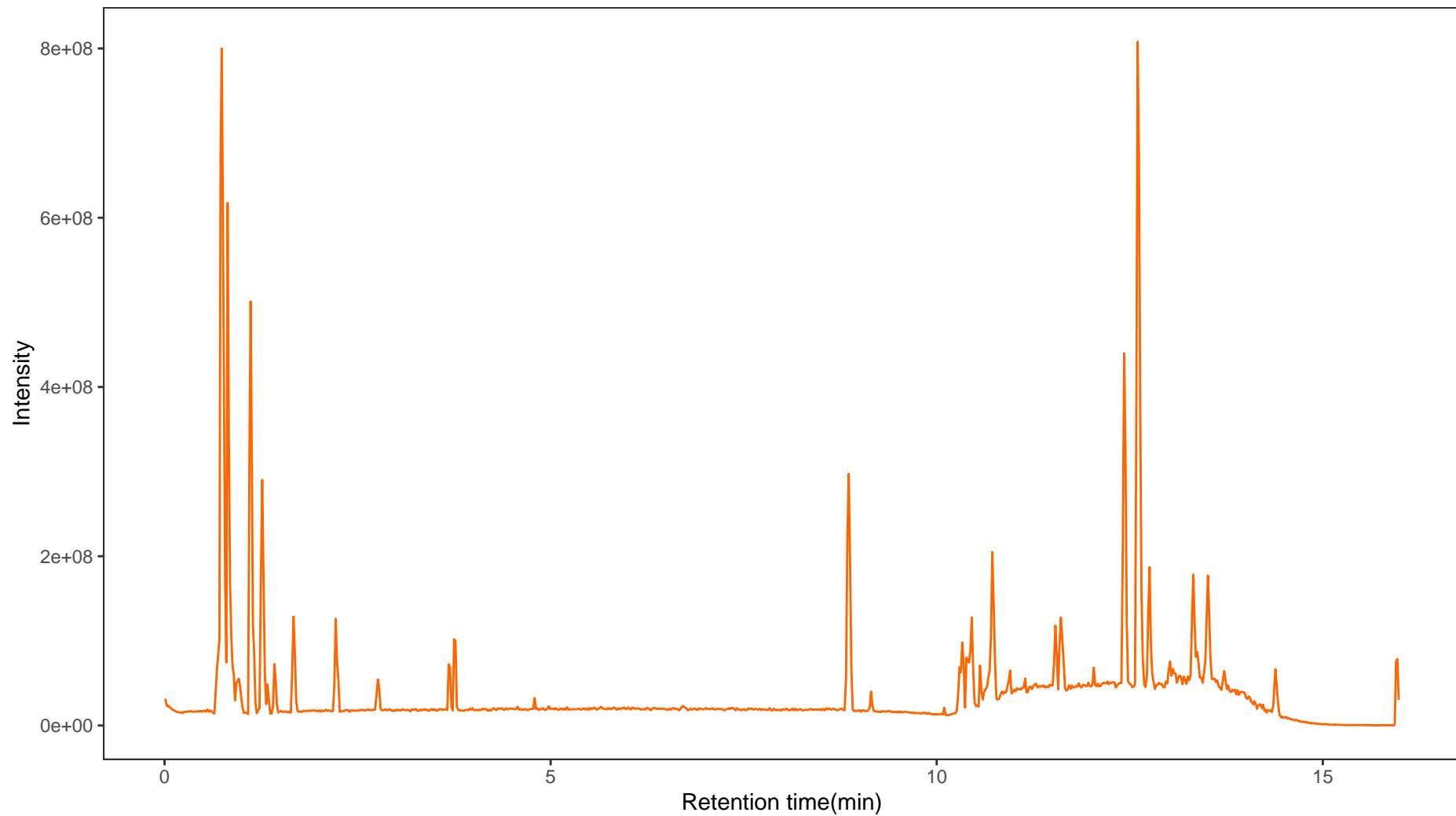

Supplement: Supplementary material S1 — The main instruments used during the LC-MS process, along with their models/specifications and manufacturers. [file Supplementary_file_1.zip › Metabolomics sequencing data FC1.2/1.基峰图/Asp_2-LCMS-neg-BPC.pdf]

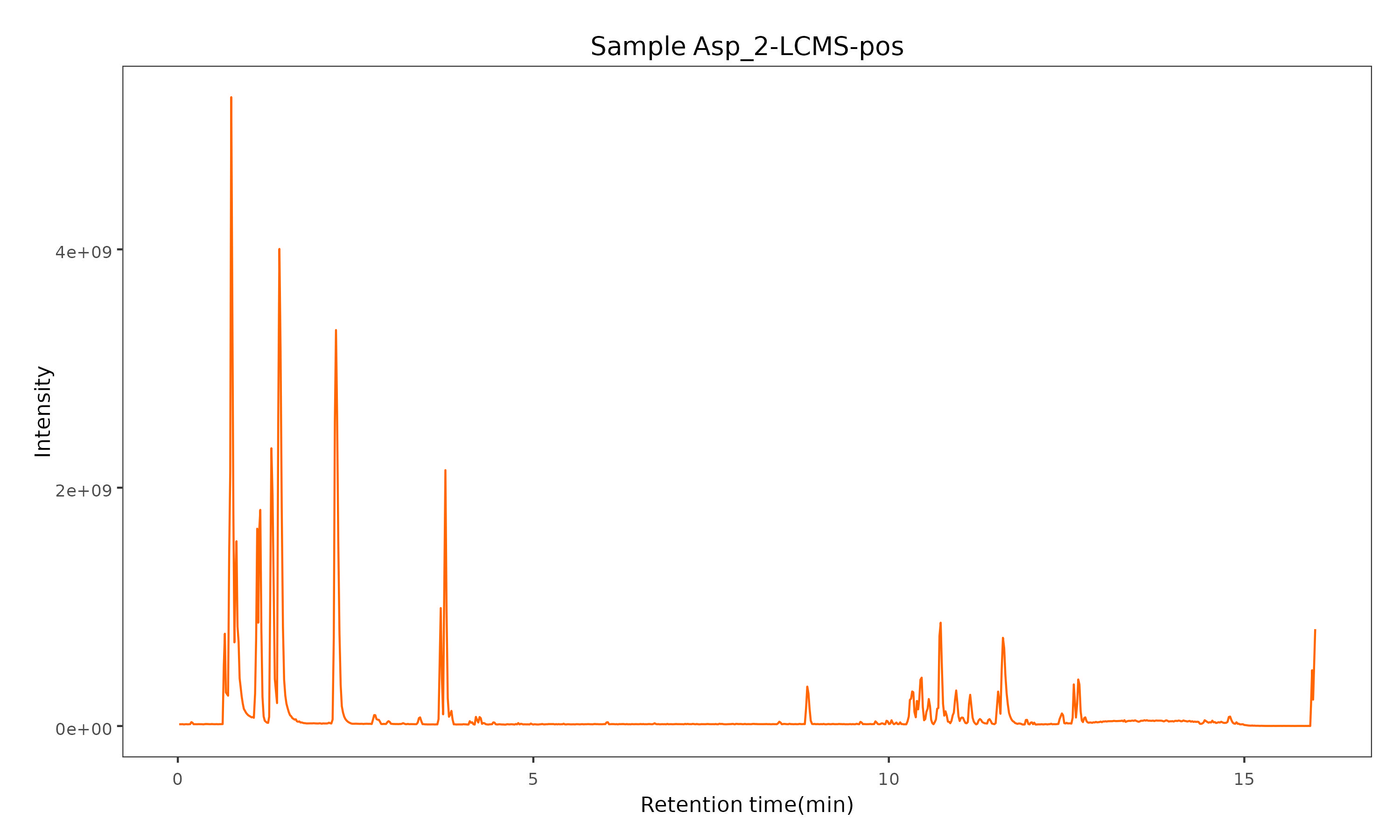

Supplement: Supplementary material S1 — The main instruments used during the LC-MS process, along with their models/specifications and manufacturers. [file Supplementary_file_1.zip › Metabolomics sequencing data FC1.2/1.基峰图/Asp_2-LCMS-pos-BPC.jpg]

Sample Asp\_2-LCMS-pos

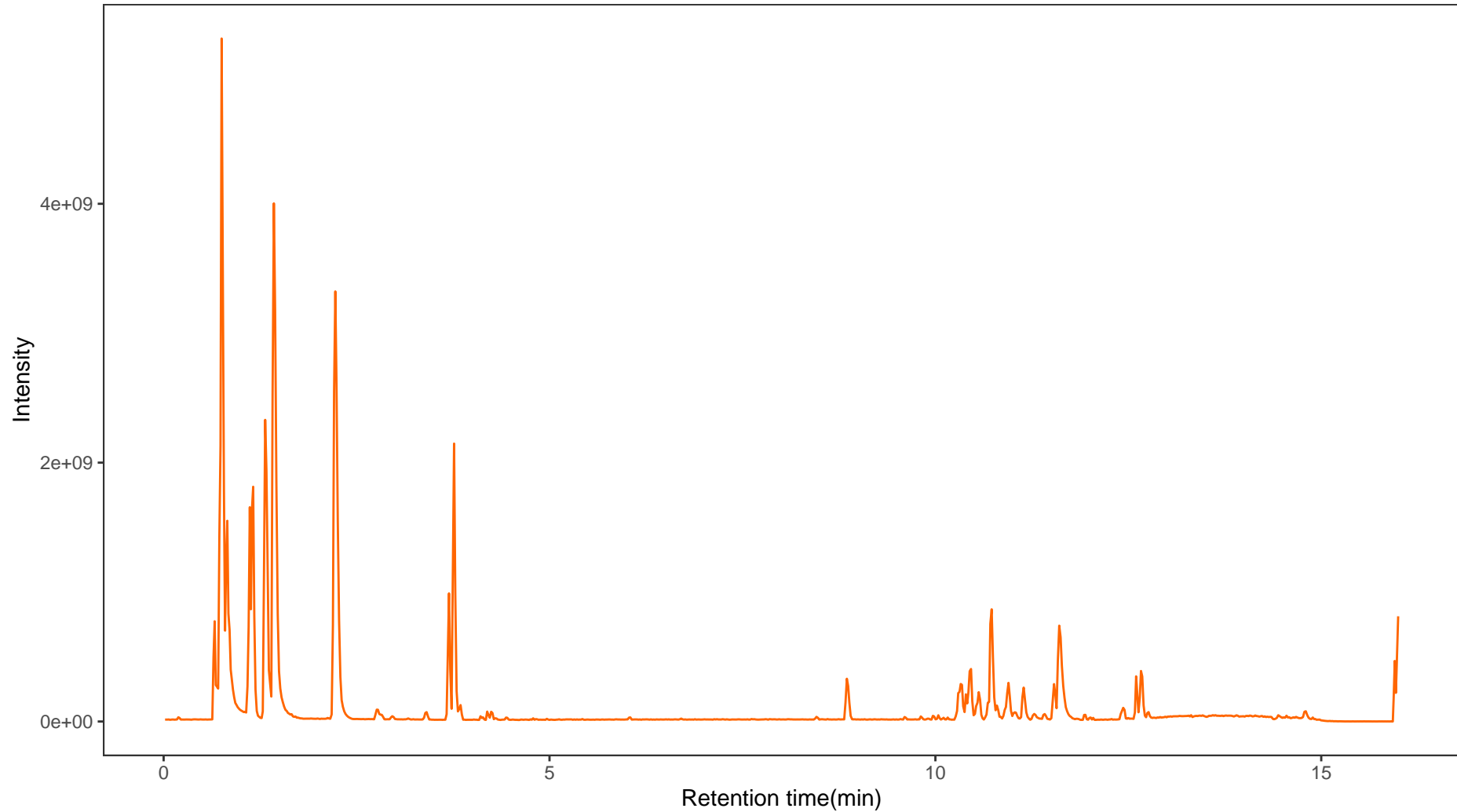

Supplement: Supplementary material S1 — The main instruments used during the LC-MS process, along with their models/specifications and manufacturers. [file Supplementary_file_1.zip › Metabolomics sequencing data FC1.2/1.基峰图/Asp_2-LCMS-pos-BPC.pdf]

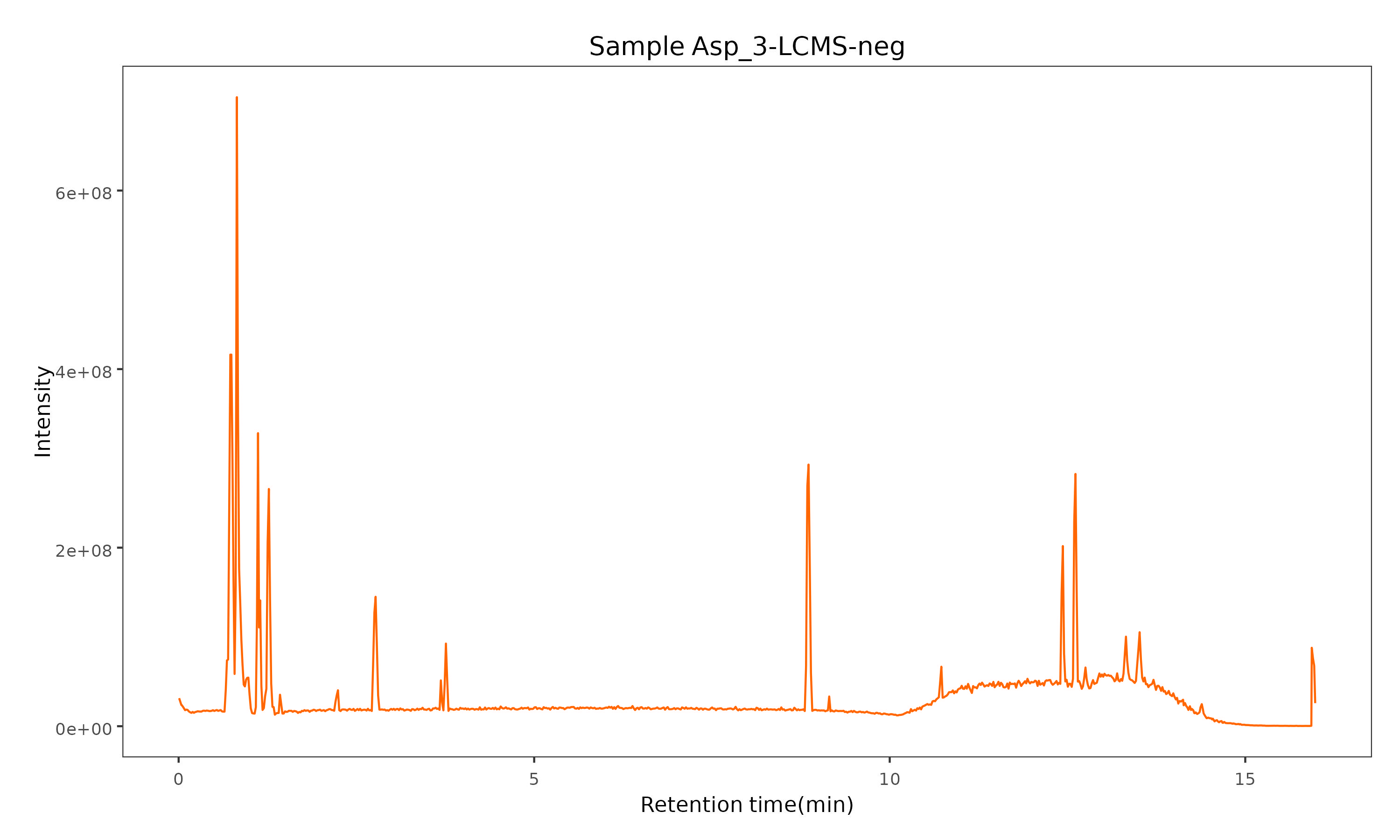

Supplement: Supplementary material S1 — The main instruments used during the LC-MS process, along with their models/specifications and manufacturers. [file Supplementary_file_1.zip › Metabolomics sequencing data FC1.2/1.基峰图/Asp_3-LCMS-neg-BPC.jpg]

Sample Asp\_3-LCMS-neg

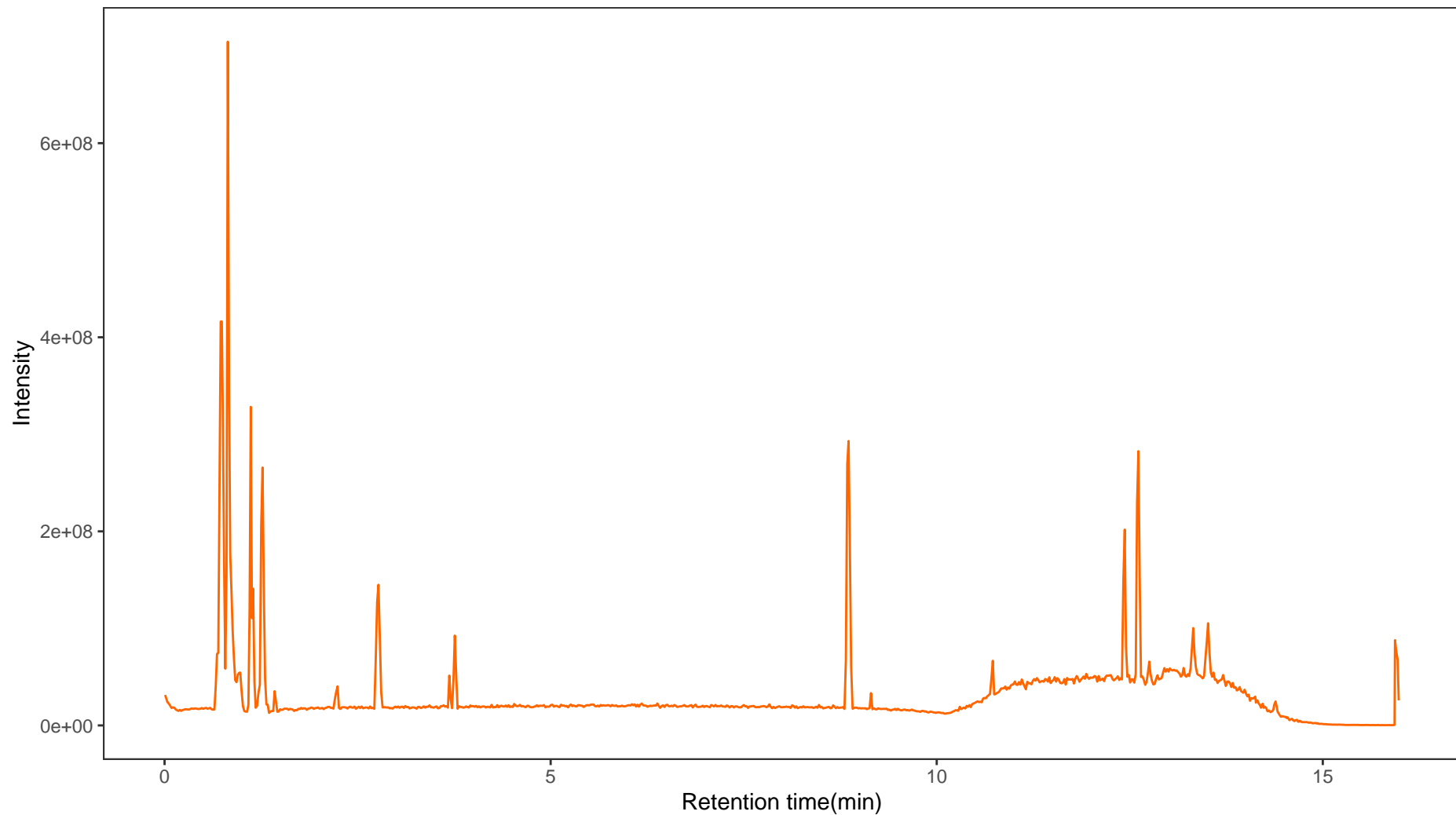

Supplement: Supplementary material S1 — The main instruments used during the LC-MS process, along with their models/specifications and manufacturers. [file Supplementary_file_1.zip › Metabolomics sequencing data FC1.2/1.基峰图/Asp_3-LCMS-neg-BPC.pdf]

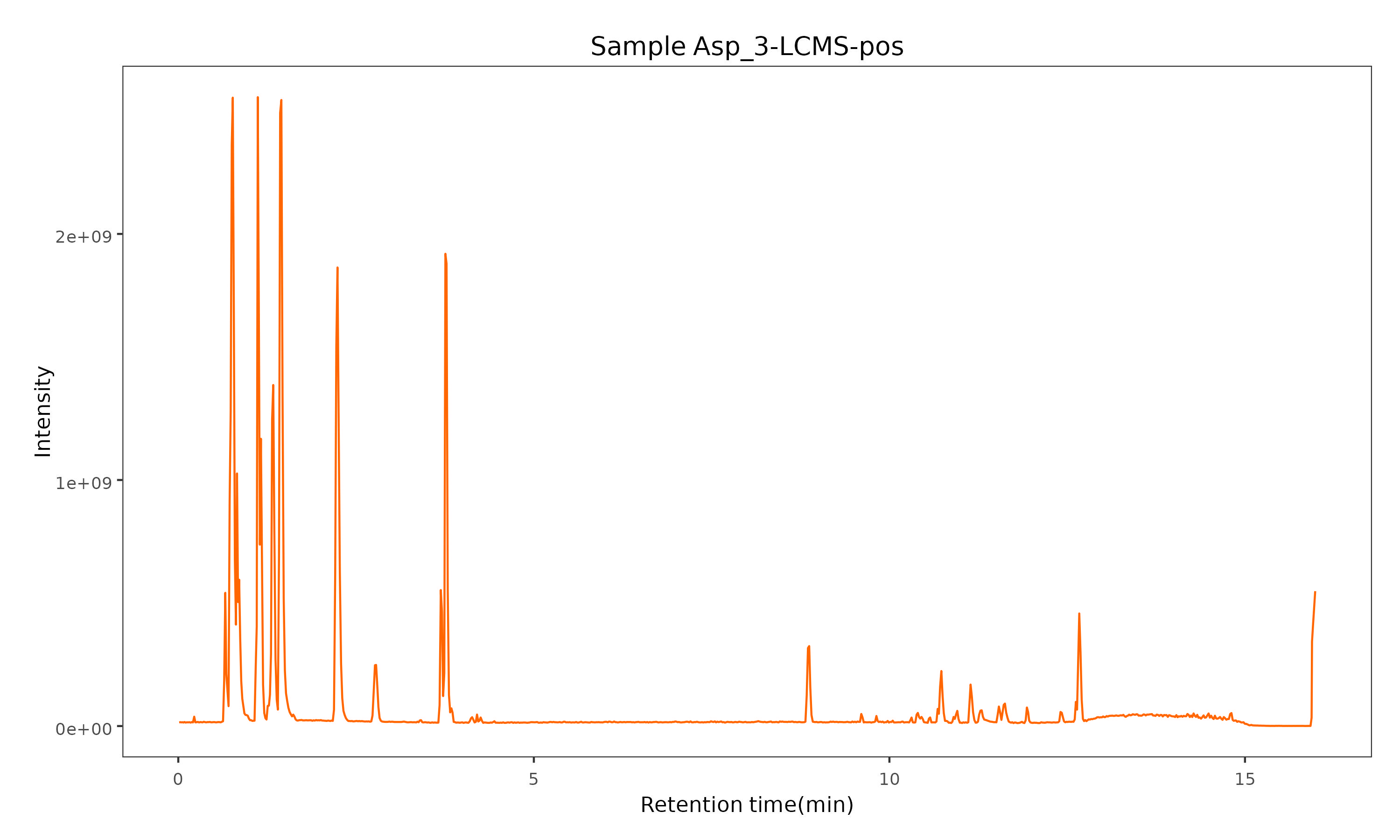

Supplement: Supplementary material S1 — The main instruments used during the LC-MS process, along with their models/specifications and manufacturers. [file Supplementary_file_1.zip › Metabolomics sequencing data FC1.2/1.基峰图/Asp_3-LCMS-pos-BPC.jpg]

Sample Asp\_3-LCMS-pos

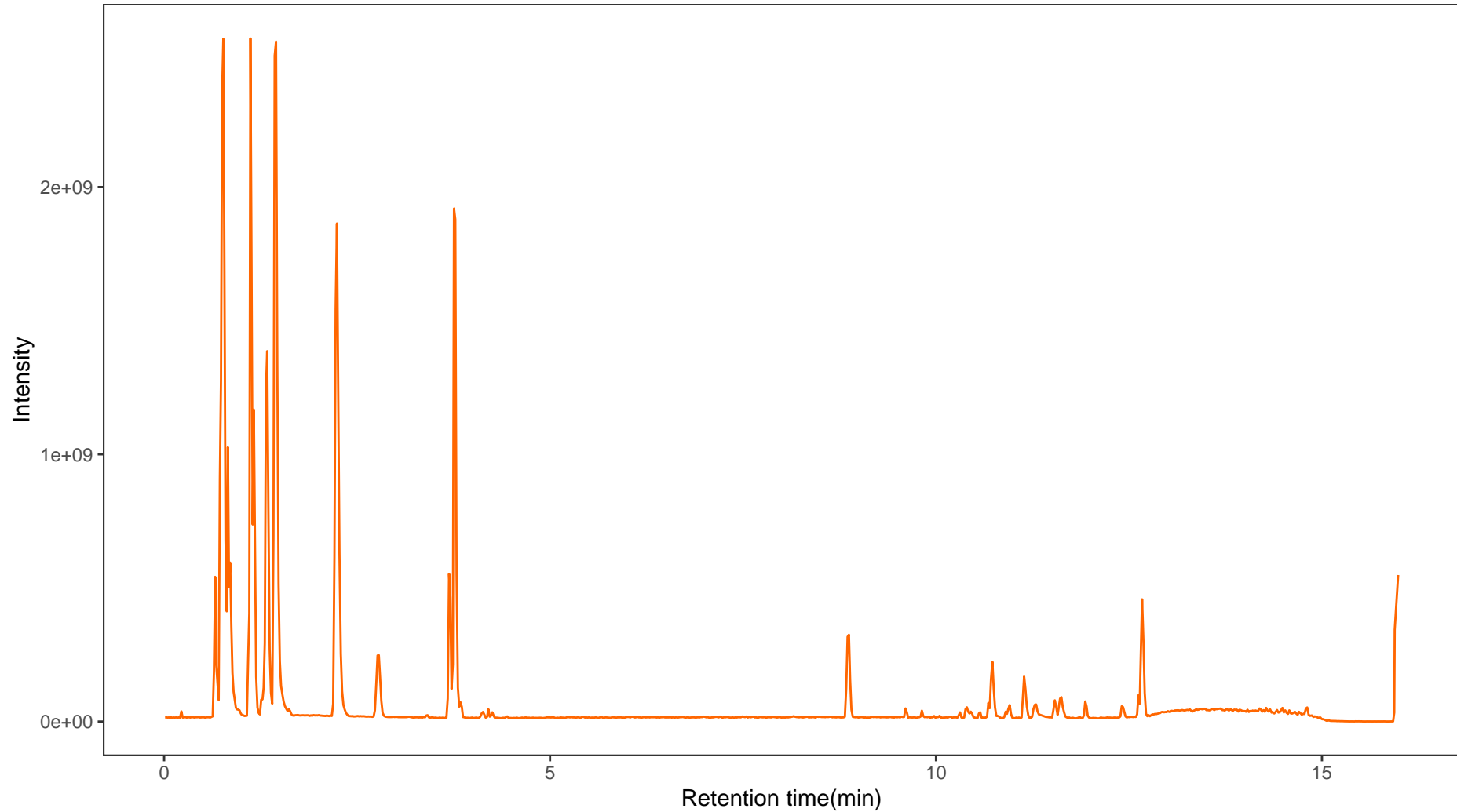

Supplement: Supplementary material S1 — The main instruments used during the LC-MS process, along with their models/specifications and manufacturers. [file Supplementary_file_1.zip › Metabolomics sequencing data FC1.2/1.基峰图/Asp_3-LCMS-pos-BPC.pdf]

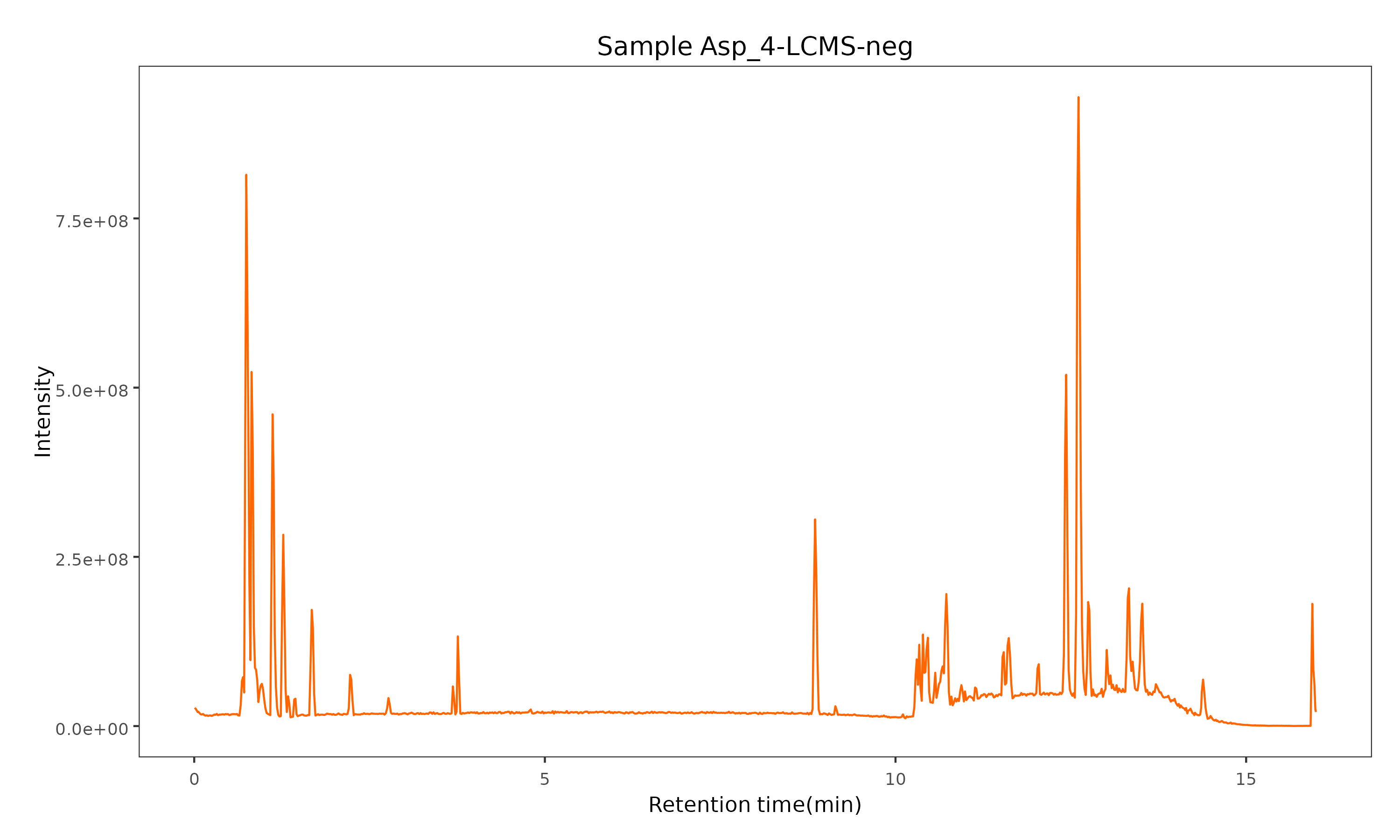

Supplement: Supplementary material S1 — The main instruments used during the LC-MS process, along with their models/specifications and manufacturers. [file Supplementary_file_1.zip › Metabolomics sequencing data FC1.2/1.基峰图/Asp_4-LCMS-neg-BPC.jpg]

Sample Asp\_4-LCMS-neg

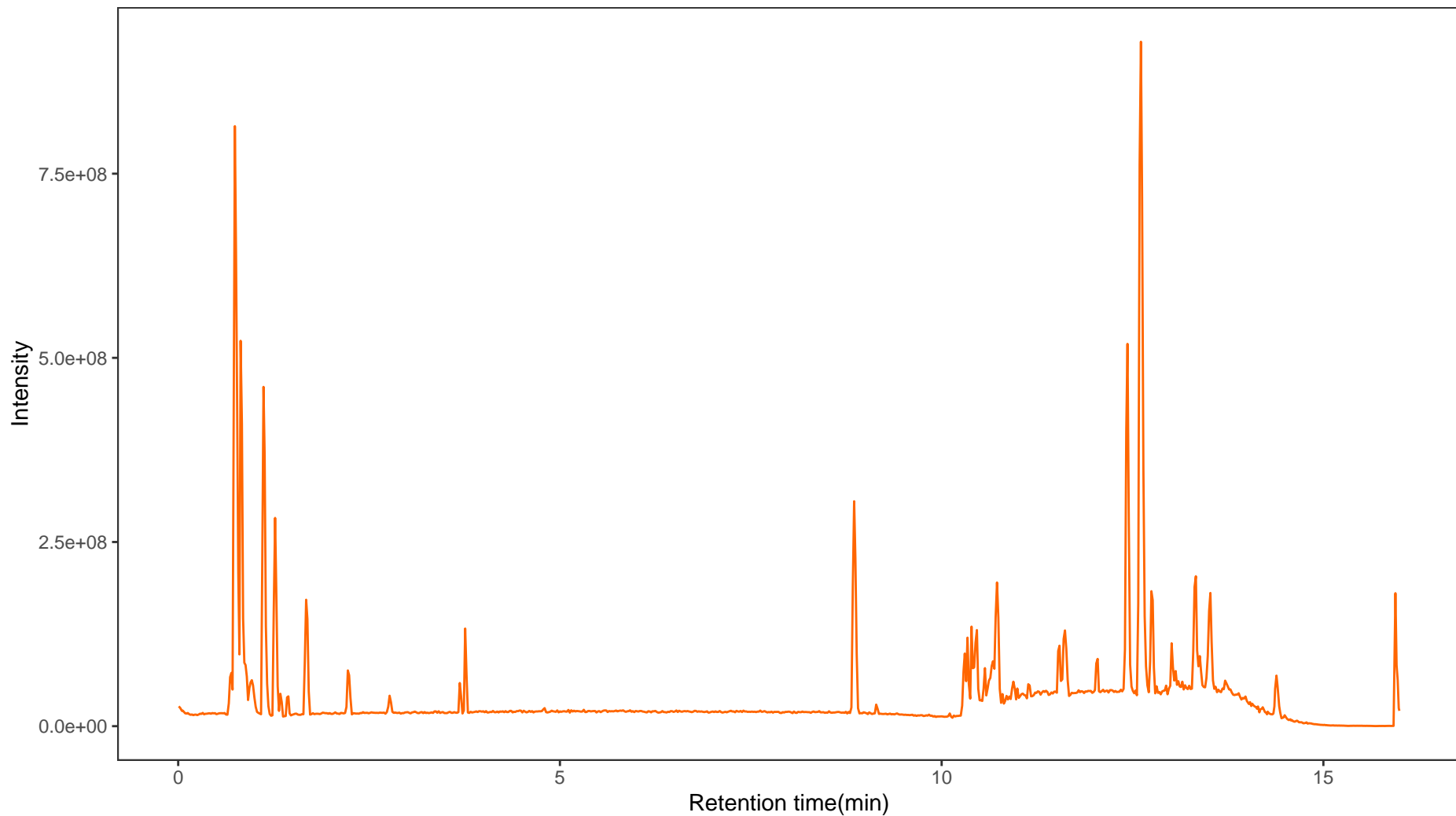

Supplement: Supplementary material S1 — The main instruments used during the LC-MS process, along with their models/specifications and manufacturers. [file Supplementary_file_1.zip › Metabolomics sequencing data FC1.2/1.基峰图/Asp_4-LCMS-neg-BPC.pdf]

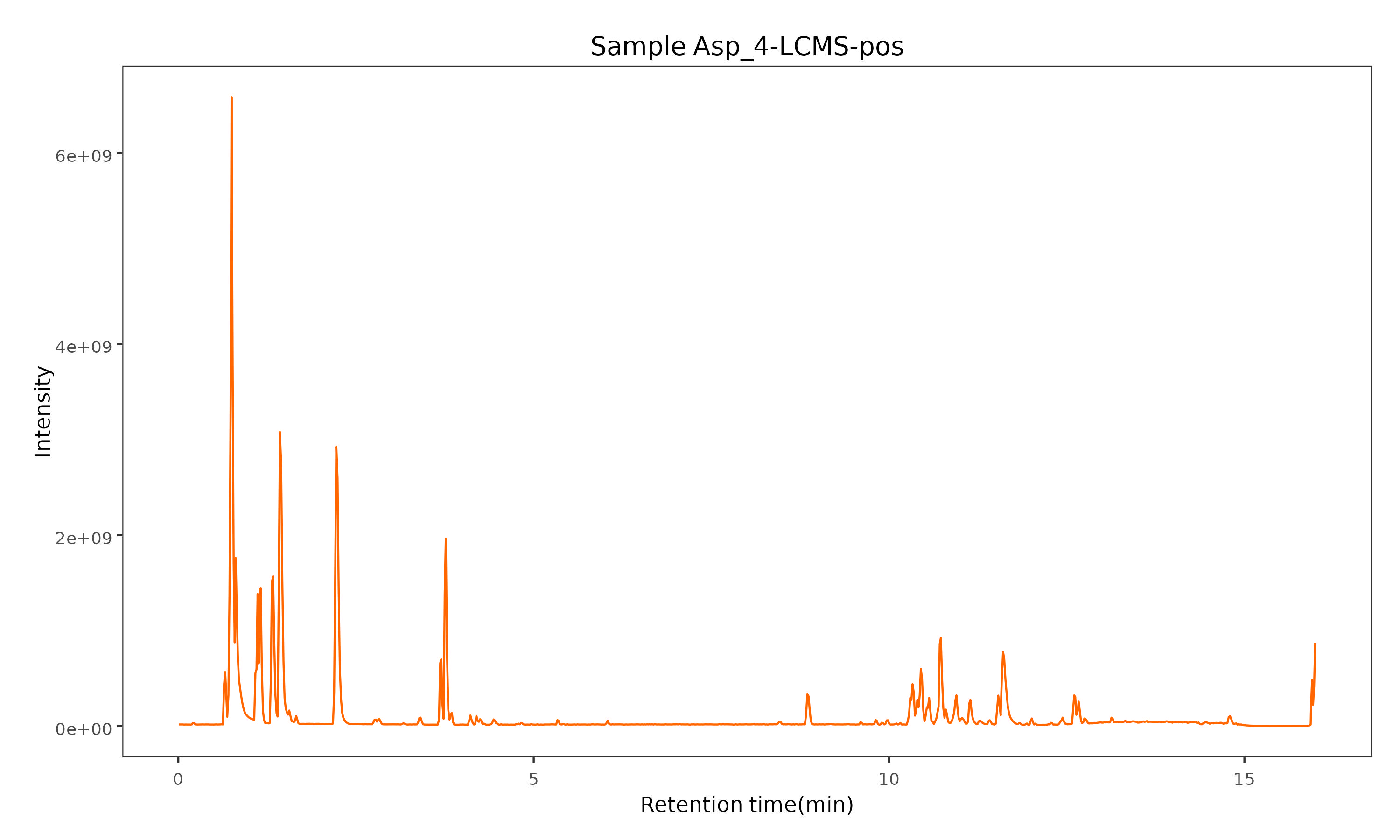

Supplement: Supplementary material S1 — The main instruments used during the LC-MS process, along with their models/specifications and manufacturers. [file Supplementary_file_1.zip › Metabolomics sequencing data FC1.2/1.基峰图/Asp_4-LCMS-pos-BPC.jpg]

Sample Asp\_4-LCMS-pos

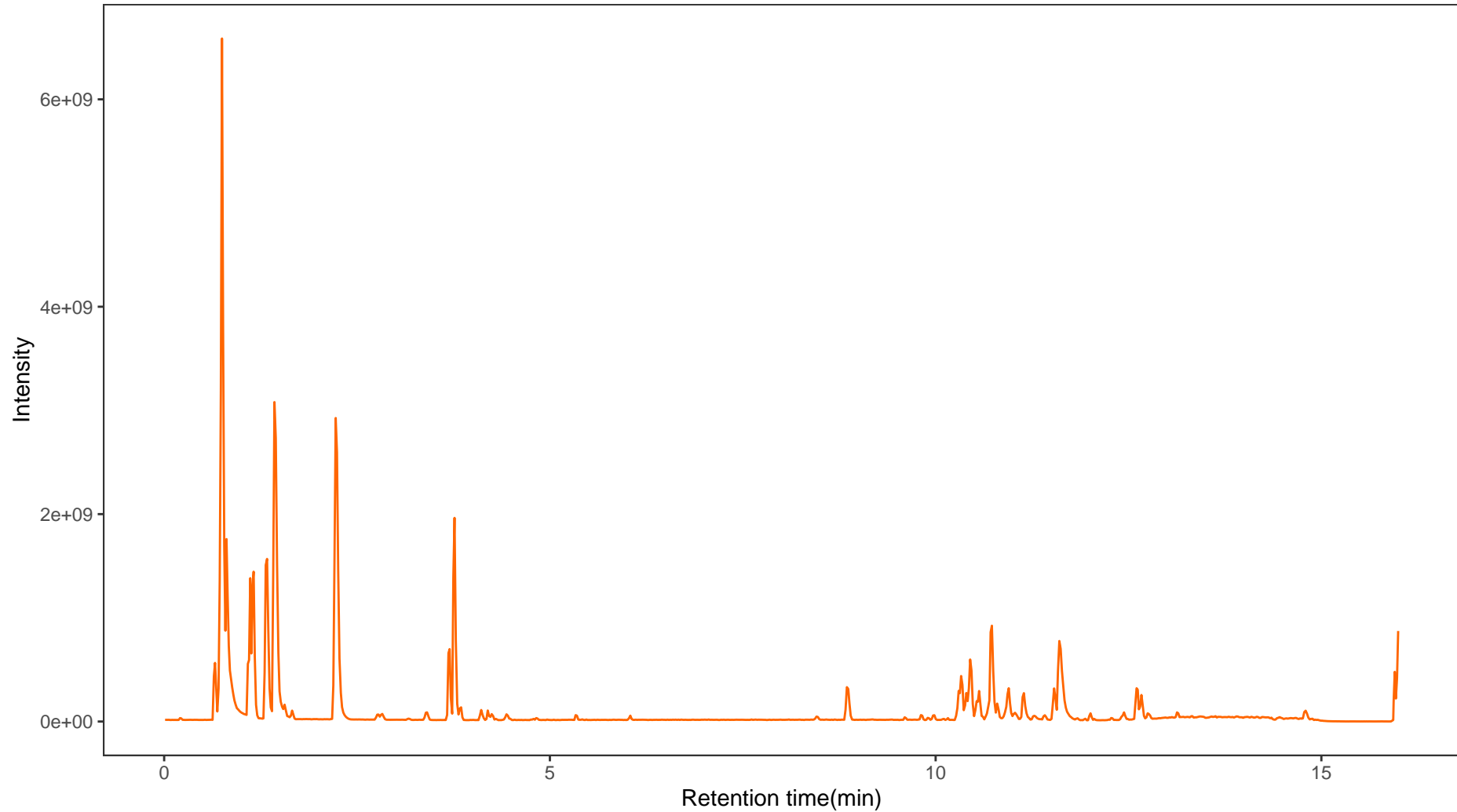

Supplement: Supplementary material S1 — The main instruments used during the LC-MS process, along with their models/specifications and manufacturers. [file Supplementary_file_1.zip › Metabolomics sequencing data FC1.2/1.基峰图/Asp_4-LCMS-pos-BPC.pdf]

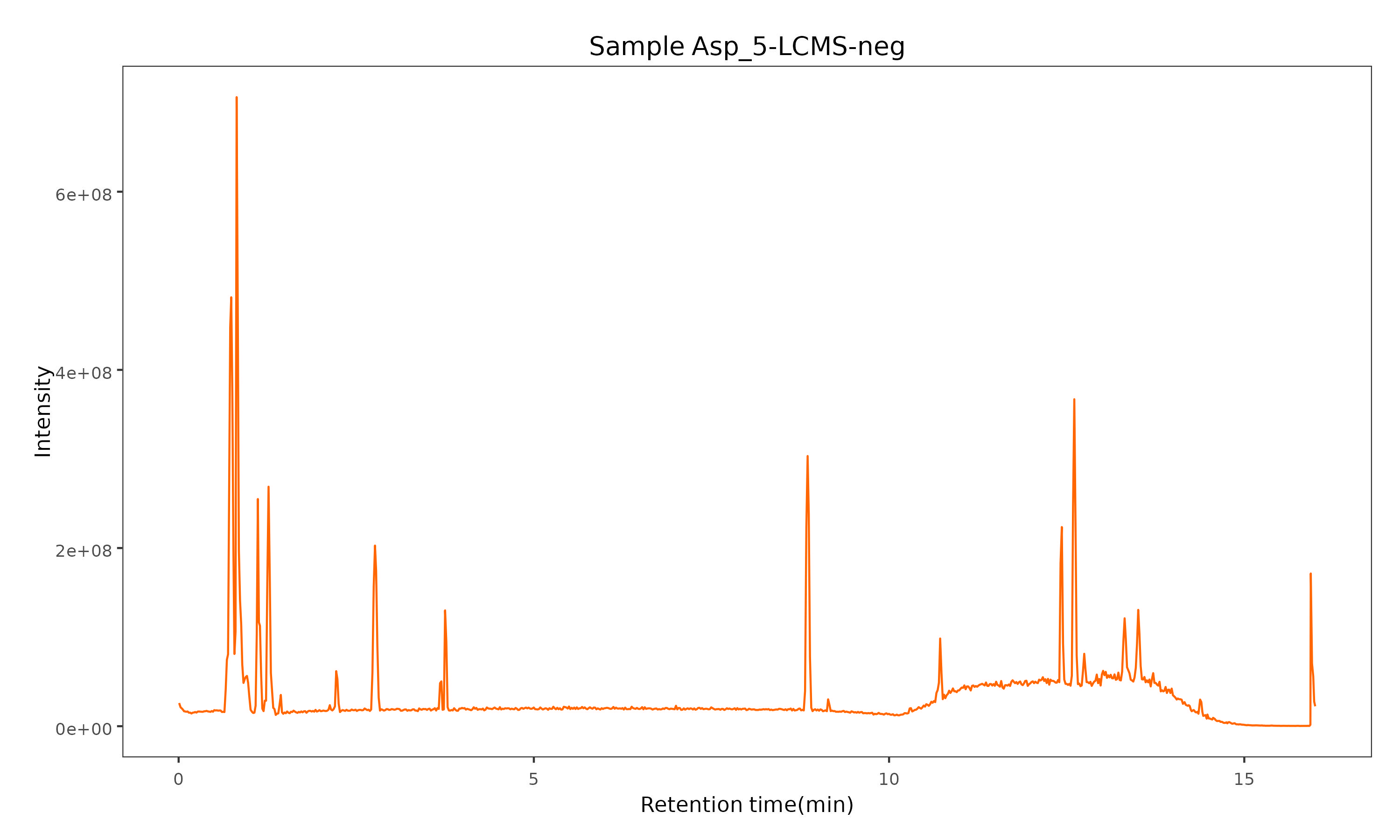

Supplement: Supplementary material S1 — The main instruments used during the LC-MS process, along with their models/specifications and manufacturers. [file Supplementary_file_1.zip › Metabolomics sequencing data FC1.2/1.基峰图/Asp_5-LCMS-neg-BPC.jpg]

Sample Asp\_5-LCMS-neg

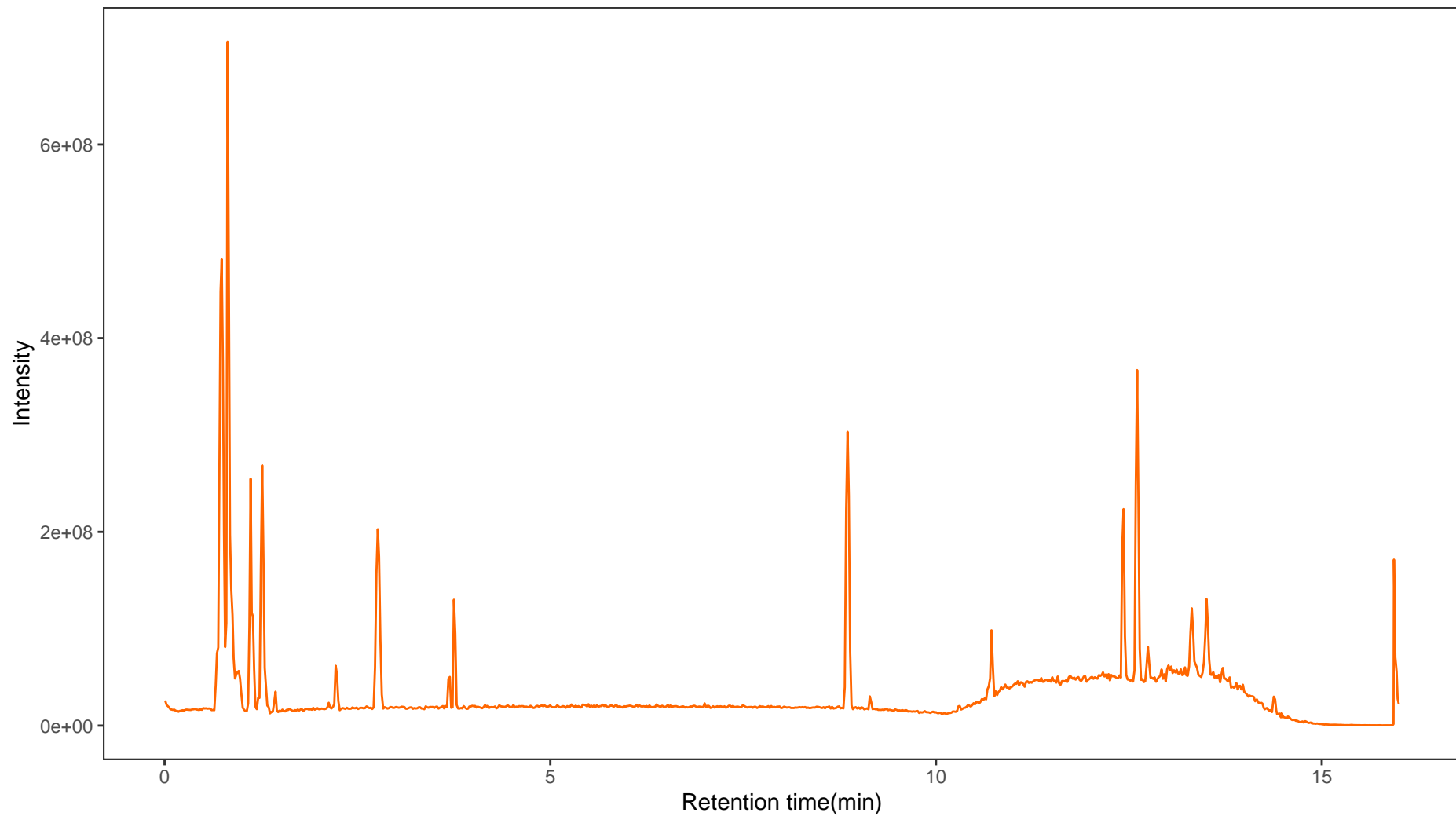

Supplement: Supplementary material S1 — The main instruments used during the LC-MS process, along with their models/specifications and manufacturers. [file Supplementary_file_1.zip › Metabolomics sequencing data FC1.2/1.基峰图/Asp_5-LCMS-neg-BPC.pdf]

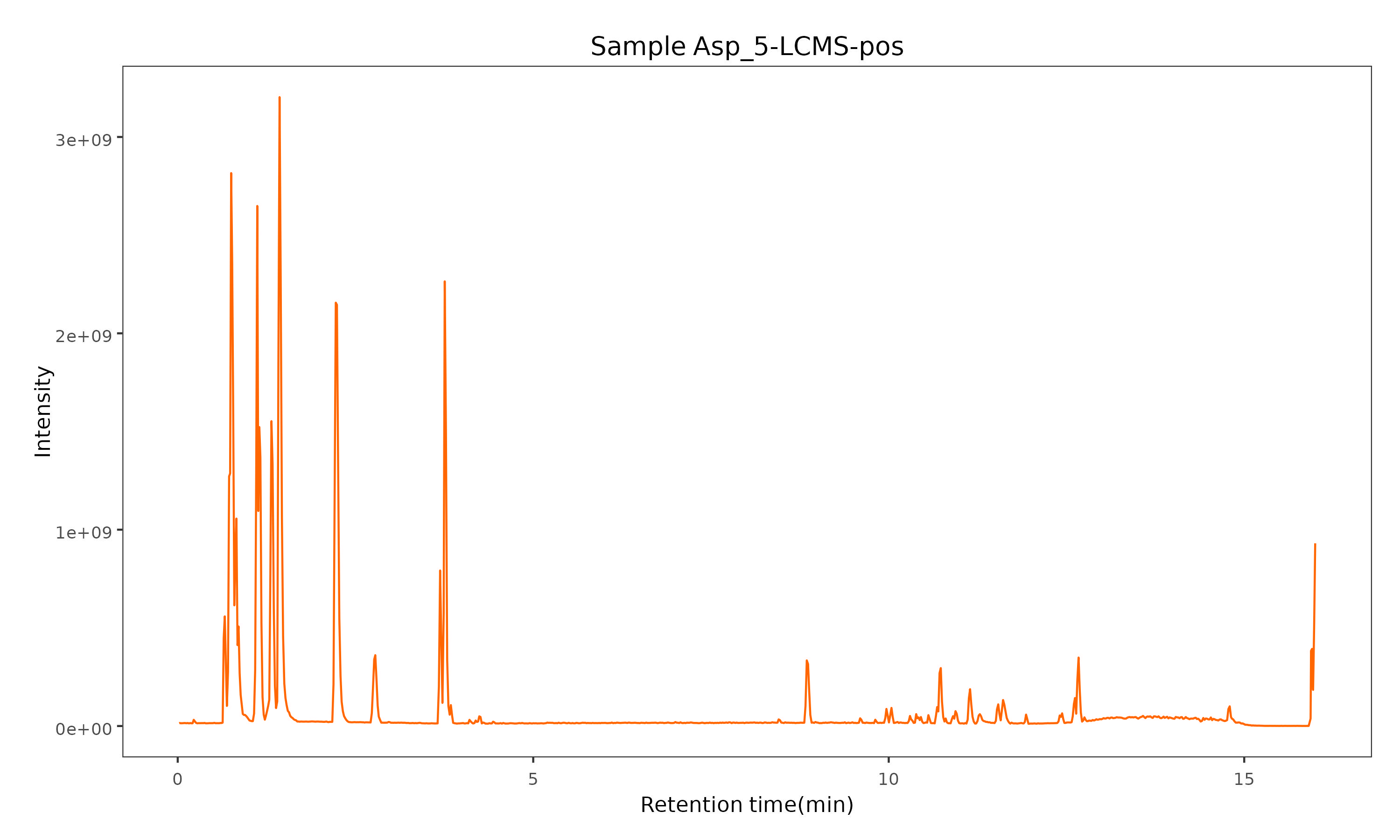

Supplement: Supplementary material S1 — The main instruments used during the LC-MS process, along with their models/specifications and manufacturers. [file Supplementary_file_1.zip › Metabolomics sequencing data FC1.2/1.基峰图/Asp_5-LCMS-pos-BPC.jpg]

Sample Asp\_5-LCMS-pos

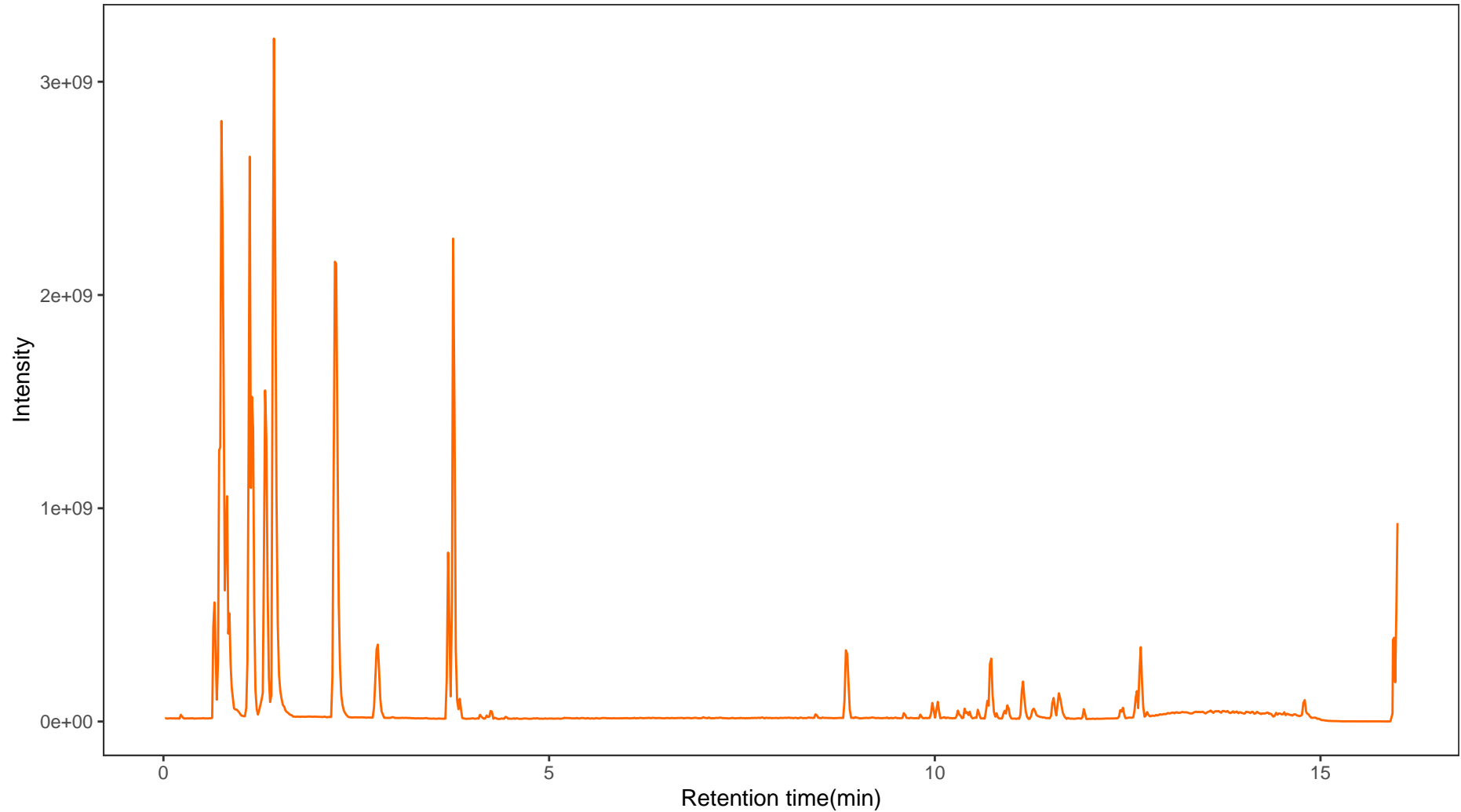

Supplement: Supplementary material S1 — The main instruments used during the LC-MS process, along with their models/specifications and manufacturers. [file Supplementary_file_1.zip › Metabolomics sequencing data FC1.2/1.基峰图/Asp_5-LCMS-pos-BPC.pdf]

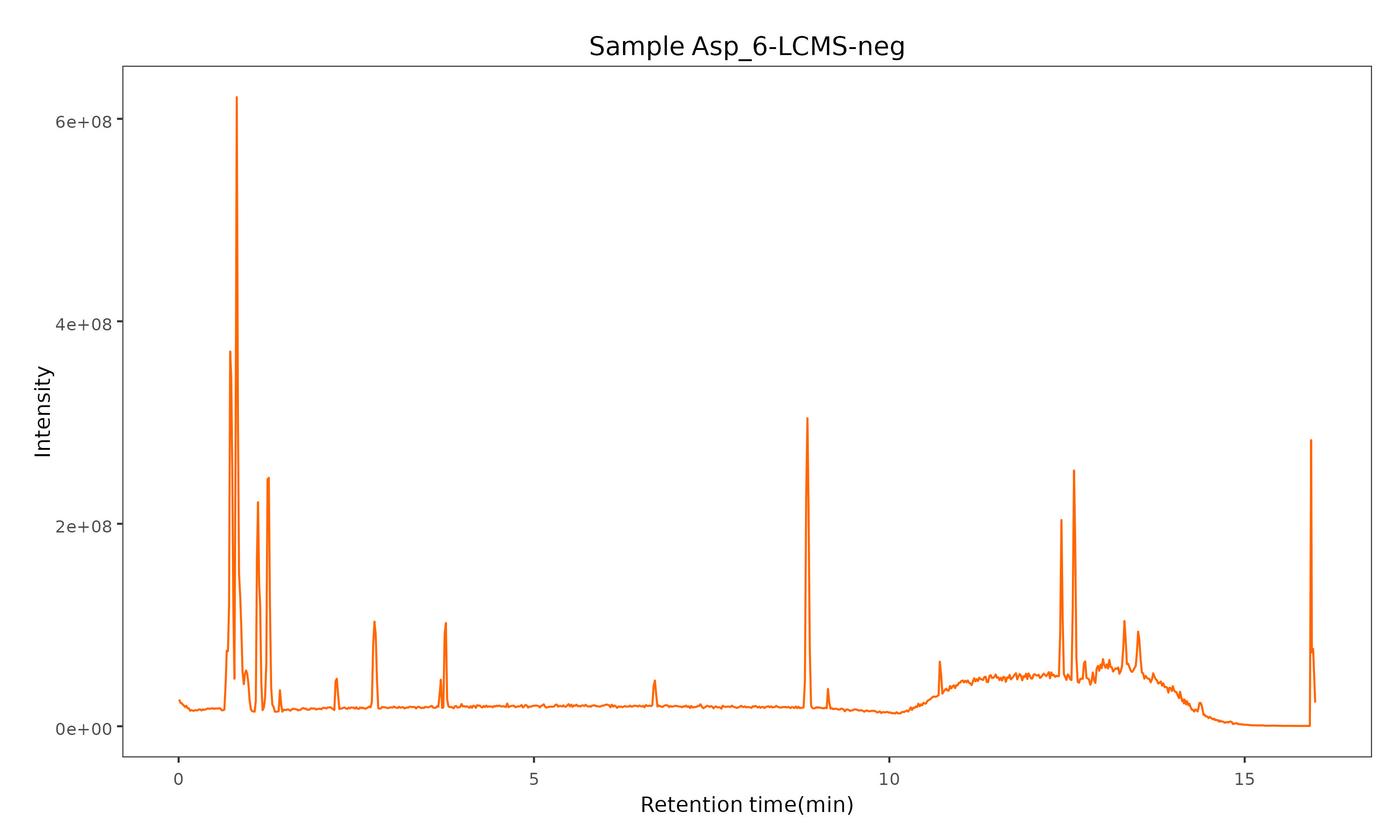

Supplement: Supplementary material S1 — The main instruments used during the LC-MS process, along with their models/specifications and manufacturers. [file Supplementary_file_1.zip › Metabolomics sequencing data FC1.2/1.基峰图/Asp_6-LCMS-neg-BPC.jpg]

Sample Asp\_6-LCMS-neg

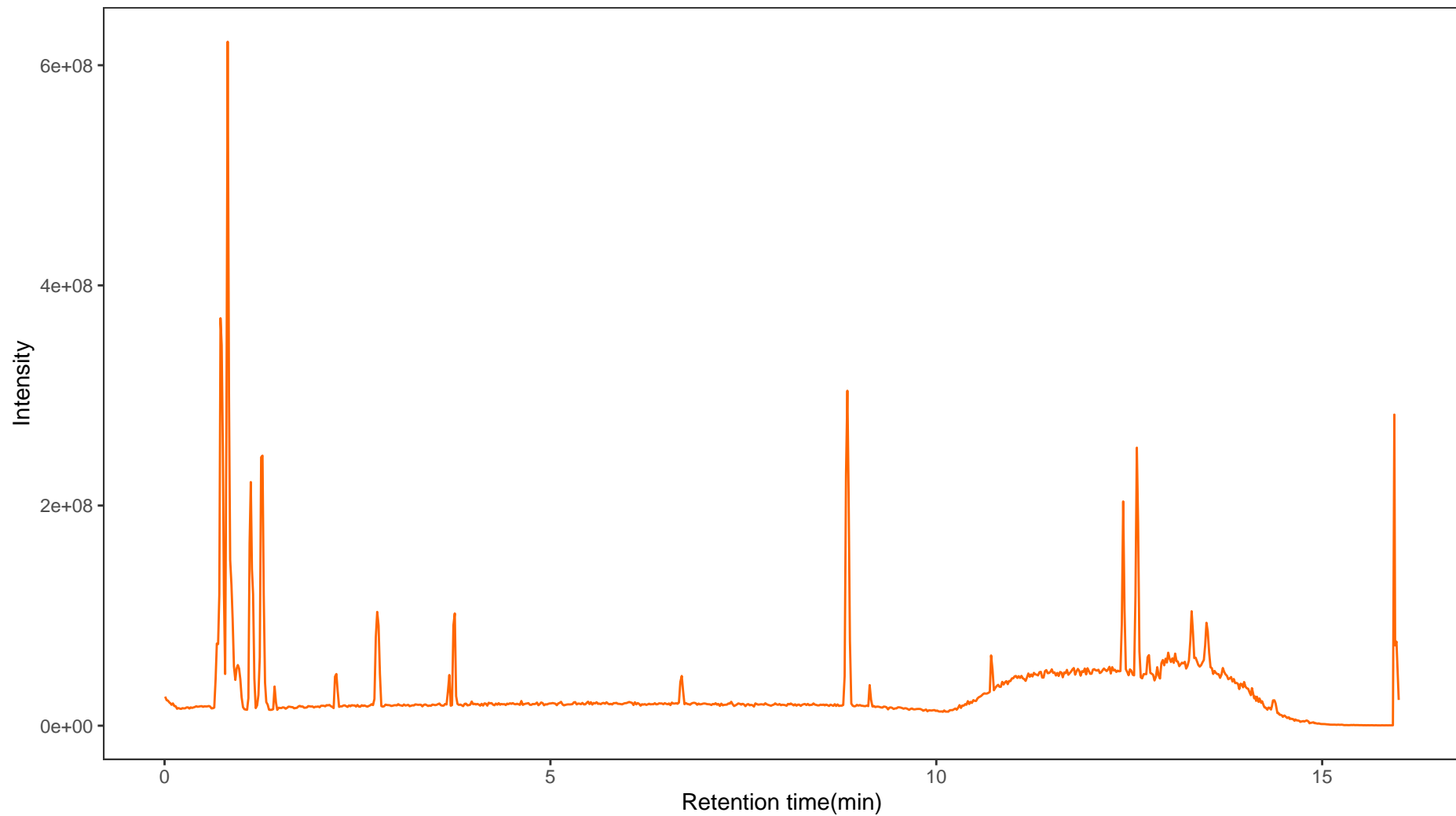

Supplement: Supplementary material S1 — The main instruments used during the LC-MS process, along with their models/specifications and manufacturers. [file Supplementary_file_1.zip › Metabolomics sequencing data FC1.2/1.基峰图/Asp_6-LCMS-neg-BPC.pdf]

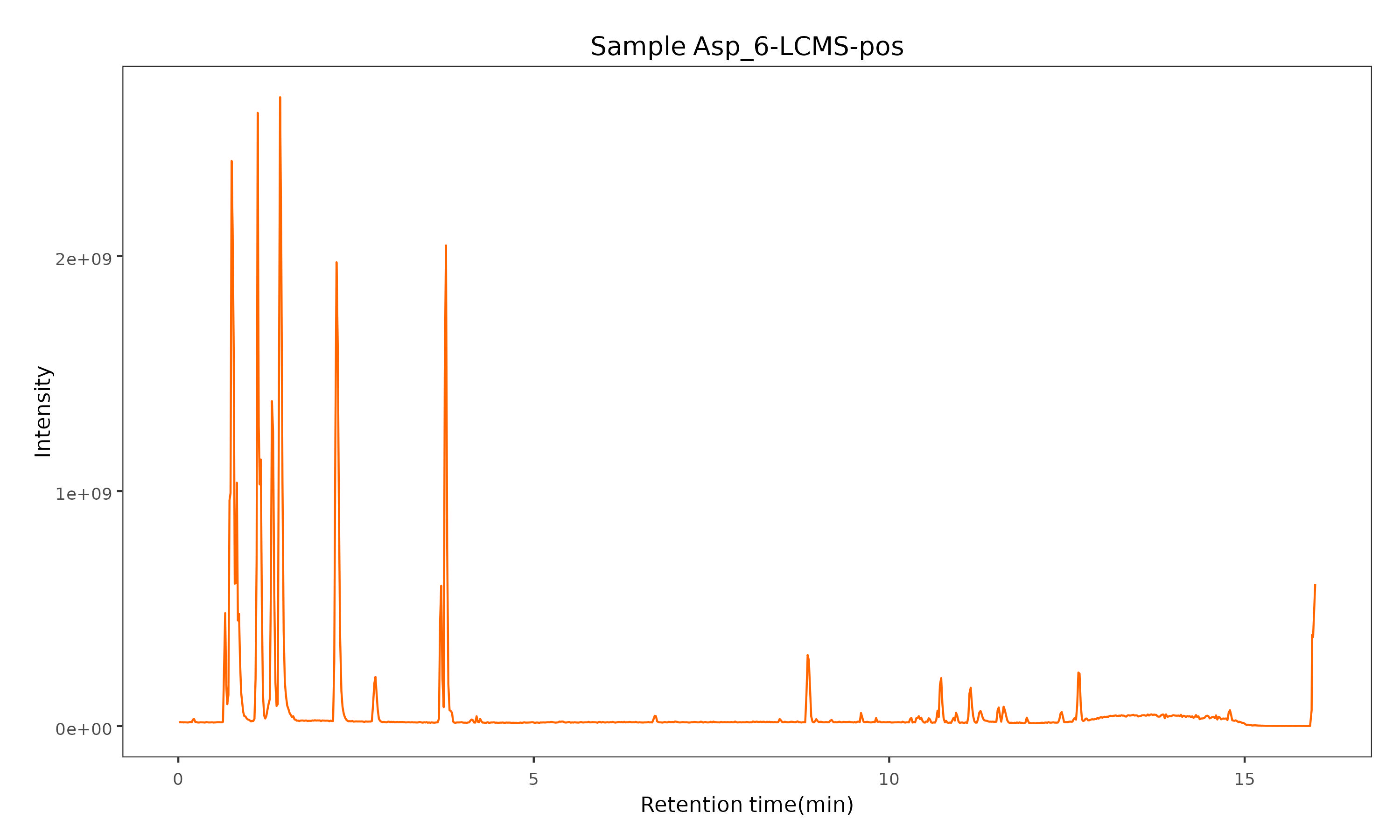

Supplement: Supplementary material S1 — The main instruments used during the LC-MS process, along with their models/specifications and manufacturers. [file Supplementary_file_1.zip › Metabolomics sequencing data FC1.2/1.基峰图/Asp_6-LCMS-pos-BPC.jpg]

Sample Asp\_6-LCMS-pos

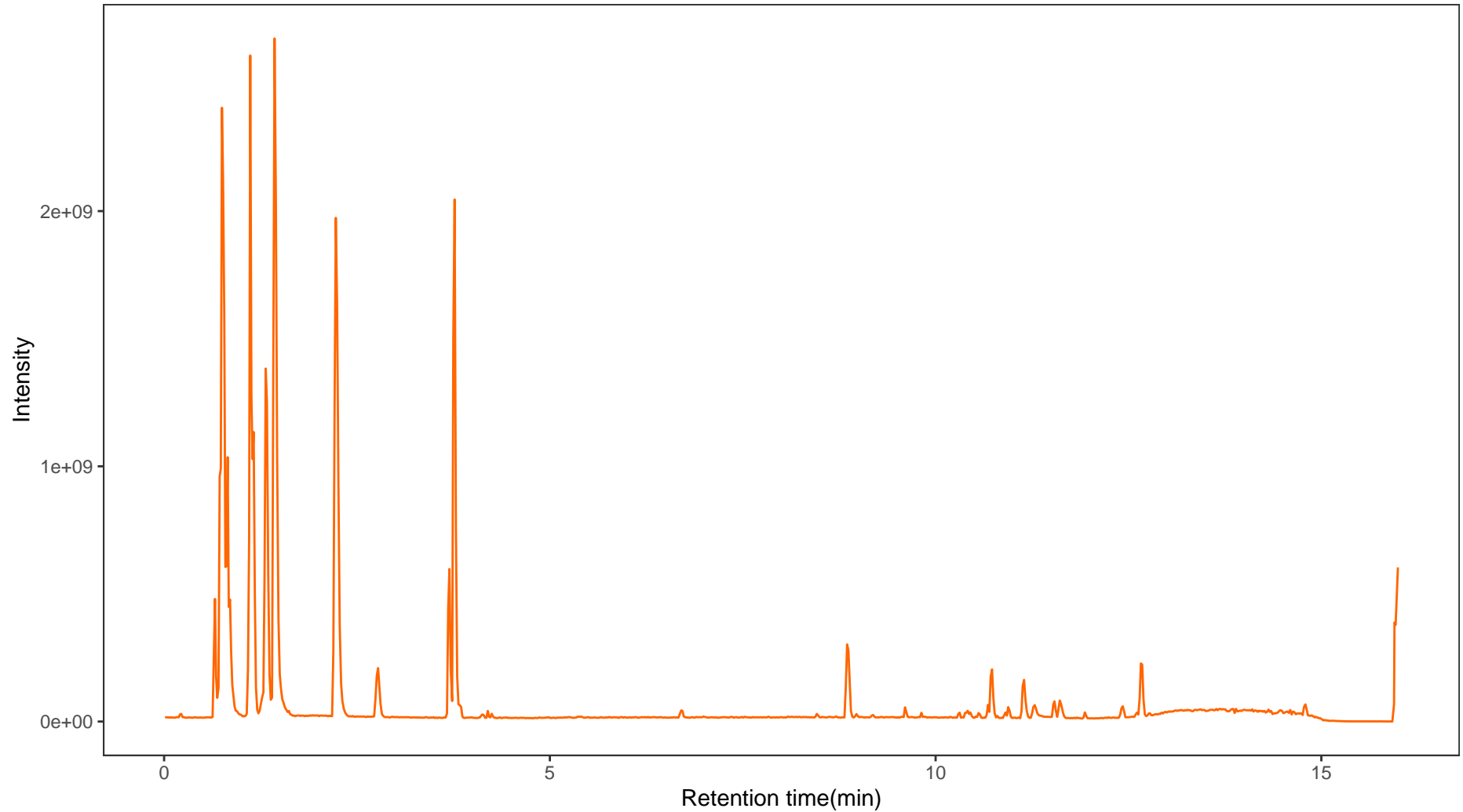

Supplement: Supplementary material S1 — The main instruments used during the LC-MS process, along with their models/specifications and manufacturers. [file Supplementary_file_1.zip › Metabolomics sequencing data FC1.2/1.基峰图/Asp_6-LCMS-pos-BPC.pdf]

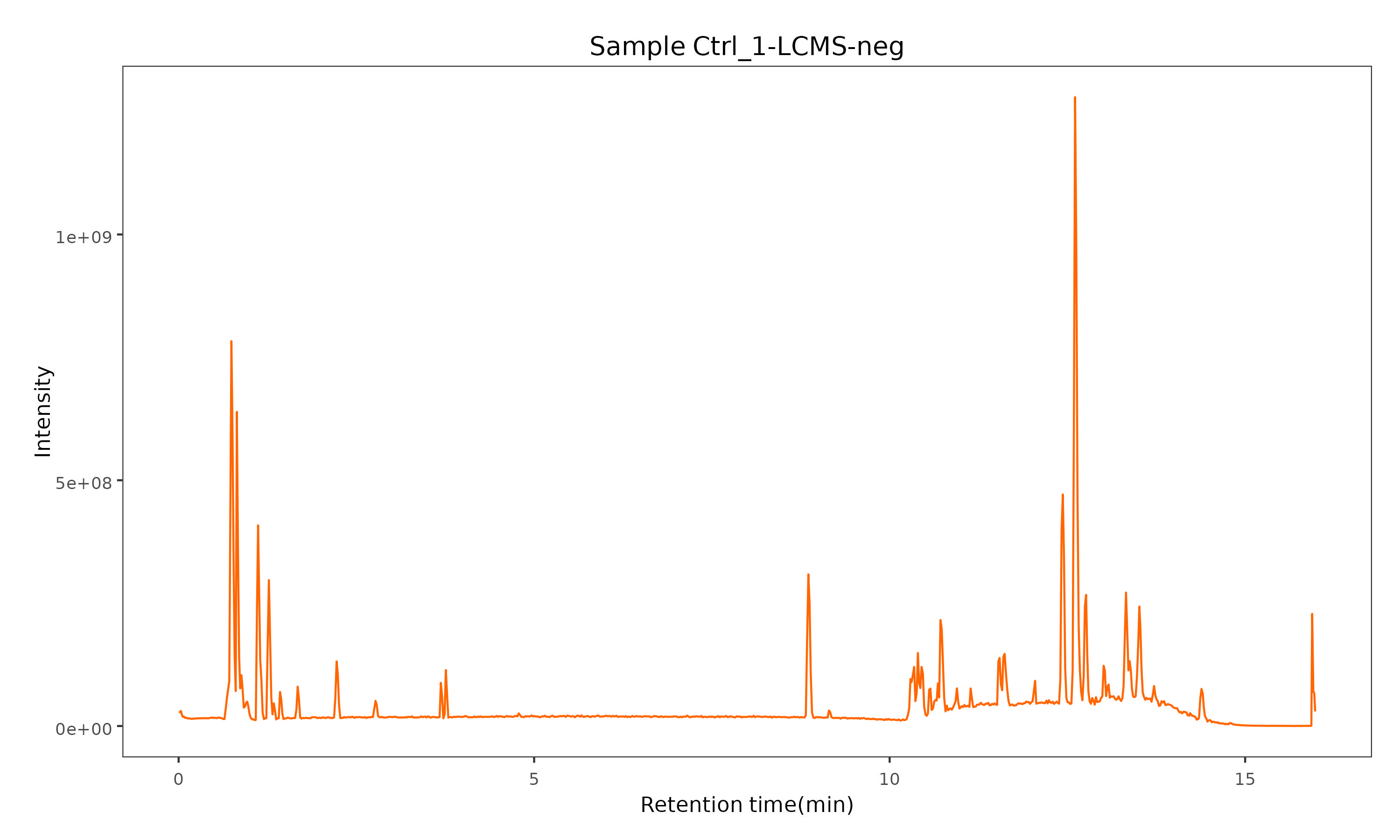

Supplement: Supplementary material S1 — The main instruments used during the LC-MS process, along with their models/specifications and manufacturers. [file Supplementary_file_1.zip › Metabolomics sequencing data FC1.2/1.基峰图/Ctrl_1-LCMS-neg-BPC.jpg]

Sample Ctrl\_1-LCMS-neg

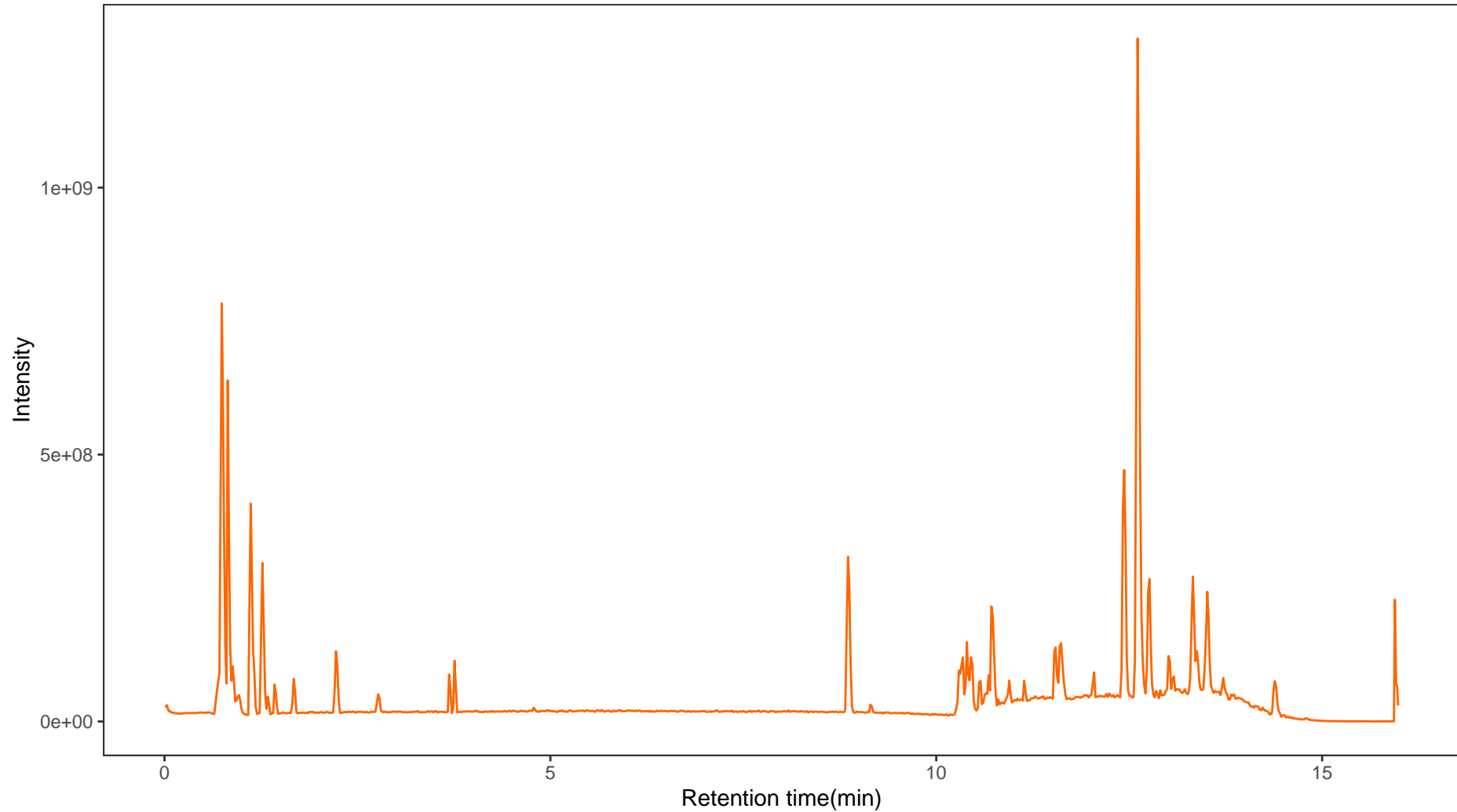

Supplement: Supplementary material S1 — The main instruments used during the LC-MS process, along with their models/specifications and manufacturers. [file Supplementary_file_1.zip › Metabolomics sequencing data FC1.2/1.基峰图/Ctrl_1-LCMS-neg-BPC.pdf]

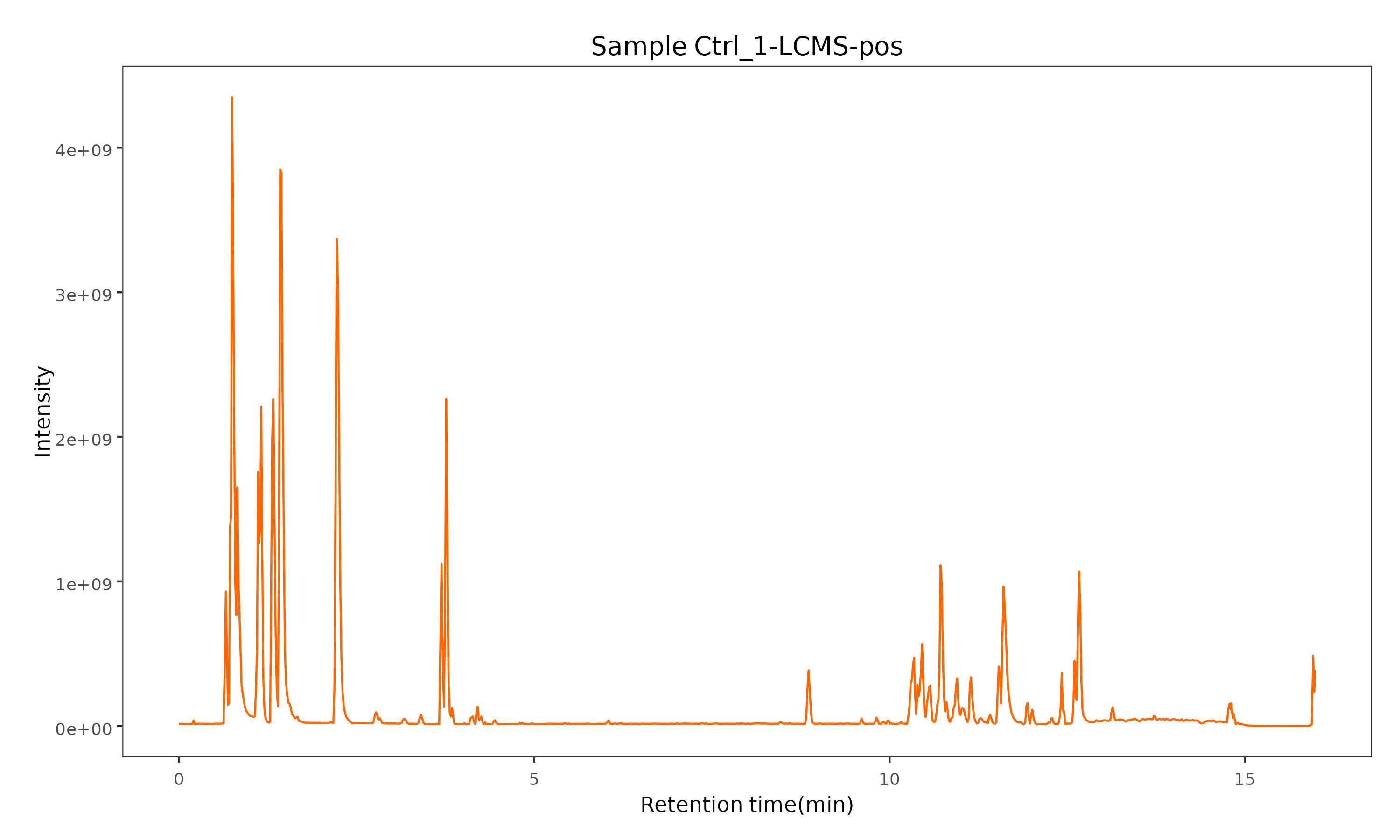

Supplement: Supplementary material S1 — The main instruments used during the LC-MS process, along with their models/specifications and manufacturers. [file Supplementary_file_1.zip › Metabolomics sequencing data FC1.2/1.基峰图/Ctrl_1-LCMS-pos-BPC.jpg]

Sample Ctrl\_1-LCMS-pos

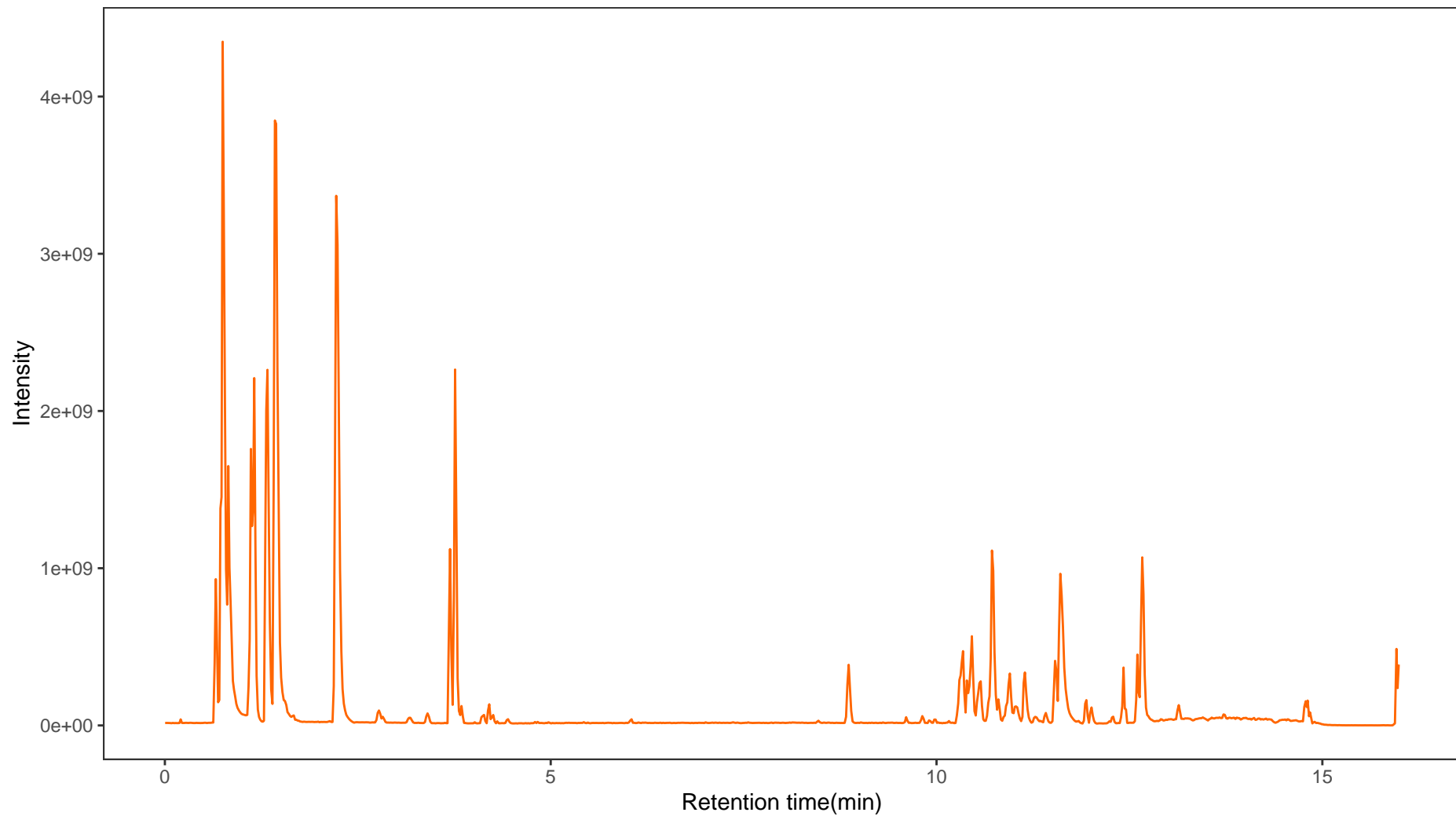

Supplement: Supplementary material S1 — The main instruments used during the LC-MS process, along with their models/specifications and manufacturers. [file Supplementary_file_1.zip › Metabolomics sequencing data FC1.2/1.基峰图/Ctrl_1-LCMS-pos-BPC.pdf]

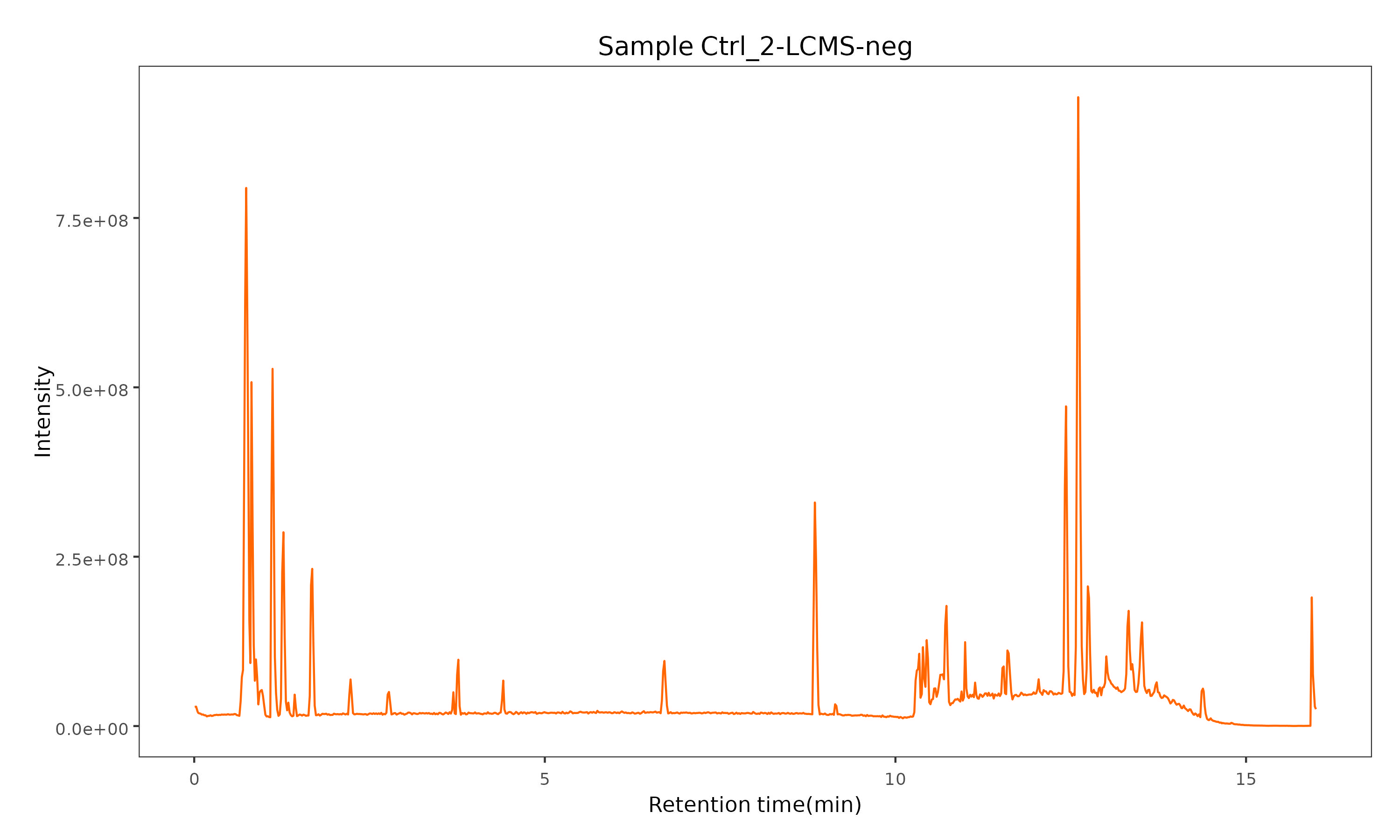

Supplement: Supplementary material S1 — The main instruments used during the LC-MS process, along with their models/specifications and manufacturers. [file Supplementary_file_1.zip › Metabolomics sequencing data FC1.2/1.基峰图/Ctrl_2-LCMS-neg-BPC.jpg]

Sample Ctrl\_2-LCMS-neg

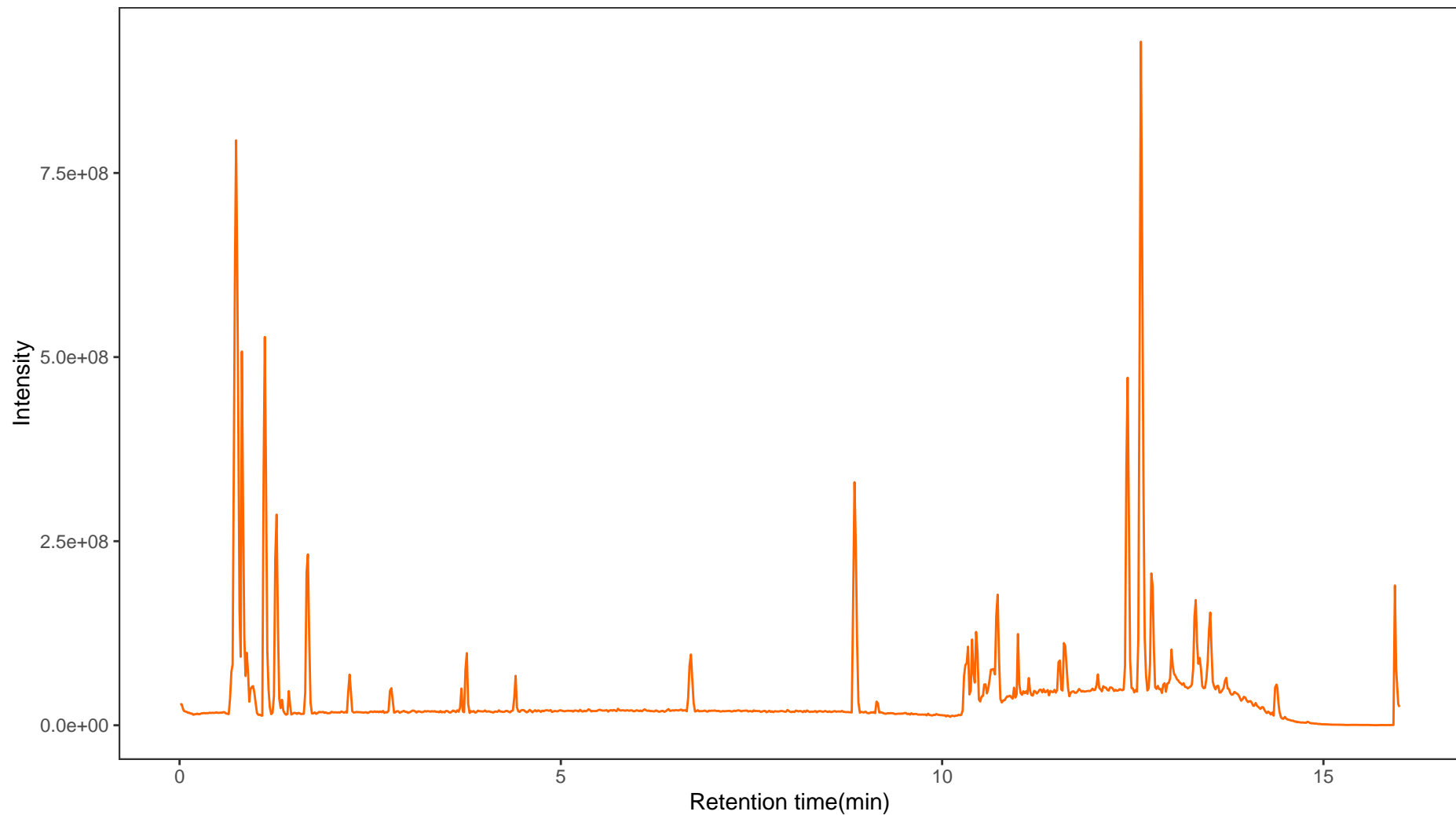

Supplement: Supplementary material S1 — The main instruments used during the LC-MS process, along with their models/specifications and manufacturers. [file Supplementary_file_1.zip › Metabolomics sequencing data FC1.2/1.基峰图/Ctrl_2-LCMS-neg-BPC.pdf]

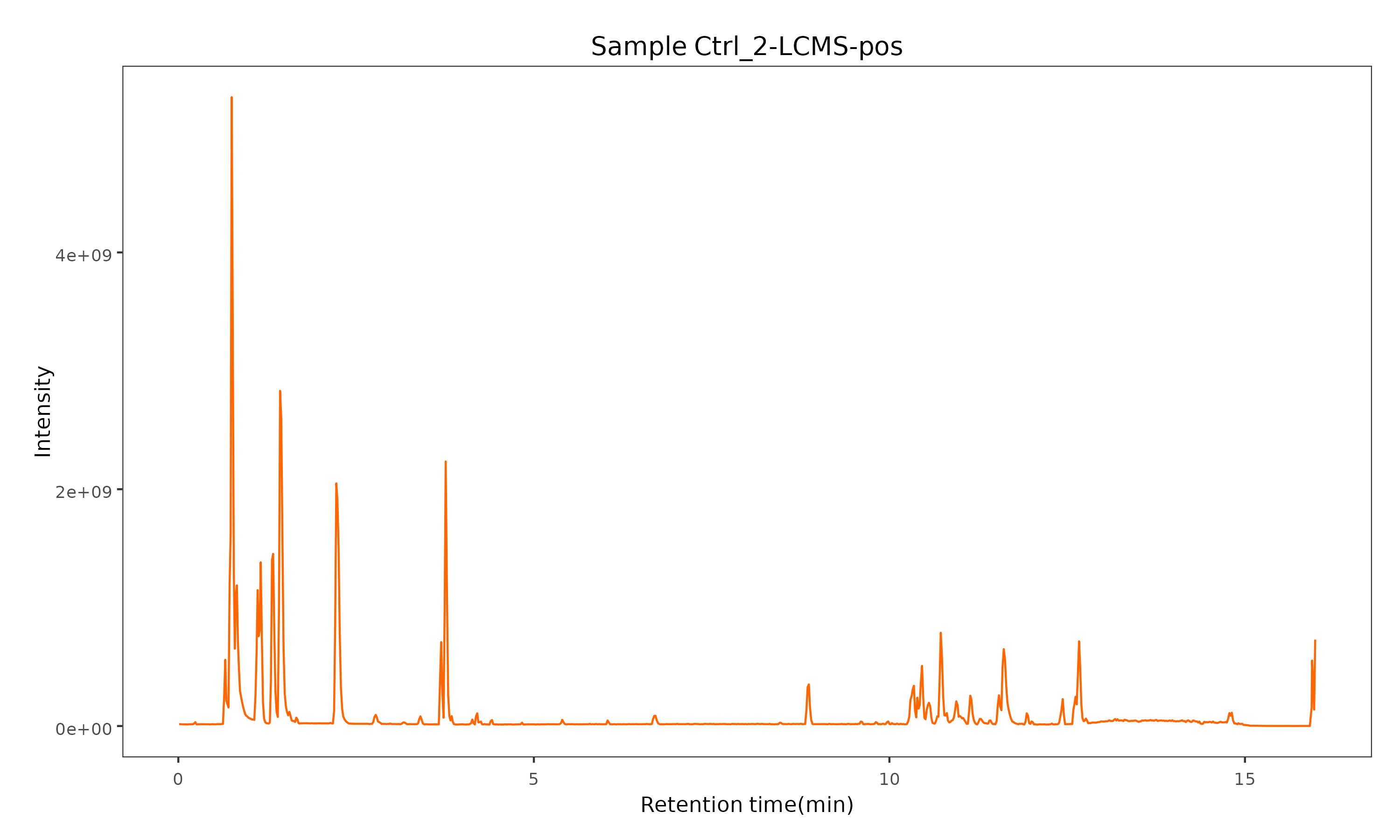

Supplement: Supplementary material S1 — The main instruments used during the LC-MS process, along with their models/specifications and manufacturers. [file Supplementary_file_1.zip › Metabolomics sequencing data FC1.2/1.基峰图/Ctrl_2-LCMS-pos-BPC.jpg]

Sample Ctrl\_2-LCMS-pos

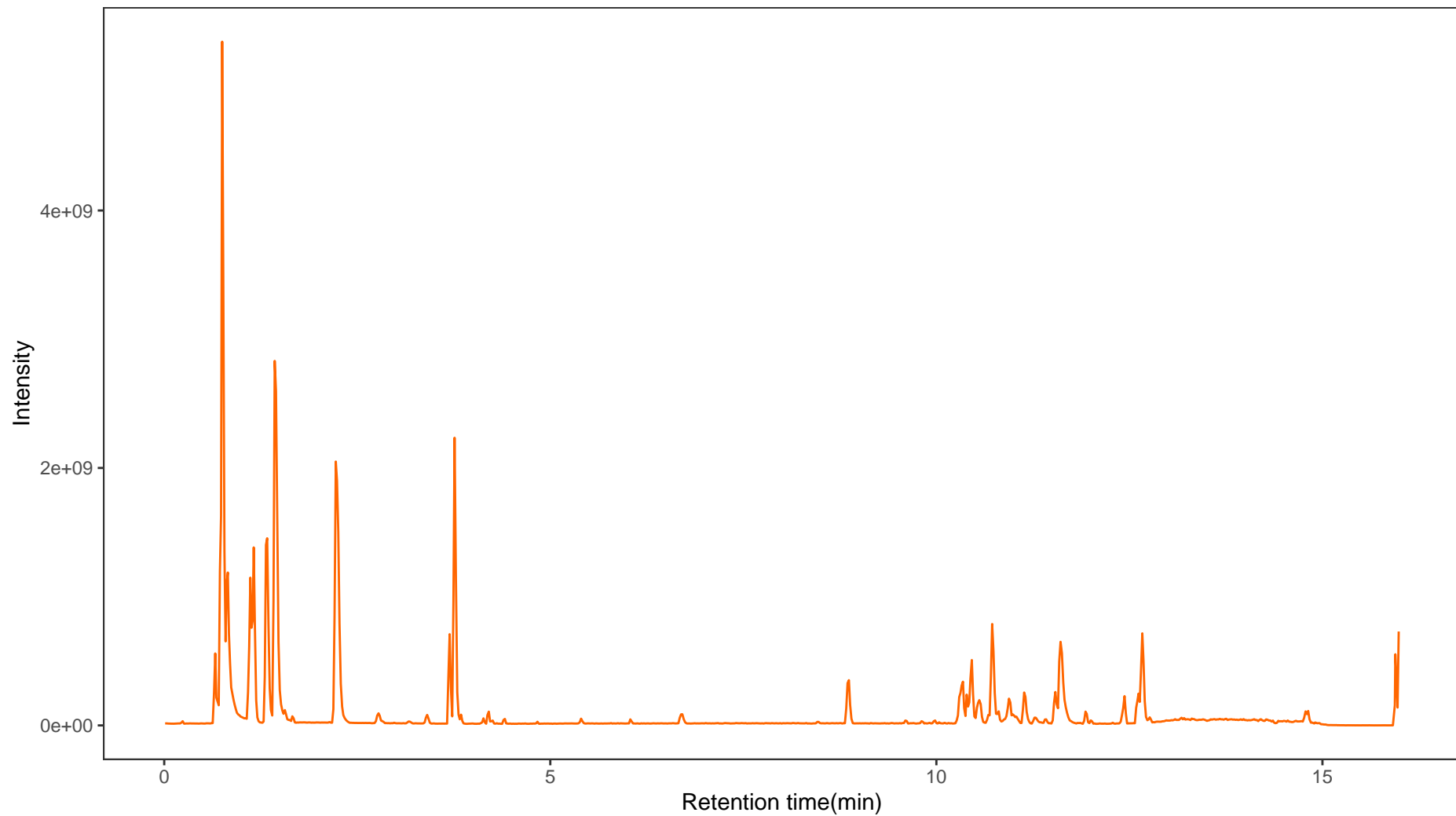

Supplement: Supplementary material S1 — The main instruments used during the LC-MS process, along with their models/specifications and manufacturers. [file Supplementary_file_1.zip › Metabolomics sequencing data FC1.2/1.基峰图/Ctrl_2-LCMS-pos-BPC.pdf]

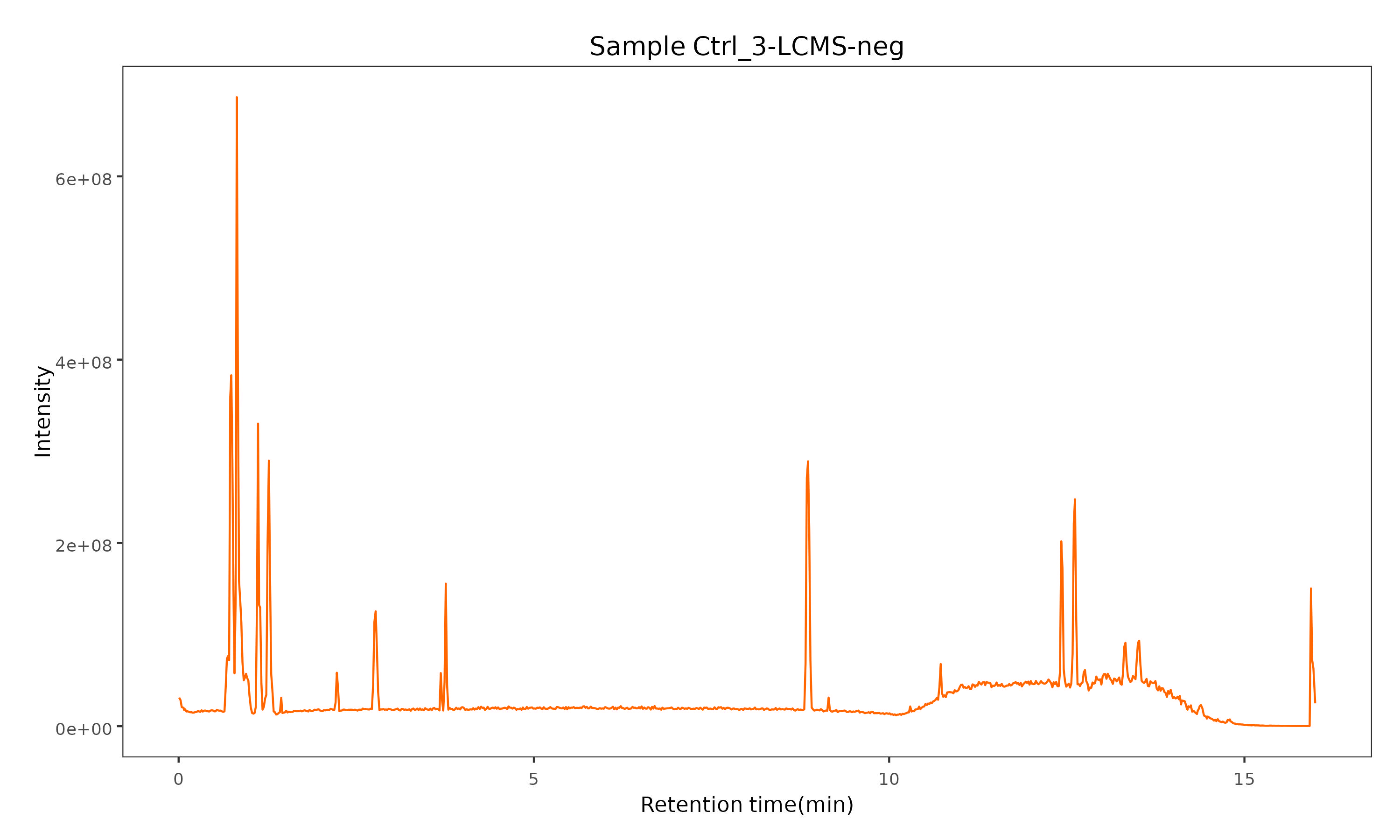

Supplement: Supplementary material S1 — The main instruments used during the LC-MS process, along with their models/specifications and manufacturers. [file Supplementary_file_1.zip › Metabolomics sequencing data FC1.2/1.基峰图/Ctrl_3-LCMS-neg-BPC.jpg]

Sample Ctrl\_3-LCMS-neg

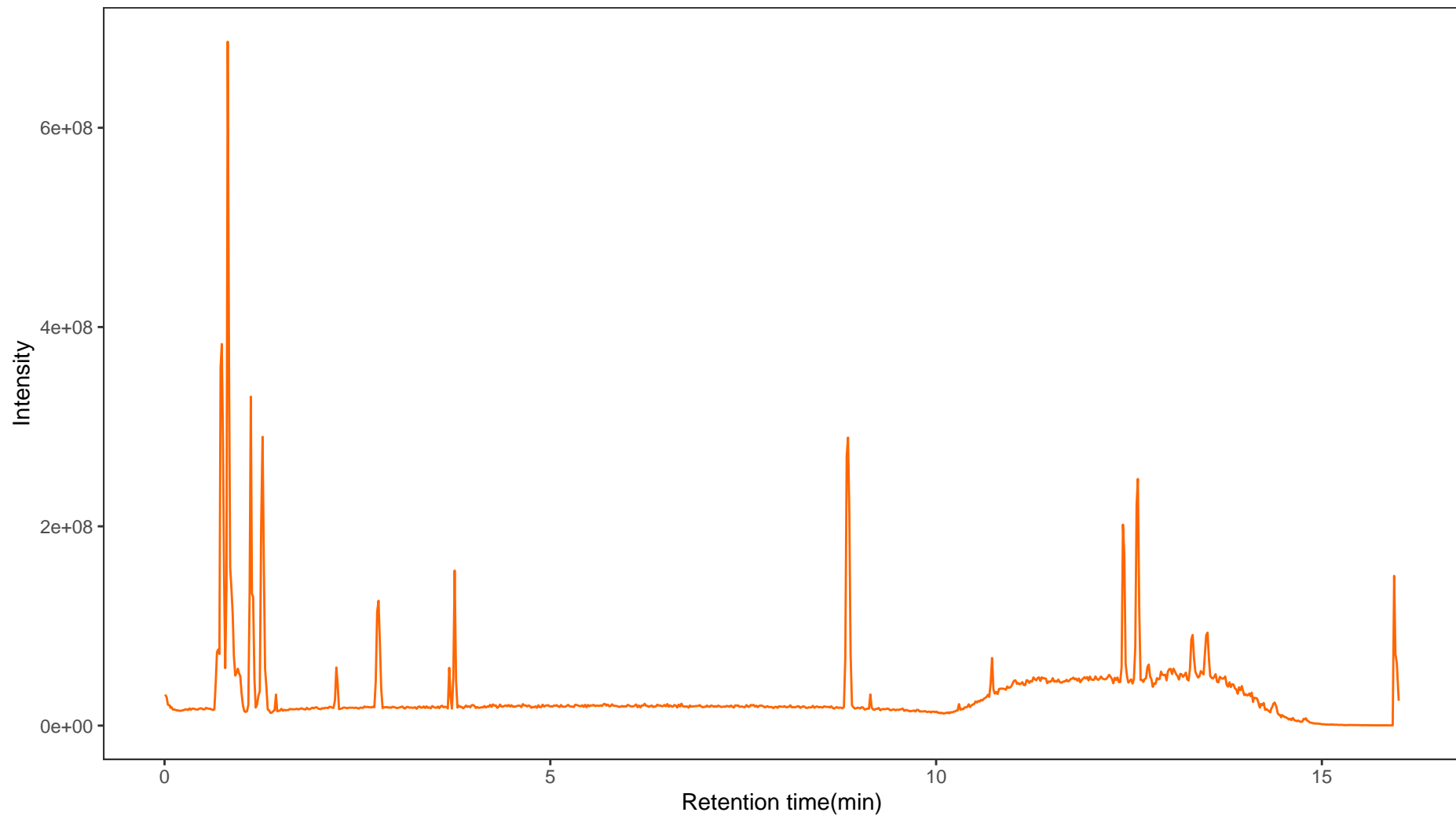

Supplement: Supplementary material S1 — The main instruments used during the LC-MS process, along with their models/specifications and manufacturers. [file Supplementary_file_1.zip › Metabolomics sequencing data FC1.2/1.基峰图/Ctrl_3-LCMS-neg-BPC.pdf]

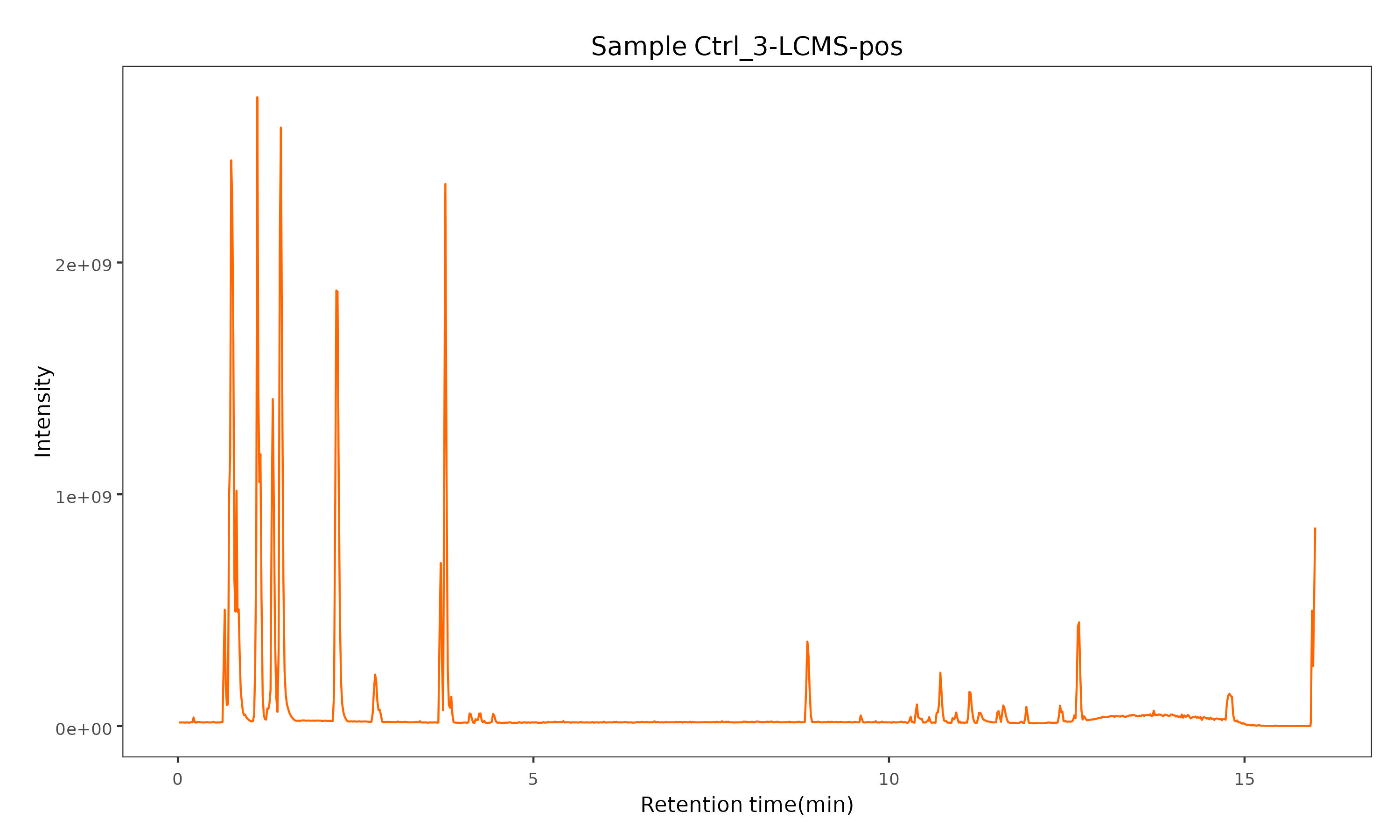

Supplement: Supplementary material S1 — The main instruments used during the LC-MS process, along with their models/specifications and manufacturers. [file Supplementary_file_1.zip › Metabolomics sequencing data FC1.2/1.基峰图/Ctrl_3-LCMS-pos-BPC.jpg]

Sample Ctrl\_3-LCMS-pos

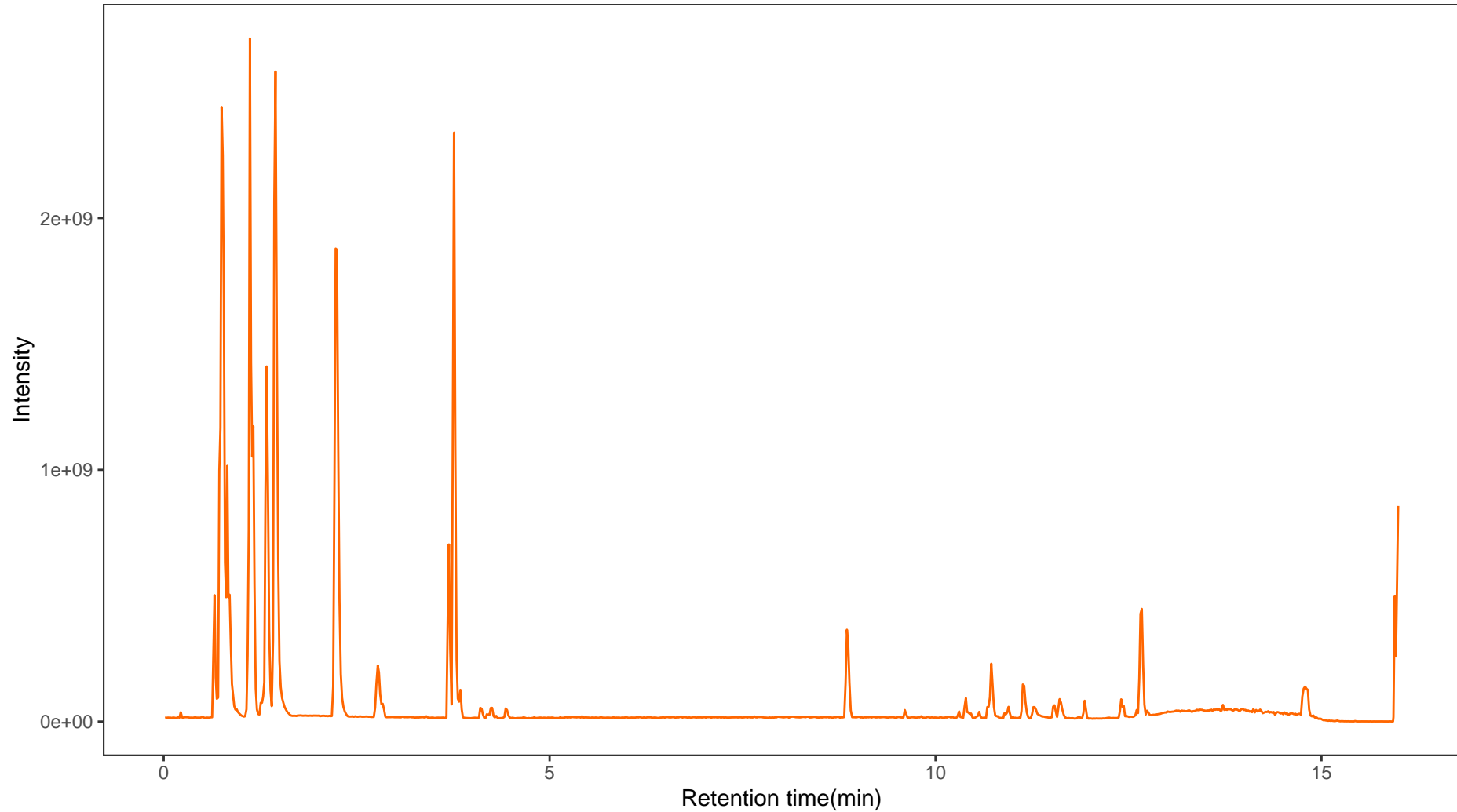

Supplement: Supplementary material S1 — The main instruments used during the LC-MS process, along with their models/specifications and manufacturers. [file Supplementary_file_1.zip › Metabolomics sequencing data FC1.2/1.基峰图/Ctrl_3-LCMS-pos-BPC.pdf]

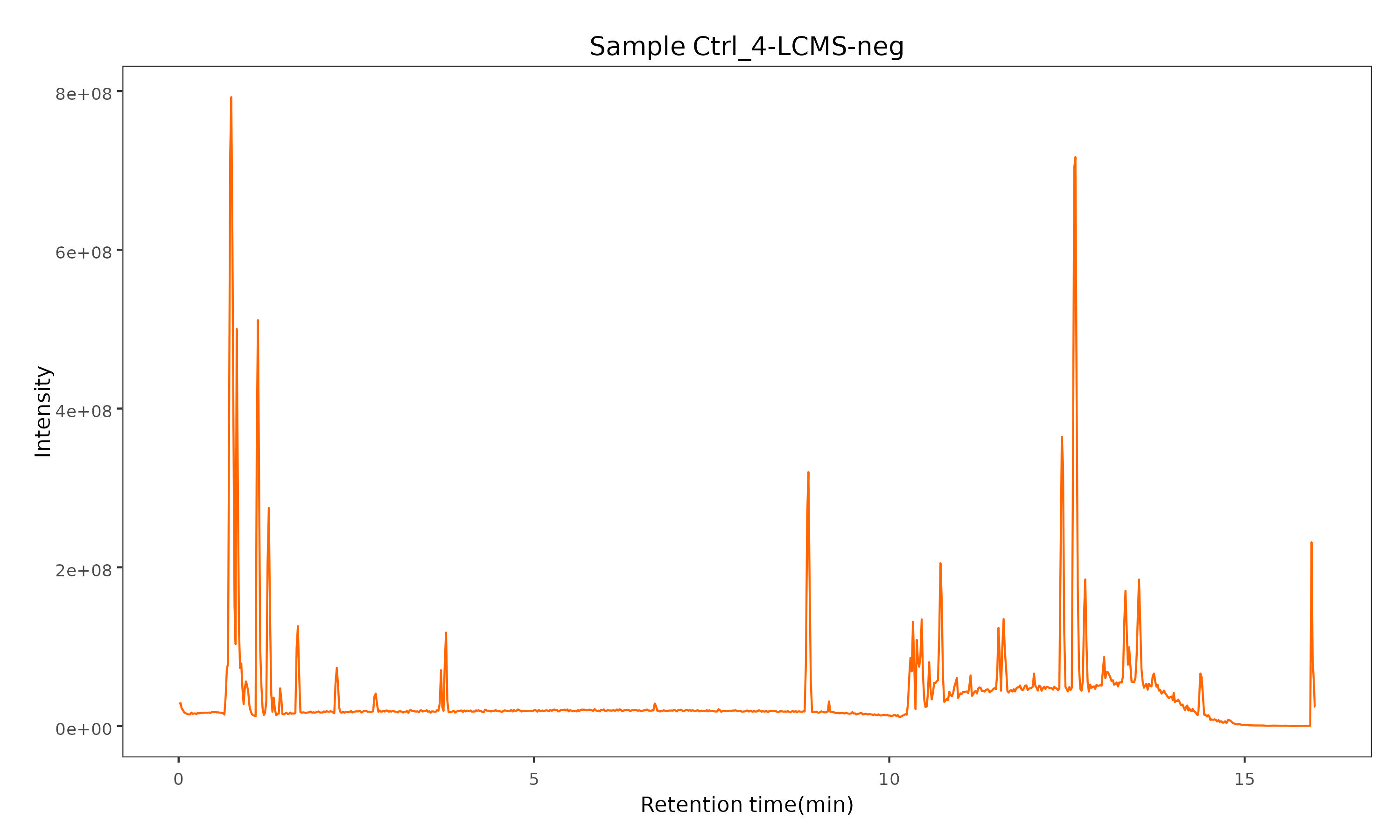

Supplement: Supplementary material S1 — The main instruments used during the LC-MS process, along with their models/specifications and manufacturers. [file Supplementary_file_1.zip › Metabolomics sequencing data FC1.2/1.基峰图/Ctrl_4-LCMS-neg-BPC.jpg]

Sample Ctrl\_4-LCMS-neg

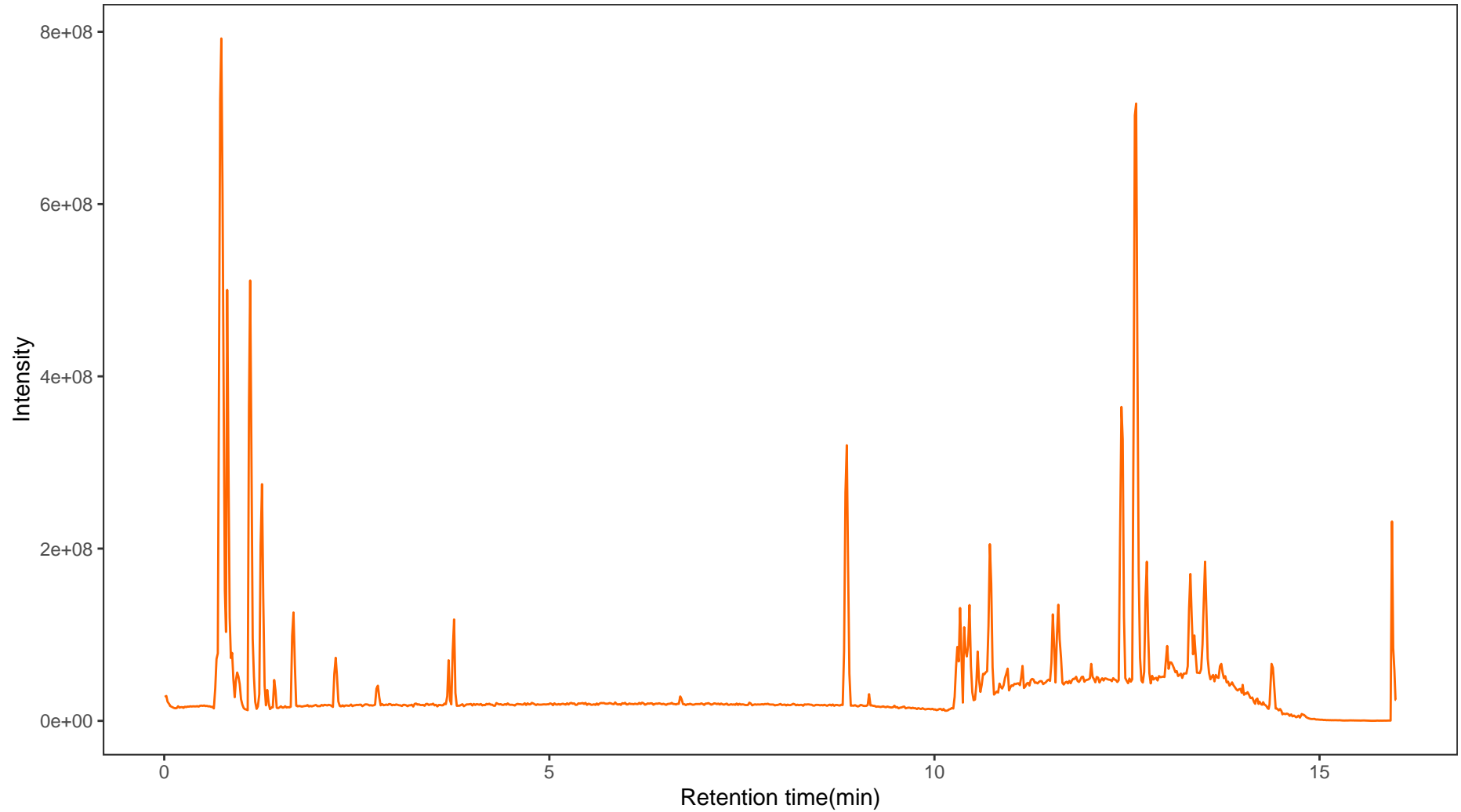

Supplement: Supplementary material S1 — The main instruments used during the LC-MS process, along with their models/specifications and manufacturers. [file Supplementary_file_1.zip › Metabolomics sequencing data FC1.2/1.基峰图/Ctrl_4-LCMS-neg-BPC.pdf]

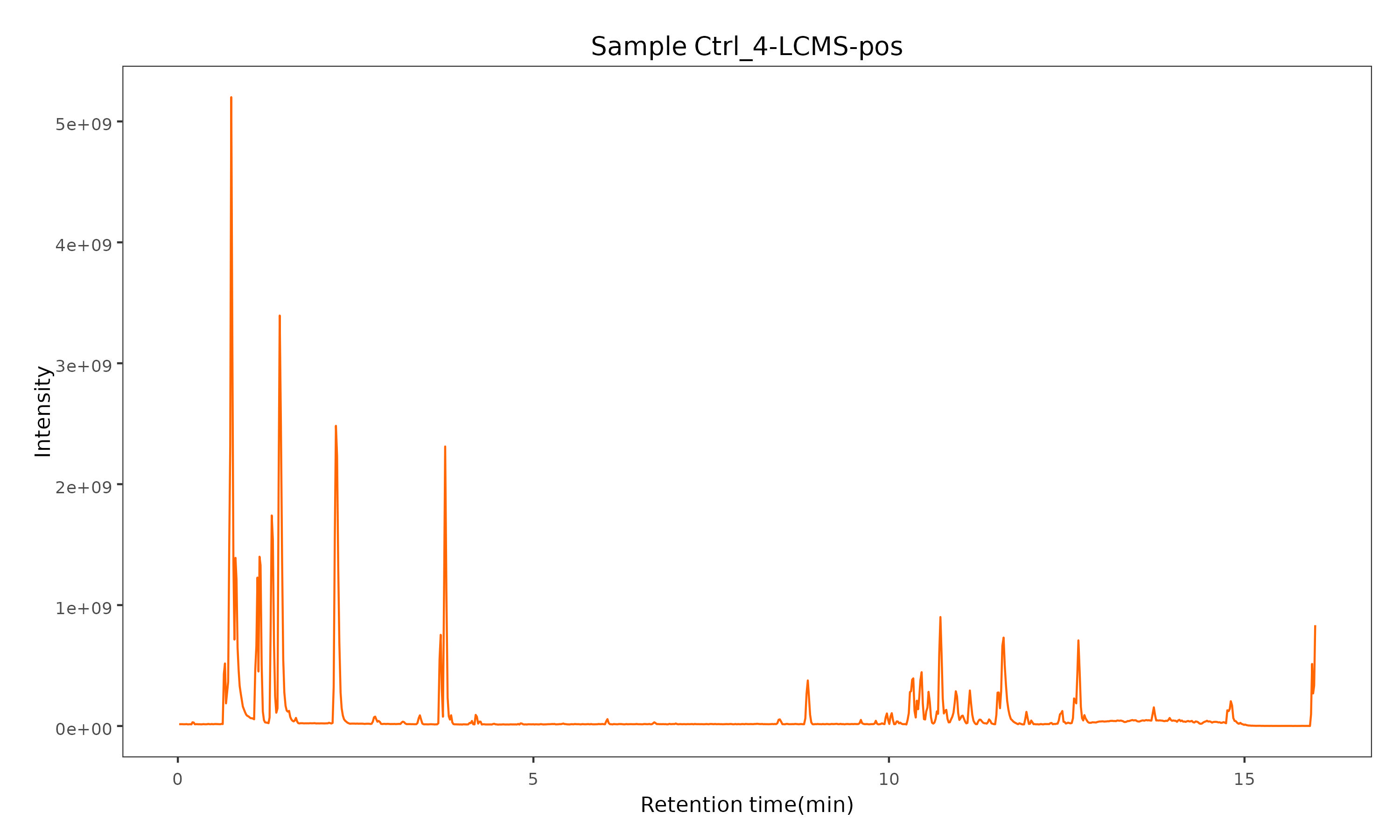

Supplement: Supplementary material S1 — The main instruments used during the LC-MS process, along with their models/specifications and manufacturers. [file Supplementary_file_1.zip › Metabolomics sequencing data FC1.2/1.基峰图/Ctrl_4-LCMS-pos-BPC.jpg]

Sample Ctrl\_4-LCMS-pos

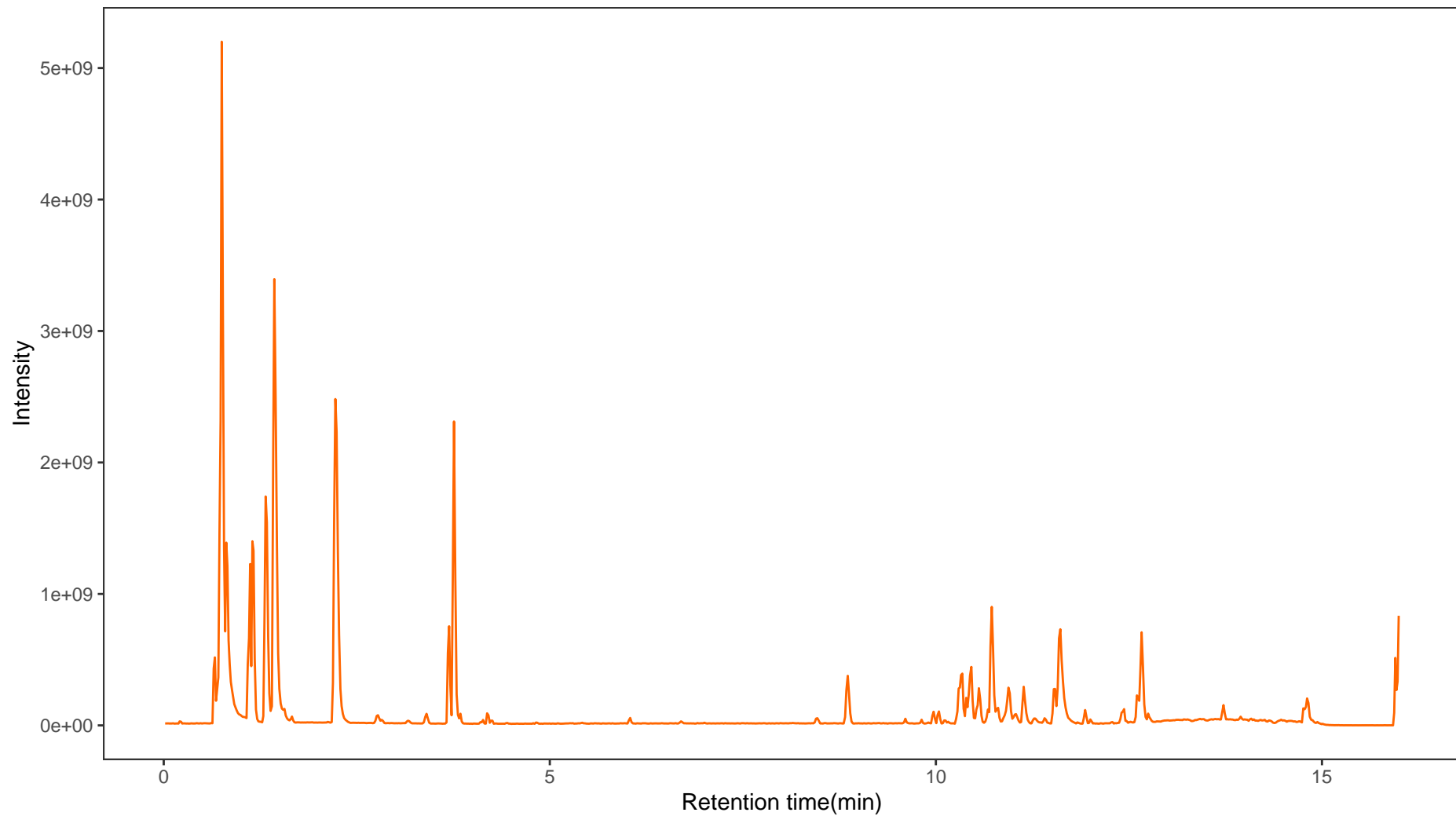

Supplement: Supplementary material S1 — The main instruments used during the LC-MS process, along with their models/specifications and manufacturers. [file Supplementary_file_1.zip › Metabolomics sequencing data FC1.2/1.基峰图/Ctrl_4-LCMS-pos-BPC.pdf]

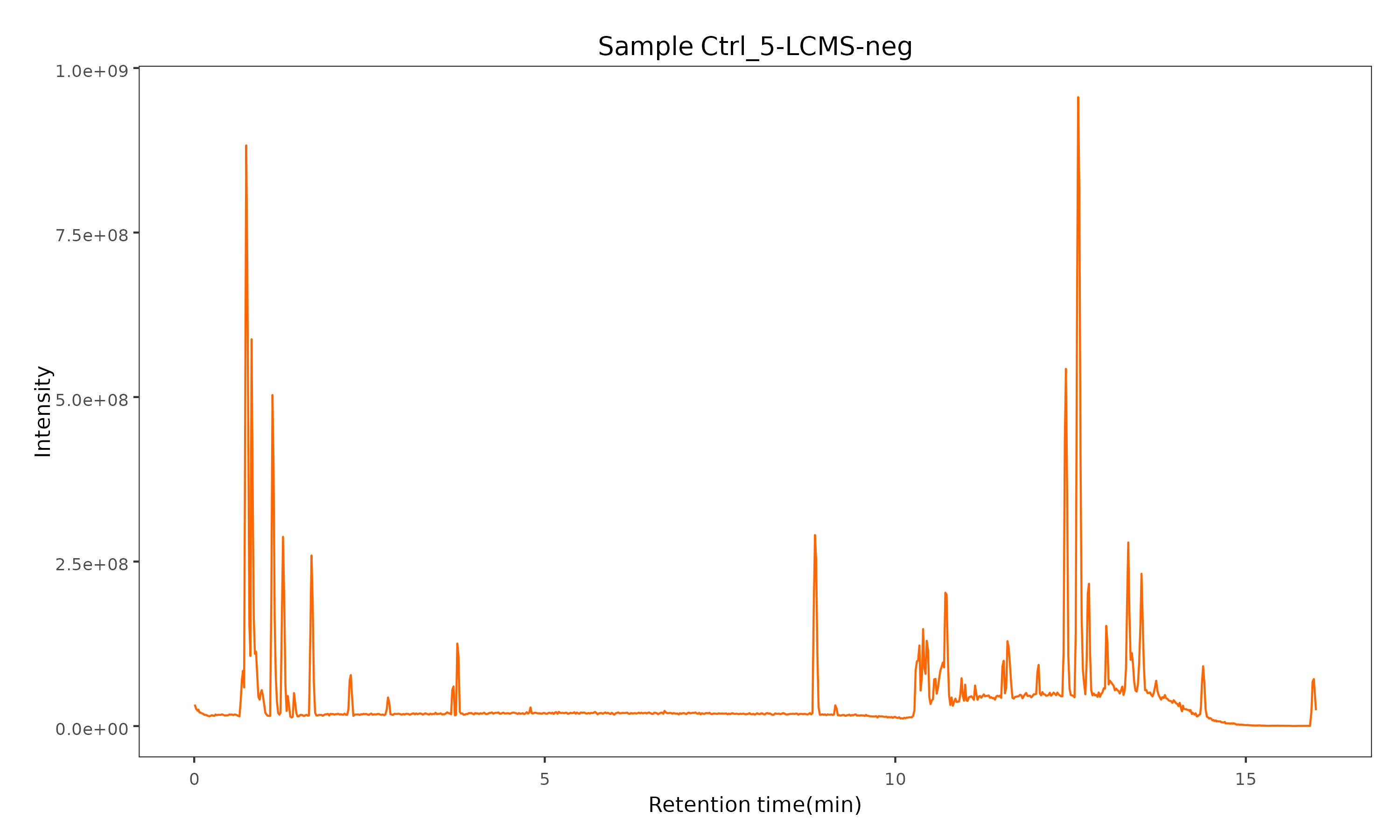

Supplement: Supplementary material S1 — The main instruments used during the LC-MS process, along with their models/specifications and manufacturers. [file Supplementary_file_1.zip › Metabolomics sequencing data FC1.2/1.基峰图/Ctrl_5-LCMS-neg-BPC.jpg]

Sample Ctrl\_5-LCMS-neg

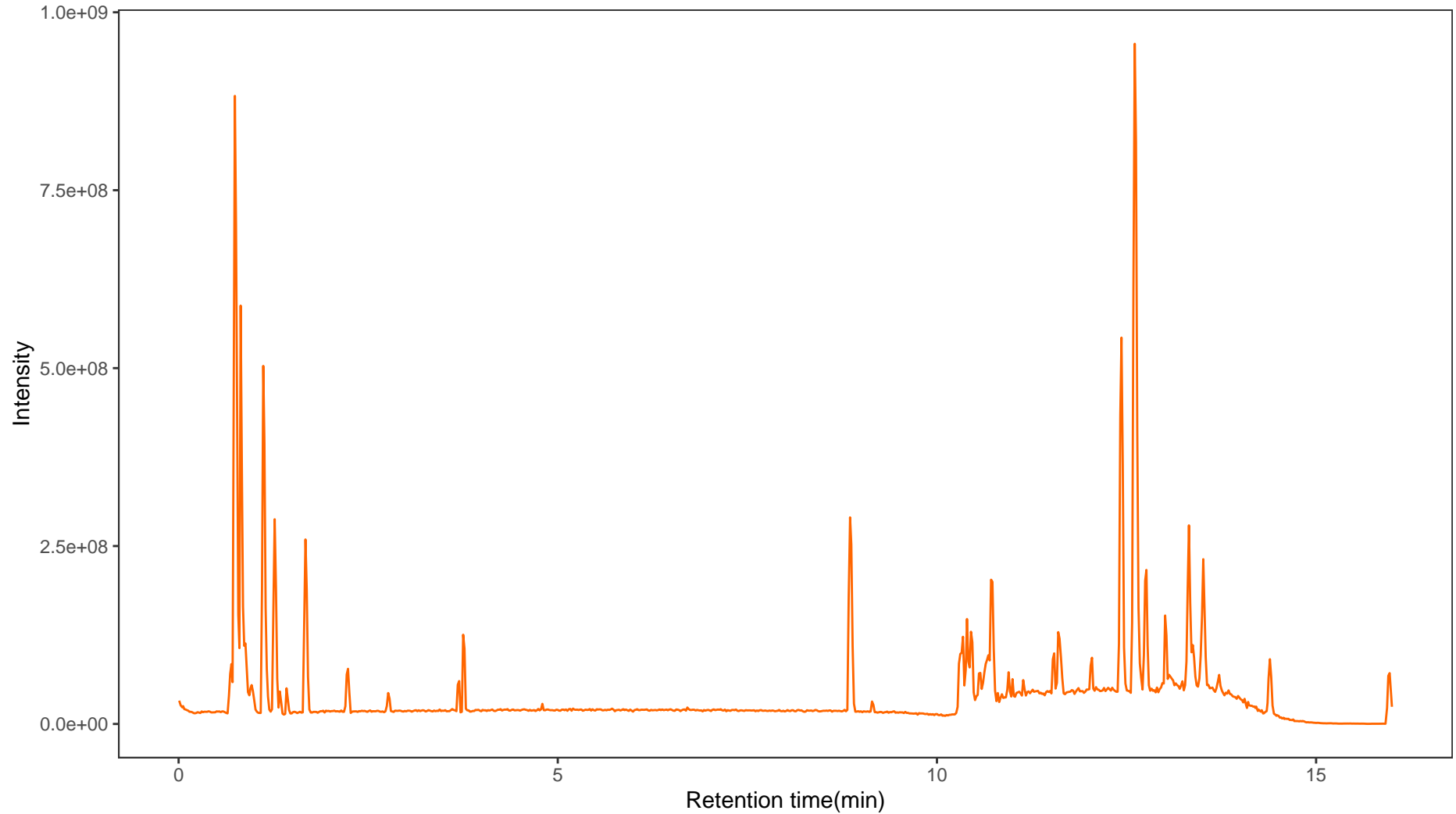

Supplement: Supplementary material S1 — The main instruments used during the LC-MS process, along with their models/specifications and manufacturers. [file Supplementary_file_1.zip › Metabolomics sequencing data FC1.2/1.基峰图/Ctrl_5-LCMS-neg-BPC.pdf]

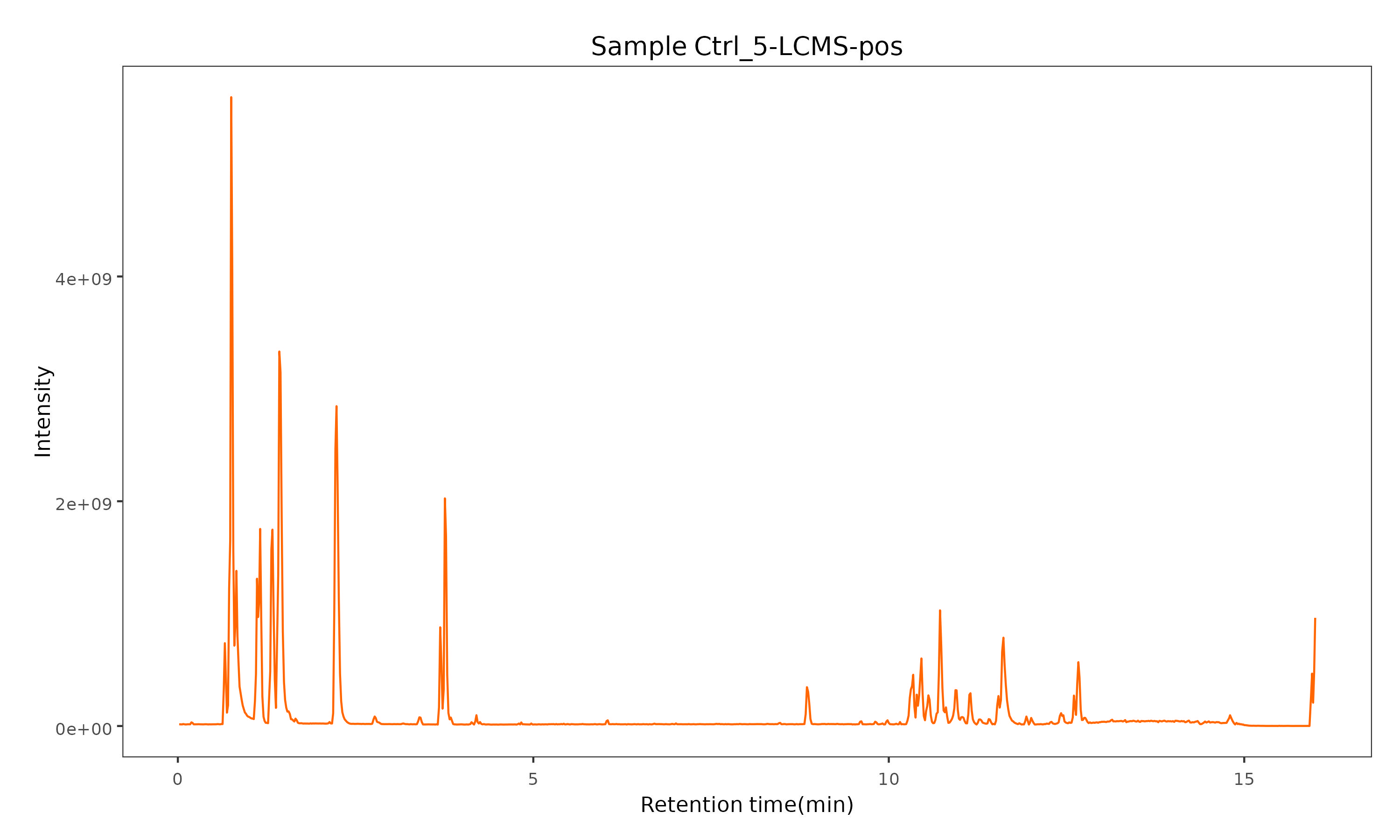

Supplement: Supplementary material S1 — The main instruments used during the LC-MS process, along with their models/specifications and manufacturers. [file Supplementary_file_1.zip › Metabolomics sequencing data FC1.2/1.基峰图/Ctrl_5-LCMS-pos-BPC.jpg]

Sample Ctrl\_5-LCMS-pos

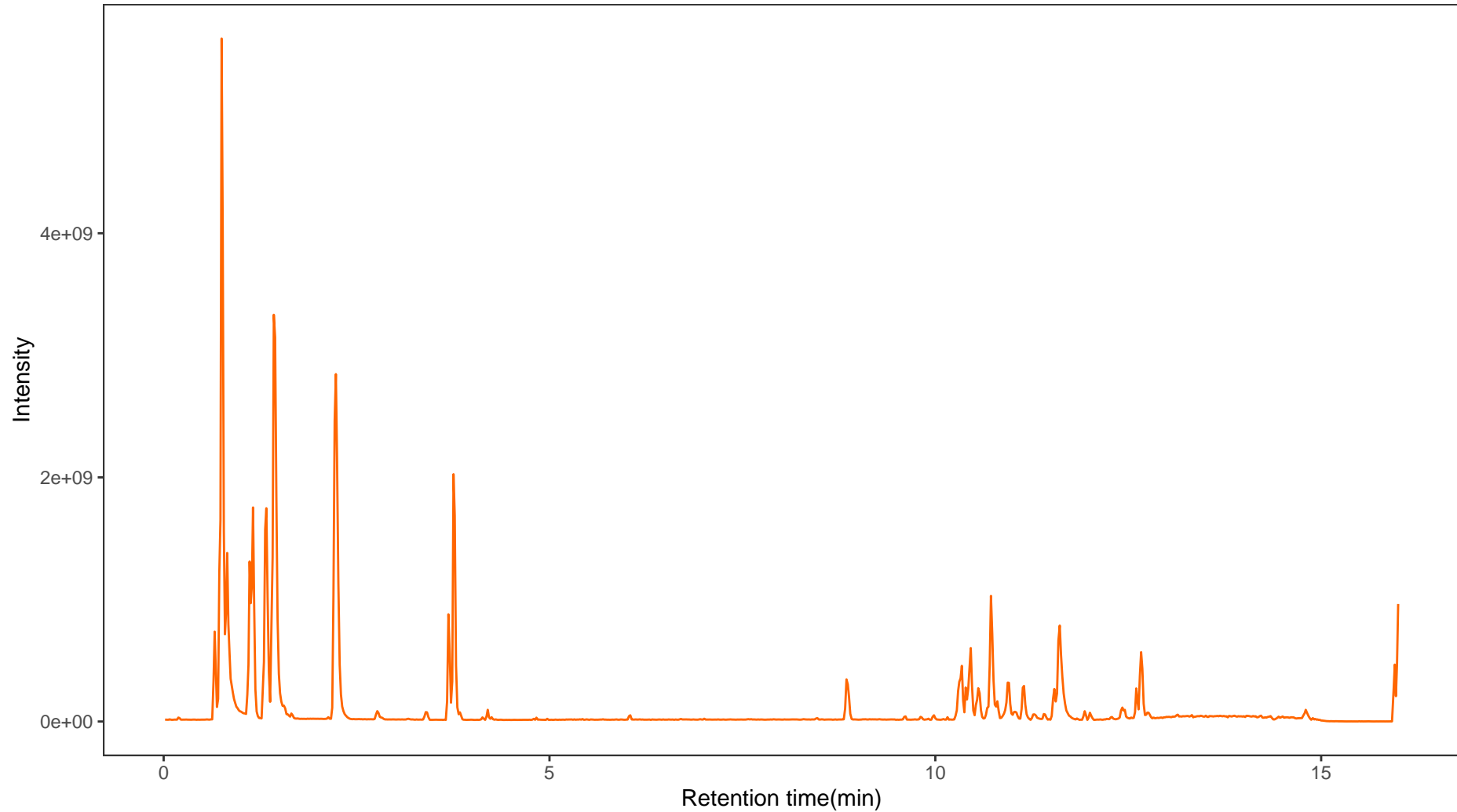

Supplement: Supplementary material S1 — The main instruments used during the LC-MS process, along with their models/specifications and manufacturers. [file Supplementary_file_1.zip › Metabolomics sequencing data FC1.2/1.基峰图/Ctrl_5-LCMS-pos-BPC.pdf]

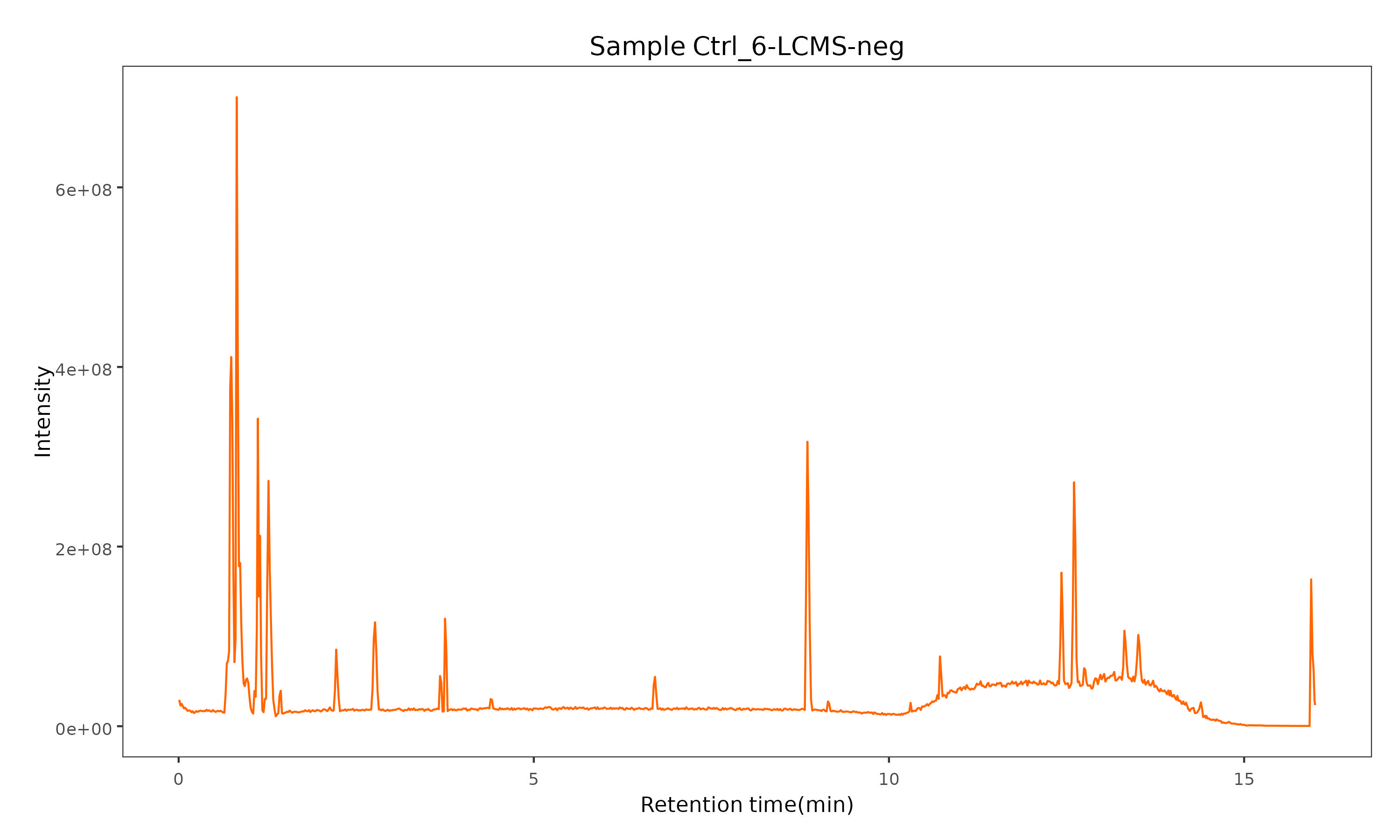

Supplement: Supplementary material S1 — The main instruments used during the LC-MS process, along with their models/specifications and manufacturers. [file Supplementary_file_1.zip › Metabolomics sequencing data FC1.2/1.基峰图/Ctrl_6-LCMS-neg-BPC.jpg]

Sample Ctrl\_6-LCMS-neg

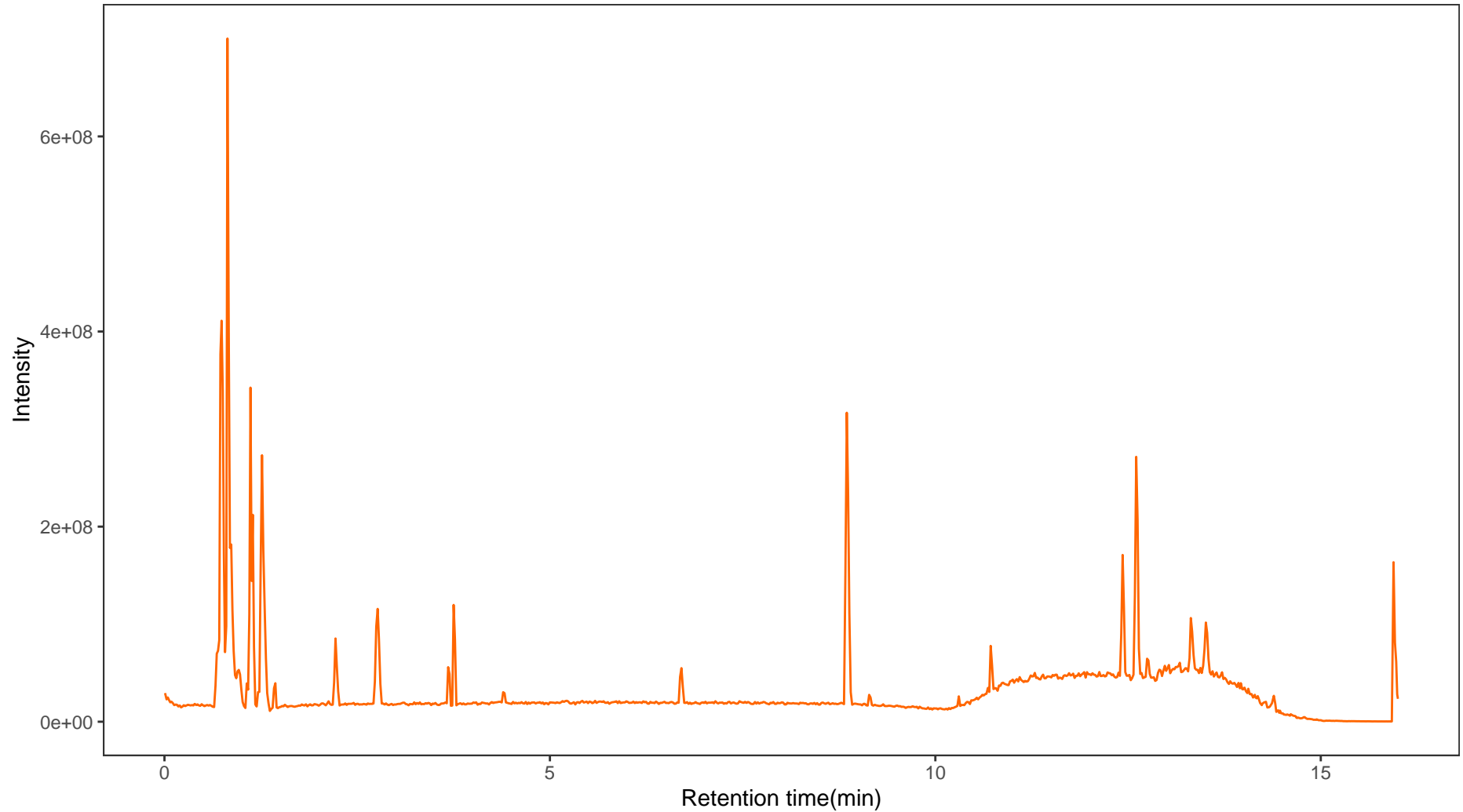

Supplement: Supplementary material S1 — The main instruments used during the LC-MS process, along with their models/specifications and manufacturers. [file Supplementary_file_1.zip › Metabolomics sequencing data FC1.2/1.基峰图/Ctrl_6-LCMS-neg-BPC.pdf]

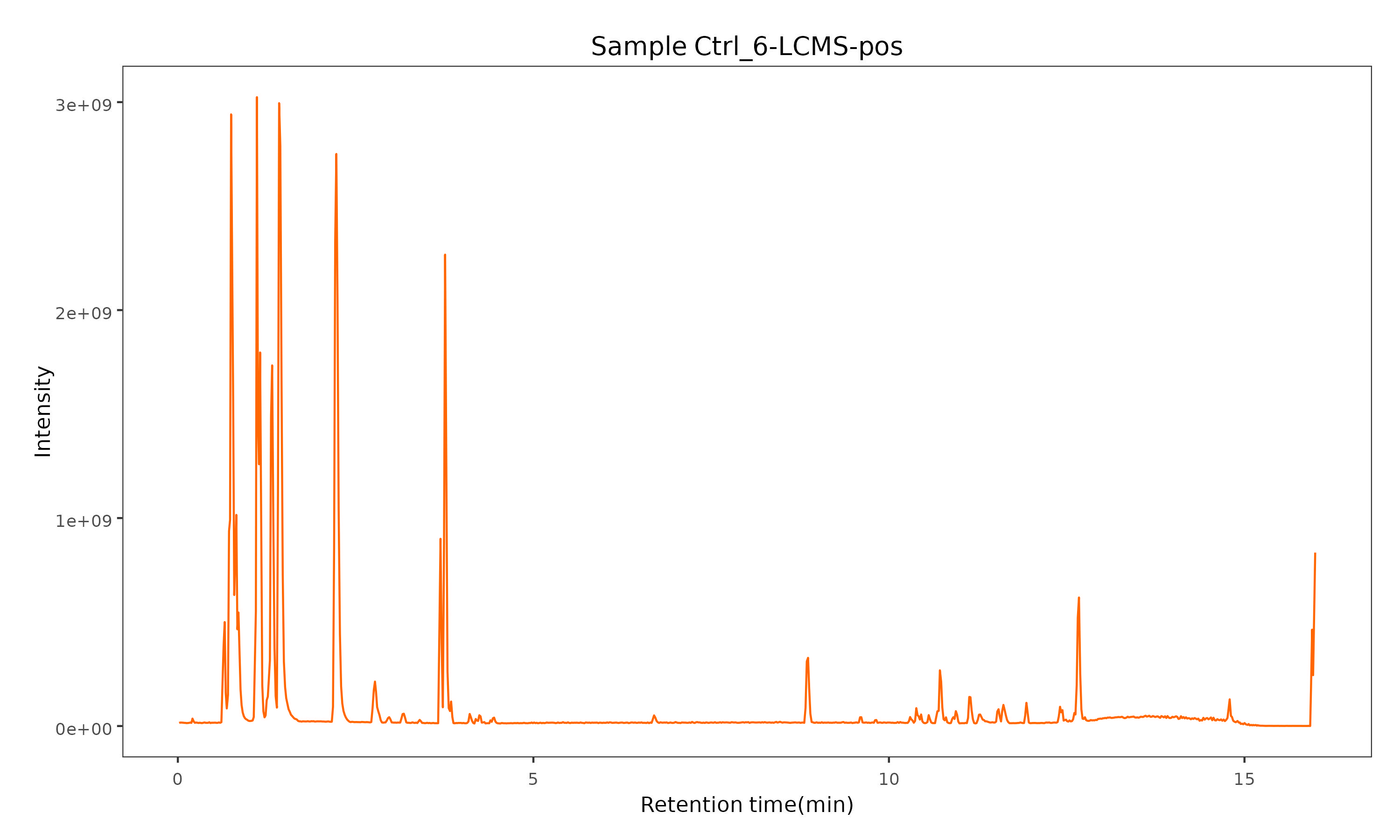

Supplement: Supplementary material S1 — The main instruments used during the LC-MS process, along with their models/specifications and manufacturers. [file Supplementary_file_1.zip › Metabolomics sequencing data FC1.2/1.基峰图/Ctrl_6-LCMS-pos-BPC.jpg]

Sample Ctrl\_6-LCMS-pos

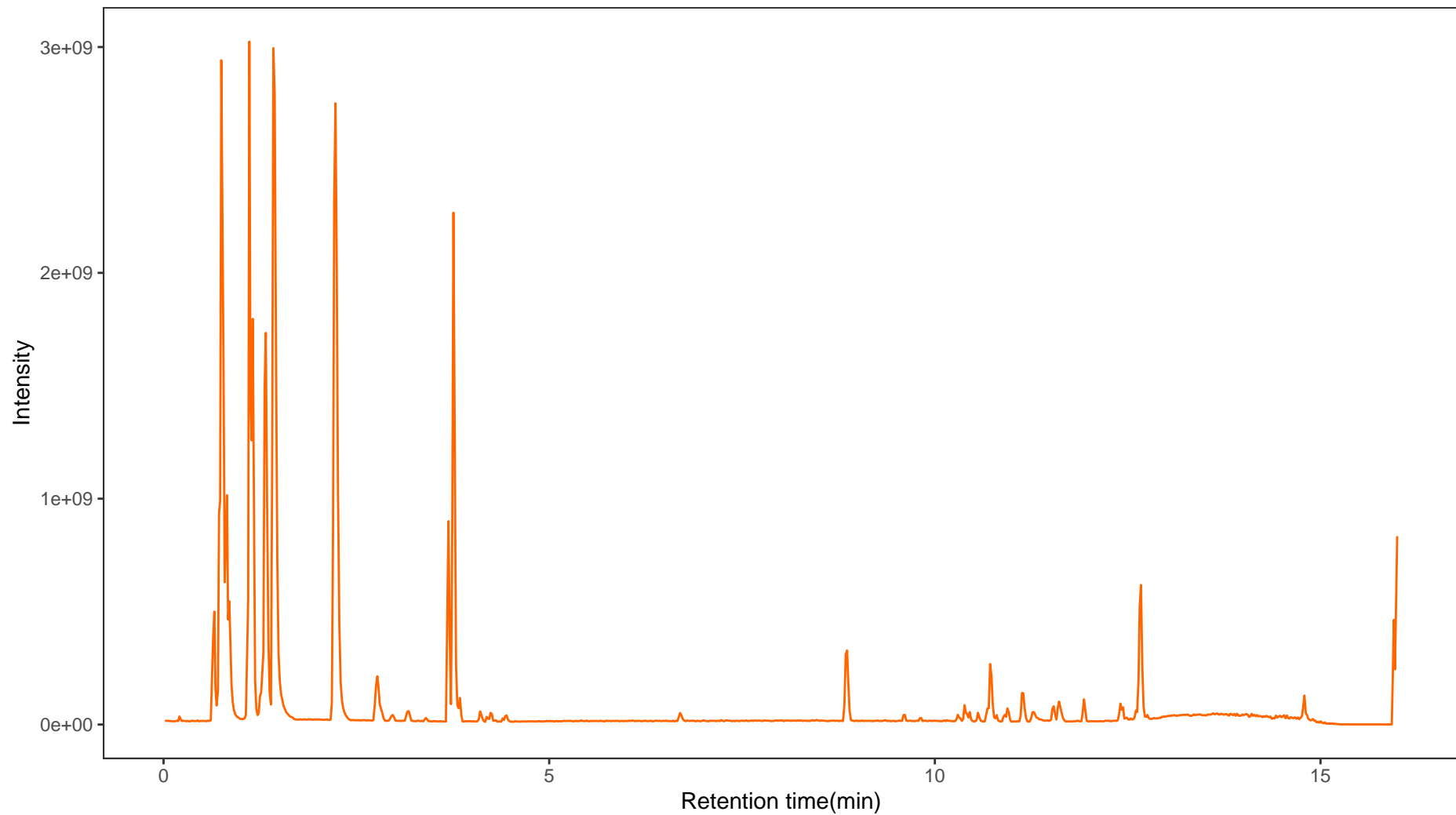

Supplement: Supplementary material S1 — The main instruments used during the LC-MS process, along with their models/specifications and manufacturers. [file Supplementary_file_1.zip › Metabolomics sequencing data FC1.2/1.基峰图/Ctrl_6-LCMS-pos-BPC.pdf]

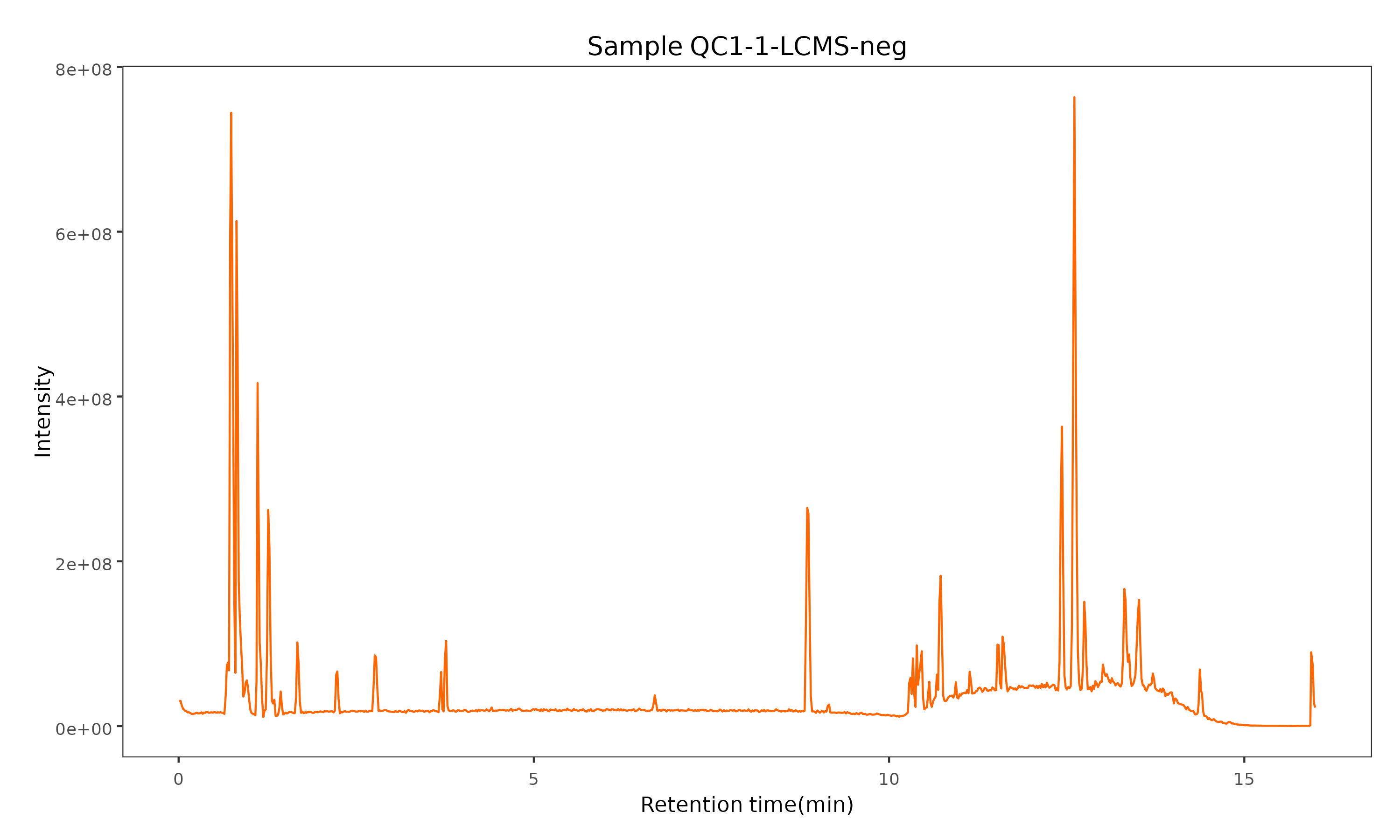

Supplement: Supplementary material S1 — The main instruments used during the LC-MS process, along with their models/specifications and manufacturers. [file Supplementary_file_1.zip › Metabolomics sequencing data FC1.2/1.基峰图/QC1-1-LCMS-neg-BPC.jpg]

Sample QC1-1-LCMS-neg

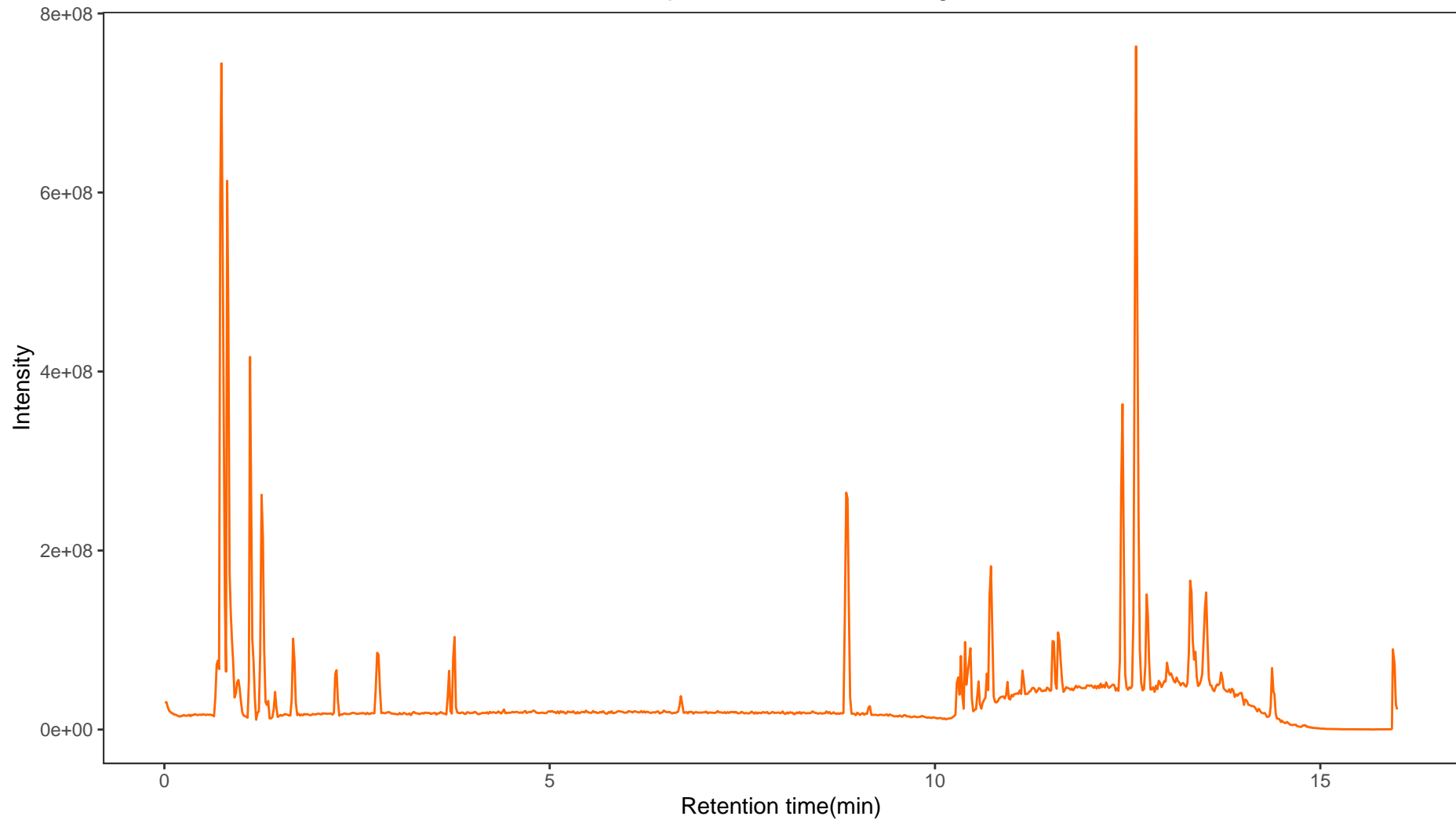

Supplement: Supplementary material S1 — The main instruments used during the LC-MS process, along with their models/specifications and manufacturers. [file Supplementary_file_1.zip › Metabolomics sequencing data FC1.2/1.基峰图/QC1-1-LCMS-neg-BPC.pdf]

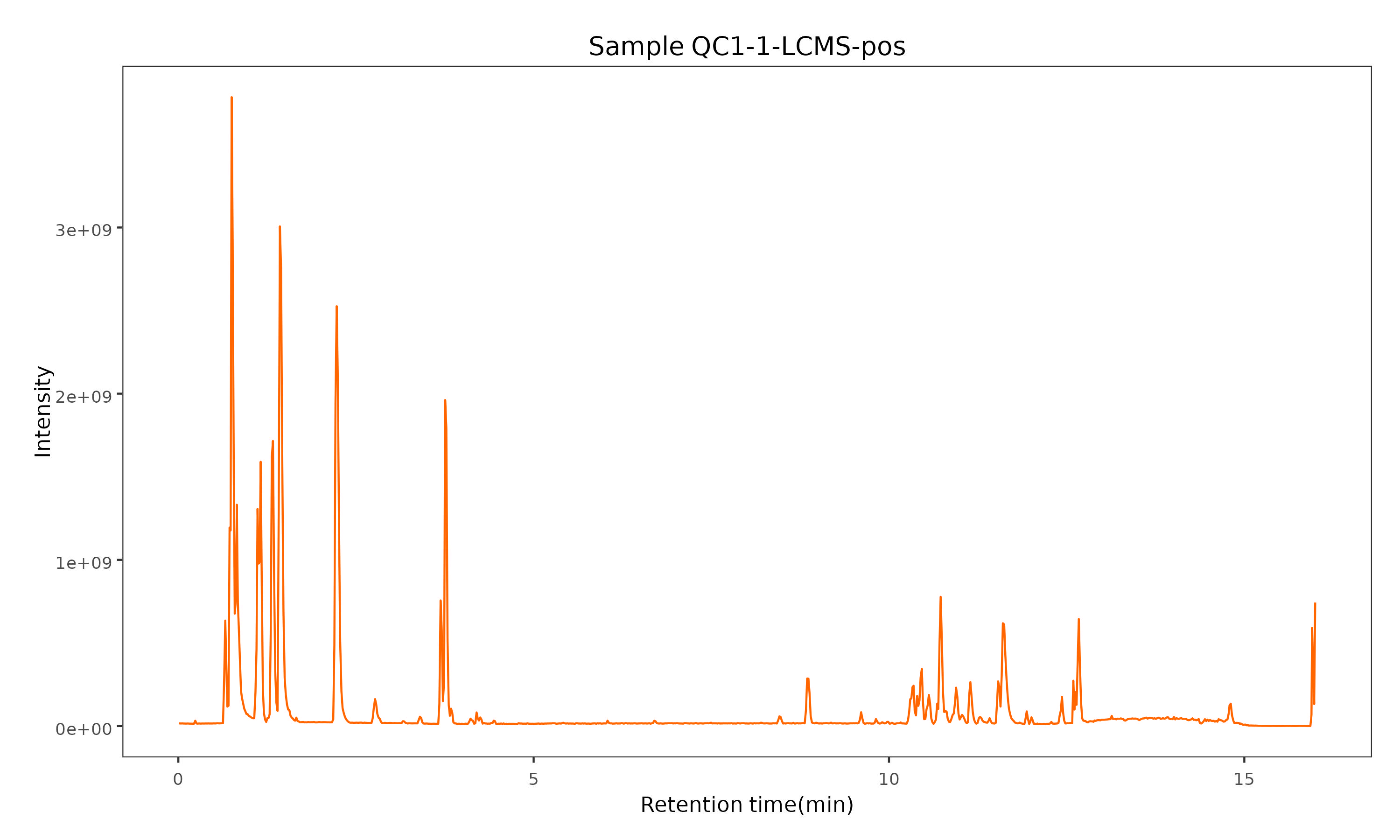

Supplement: Supplementary material S1 — The main instruments used during the LC-MS process, along with their models/specifications and manufacturers. [file Supplementary_file_1.zip › Metabolomics sequencing data FC1.2/1.基峰图/QC1-1-LCMS-pos-BPC.jpg]

Sample QC1-1-LCMS-pos

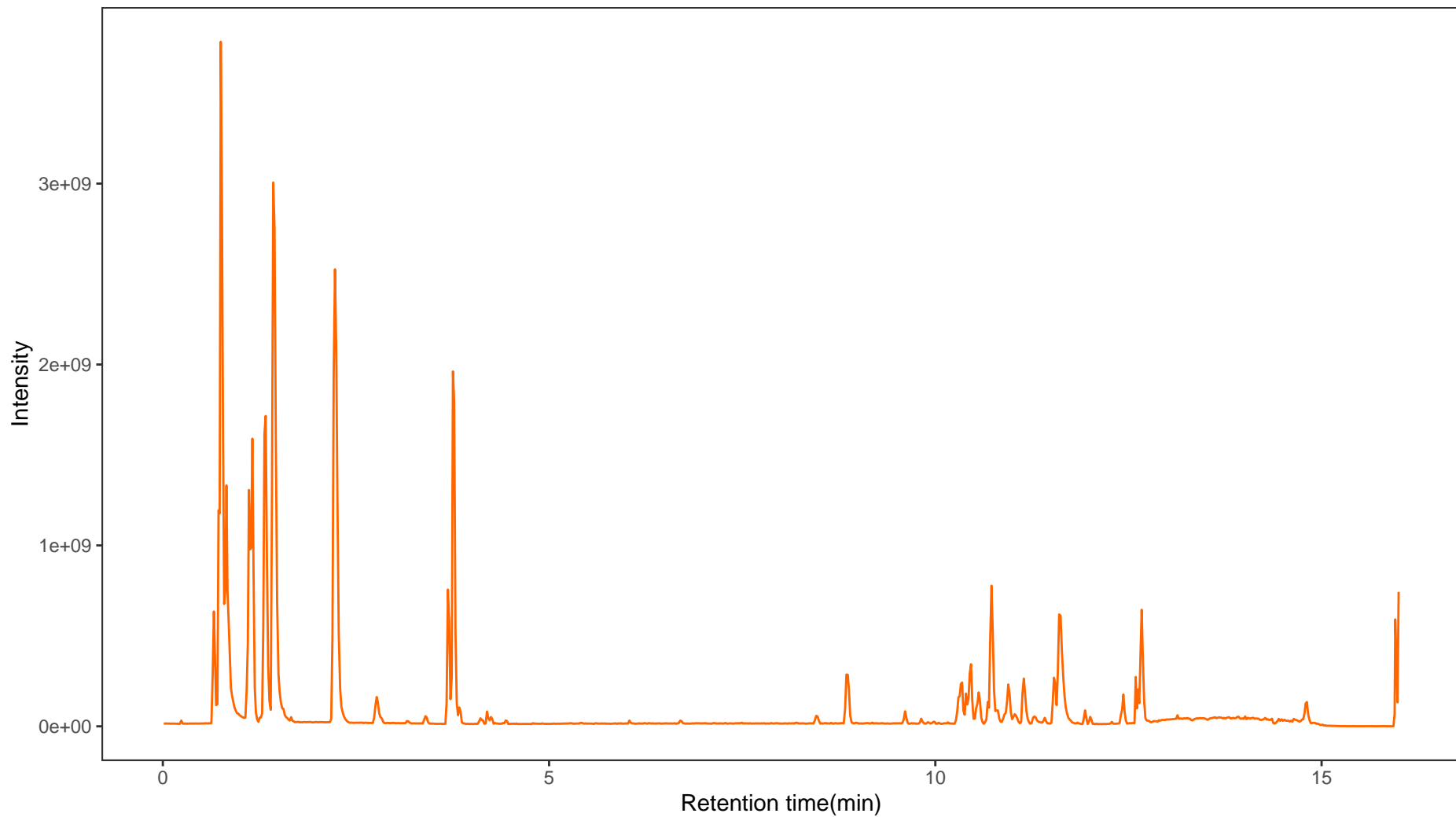

Supplement: Supplementary material S1 — The main instruments used during the LC-MS process, along with their models/specifications and manufacturers. [file Supplementary_file_1.zip › Metabolomics sequencing data FC1.2/1.基峰图/QC1-1-LCMS-pos-BPC.pdf]

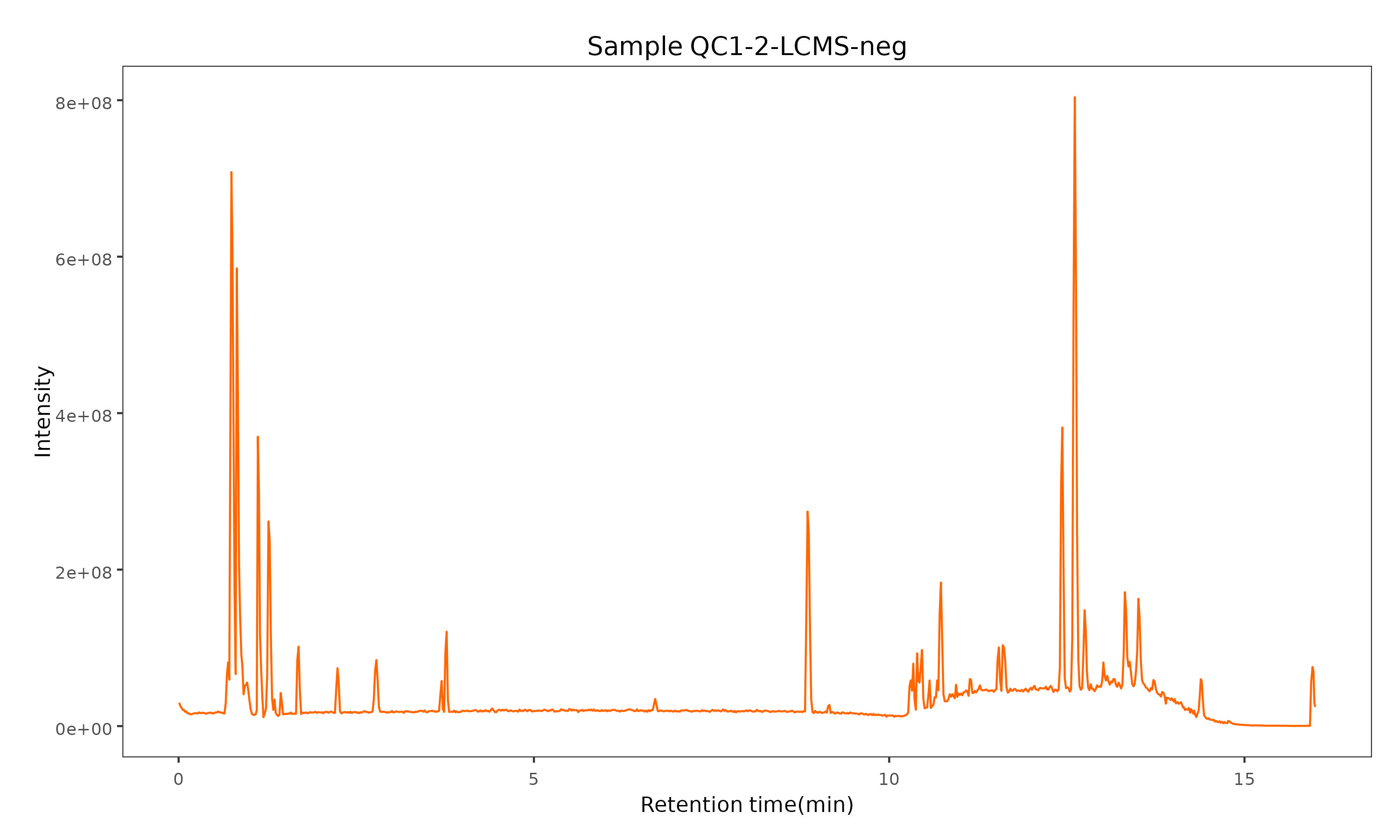

Supplement: Supplementary material S1 — The main instruments used during the LC-MS process, along with their models/specifications and manufacturers. [file Supplementary_file_1.zip › Metabolomics sequencing data FC1.2/1.基峰图/QC1-2-LCMS-neg-BPC.jpg]

Sample QC1-2-LCMS-neg

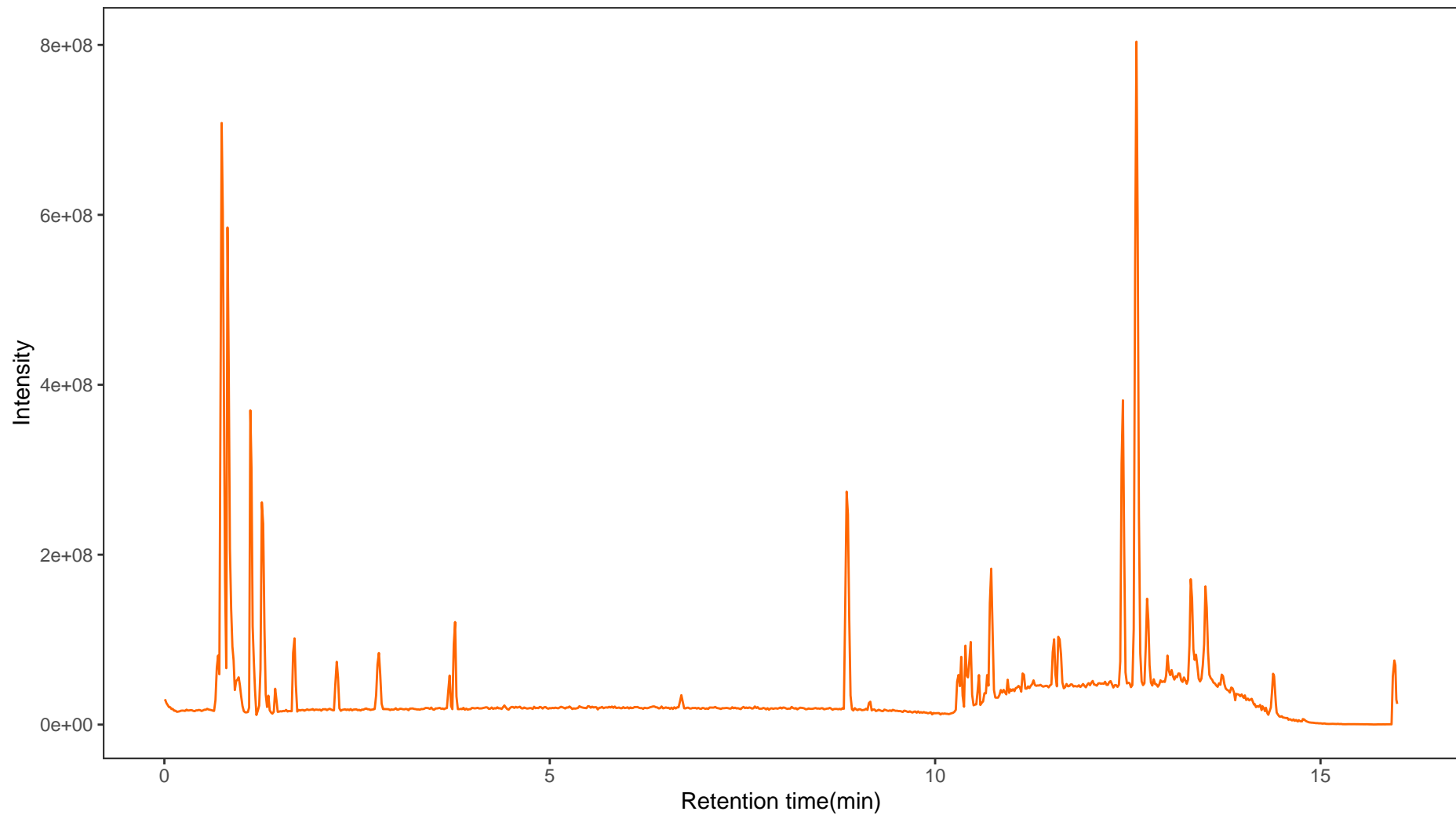

Supplement: Supplementary material S1 — The main instruments used during the LC-MS process, along with their models/specifications and manufacturers. [file Supplementary_file_1.zip › Metabolomics sequencing data FC1.2/1.基峰图/QC1-2-LCMS-neg-BPC.pdf]

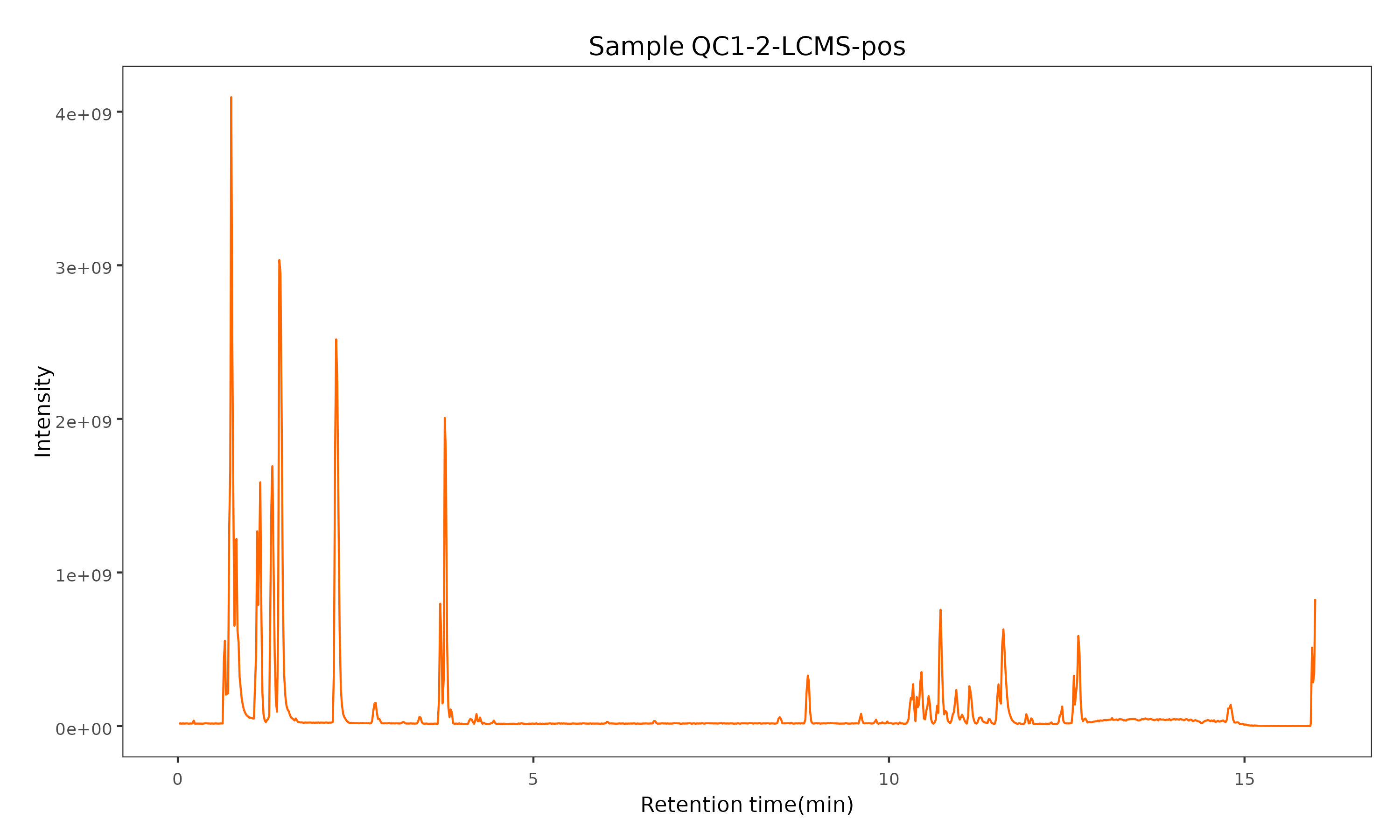

Supplement: Supplementary material S1 — The main instruments used during the LC-MS process, along with their models/specifications and manufacturers. [file Supplementary_file_1.zip › Metabolomics sequencing data FC1.2/1.基峰图/QC1-2-LCMS-pos-BPC.jpg]

Sample QC1-2-LCMS-pos

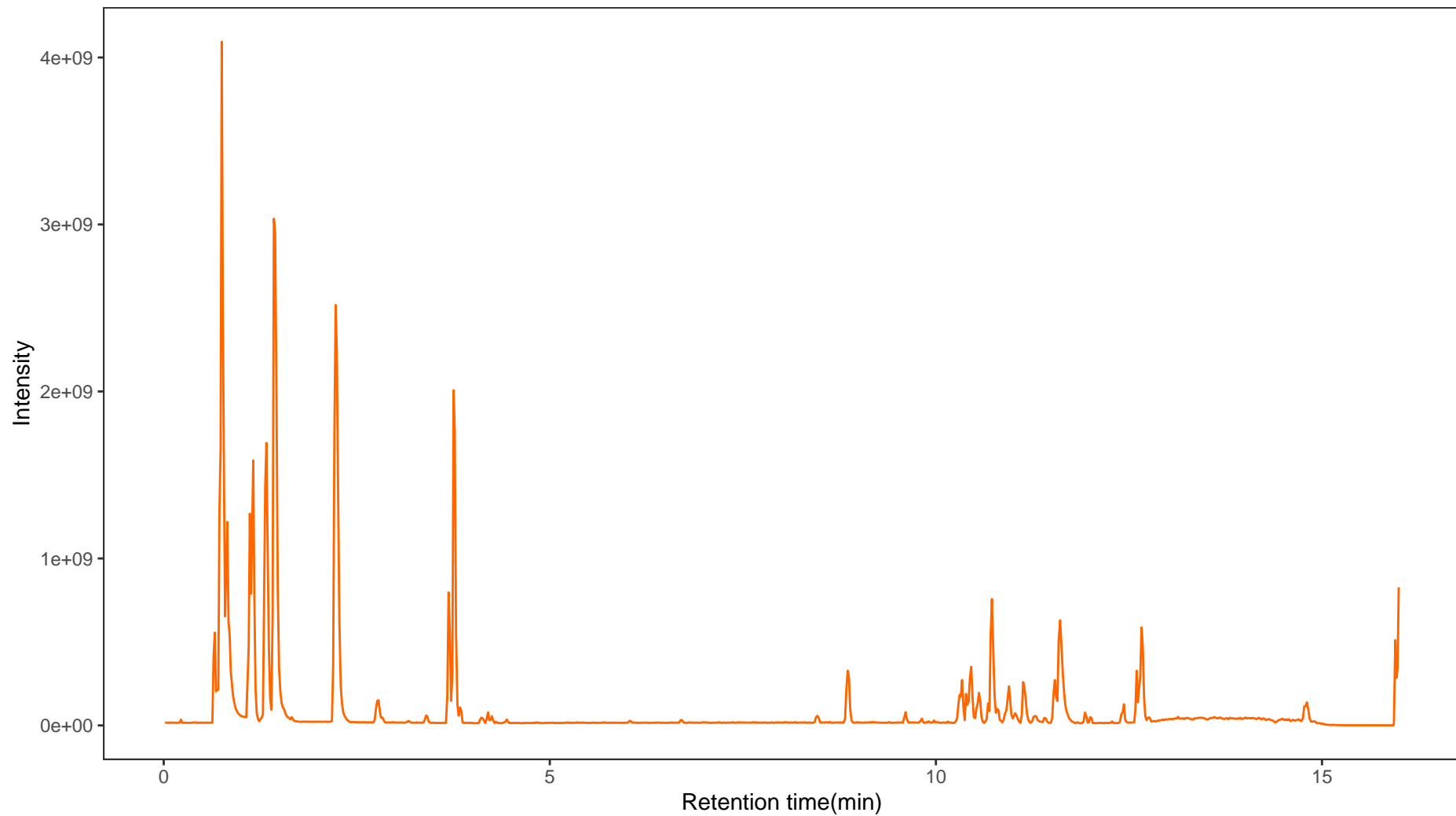

Supplement: Supplementary material S1 — The main instruments used during the LC-MS process, along with their models/specifications and manufacturers. [file Supplementary_file_1.zip › Metabolomics sequencing data FC1.2/1.基峰图/QC1-2-LCMS-pos-BPC.pdf]

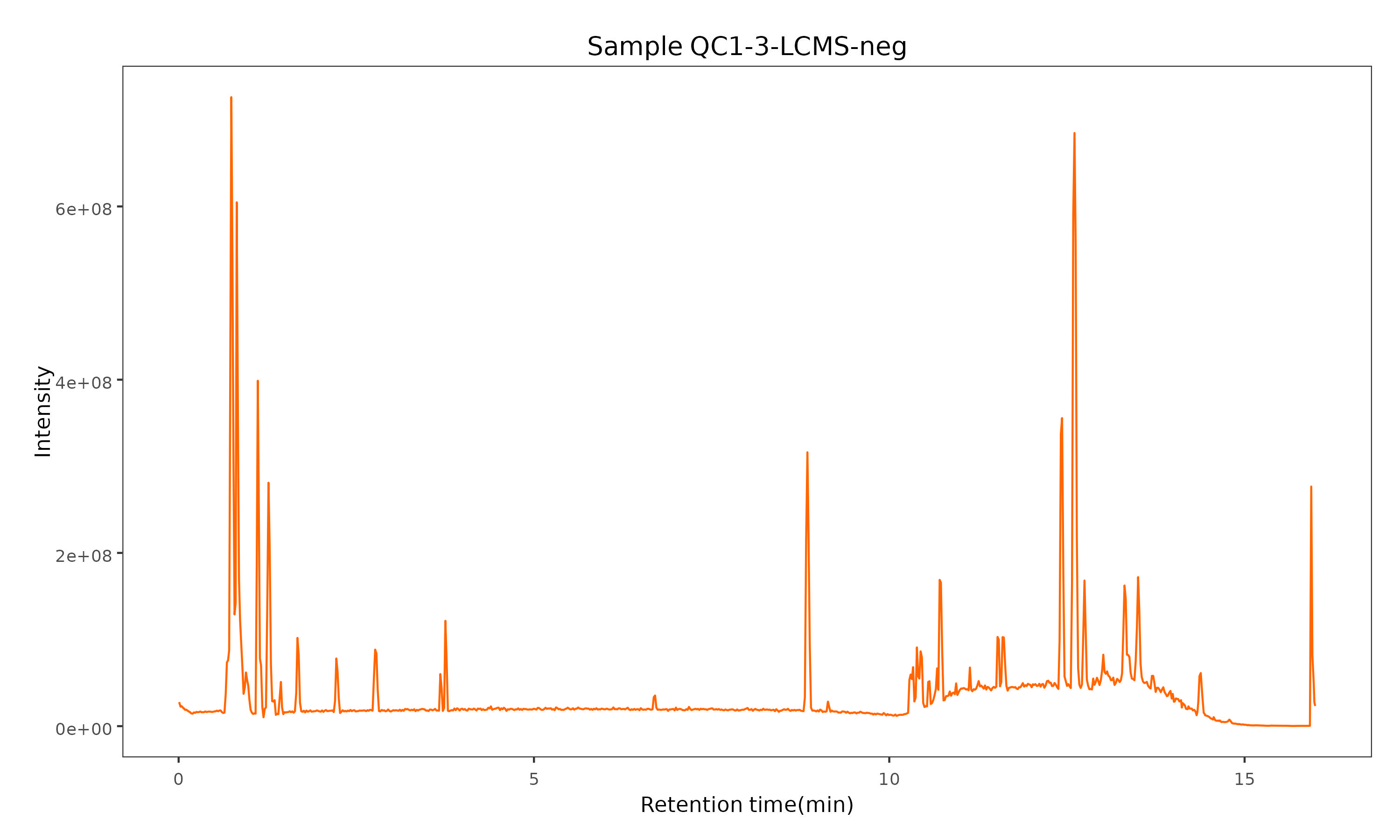

Supplement: Supplementary material S1 — The main instruments used during the LC-MS process, along with their models/specifications and manufacturers. [file Supplementary_file_1.zip › Metabolomics sequencing data FC1.2/1.基峰图/QC1-3-LCMS-neg-BPC.jpg]

Sample QC1-3-LCMS-neg

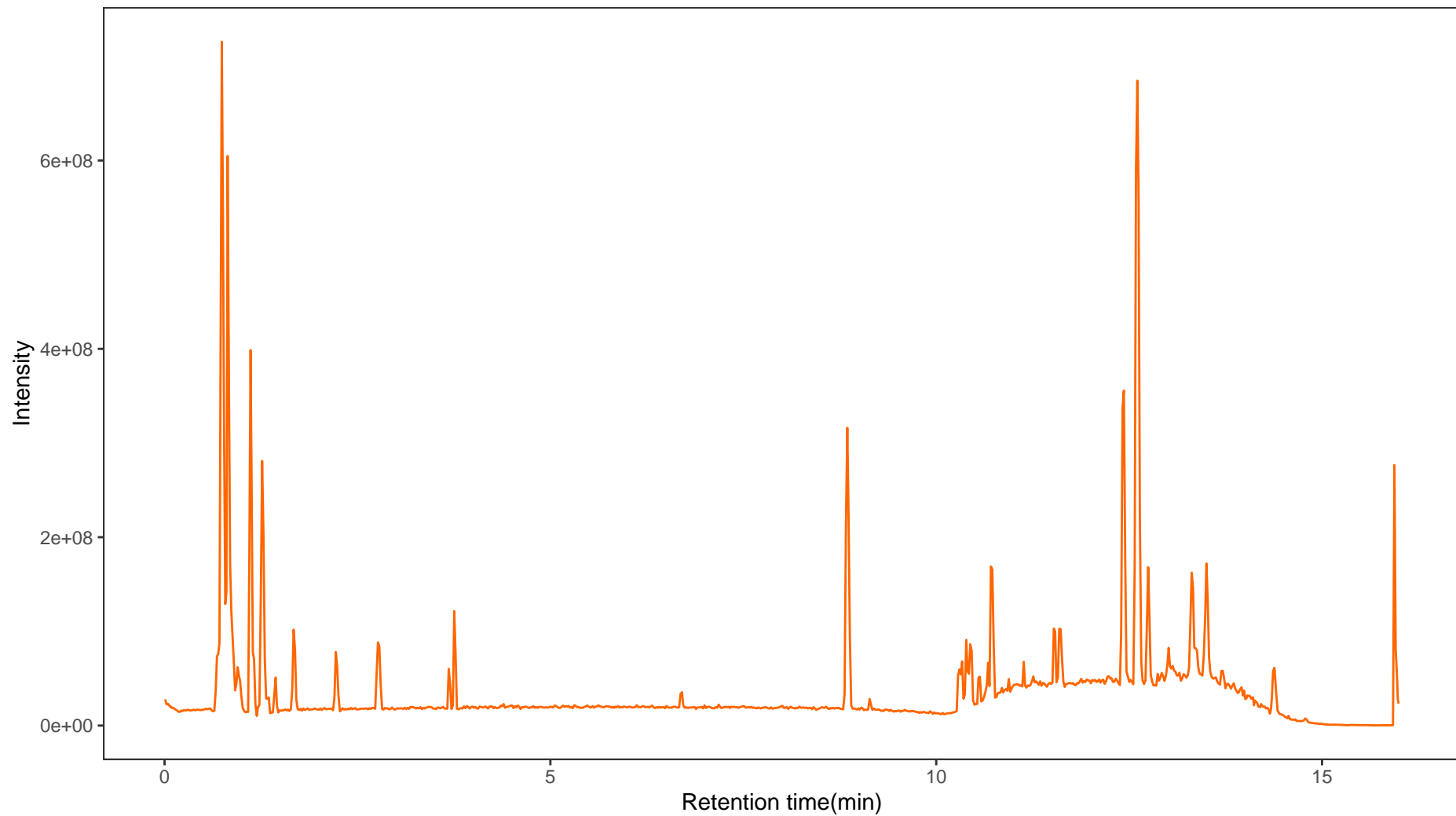

Supplement: Supplementary material S1 — The main instruments used during the LC-MS process, along with their models/specifications and manufacturers. [file Supplementary_file_1.zip › Metabolomics sequencing data FC1.2/1.基峰图/QC1-3-LCMS-neg-BPC.pdf]

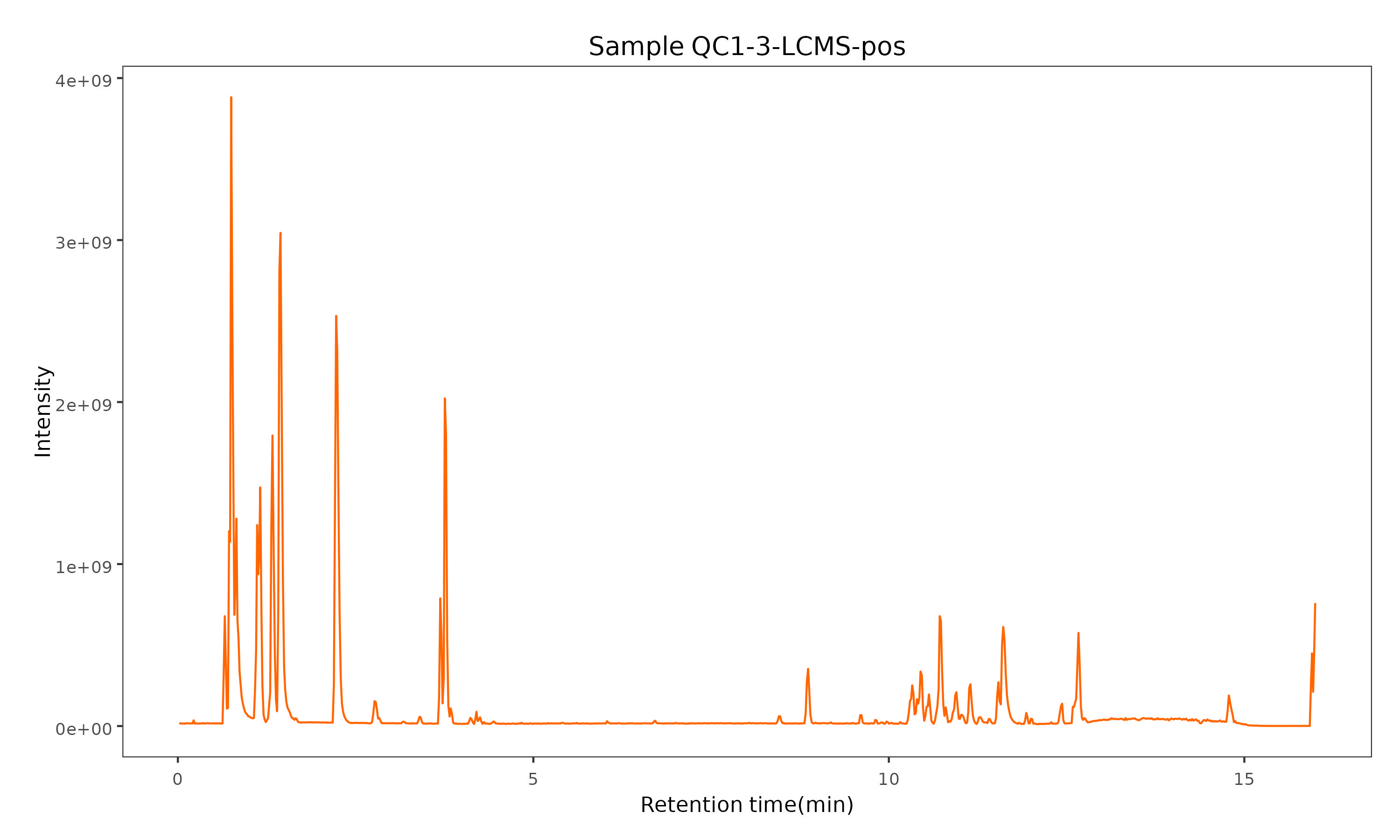

Supplement: Supplementary material S1 — The main instruments used during the LC-MS process, along with their models/specifications and manufacturers. [file Supplementary_file_1.zip › Metabolomics sequencing data FC1.2/1.基峰图/QC1-3-LCMS-pos-BPC.jpg]

Sample QC1-3-LCMS-pos

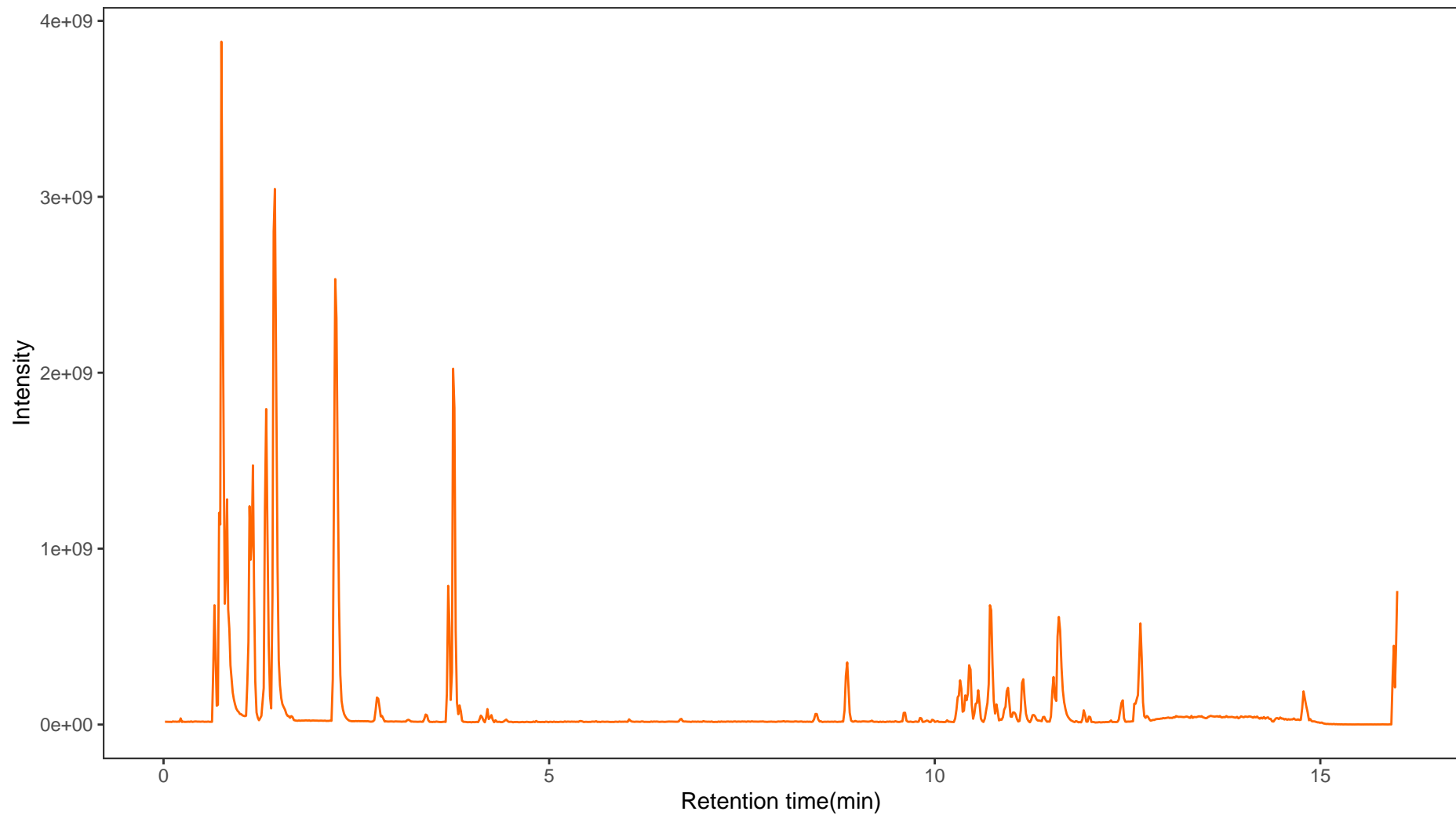

Supplement: Supplementary material S1 — The main instruments used during the LC-MS process, along with their models/specifications and manufacturers. [file Supplementary_file_1.zip › Metabolomics sequencing data FC1.2/1.基峰图/QC1-3-LCMS-pos-BPC.pdf]

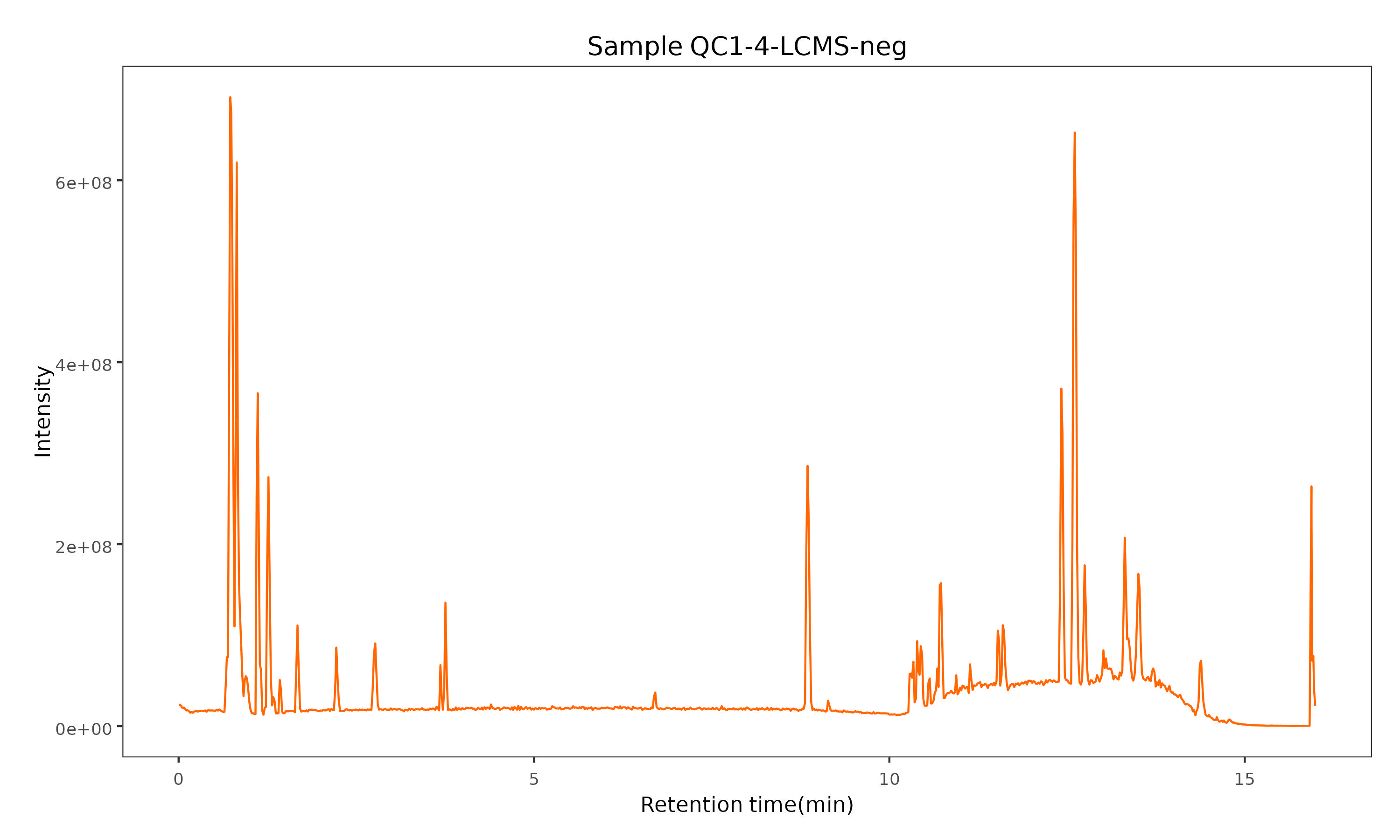

Supplement: Supplementary material S1 — The main instruments used during the LC-MS process, along with their models/specifications and manufacturers. [file Supplementary_file_1.zip › Metabolomics sequencing data FC1.2/1.基峰图/QC1-4-LCMS-neg-BPC.jpg]

Sample QC1-4-LCMS-neg

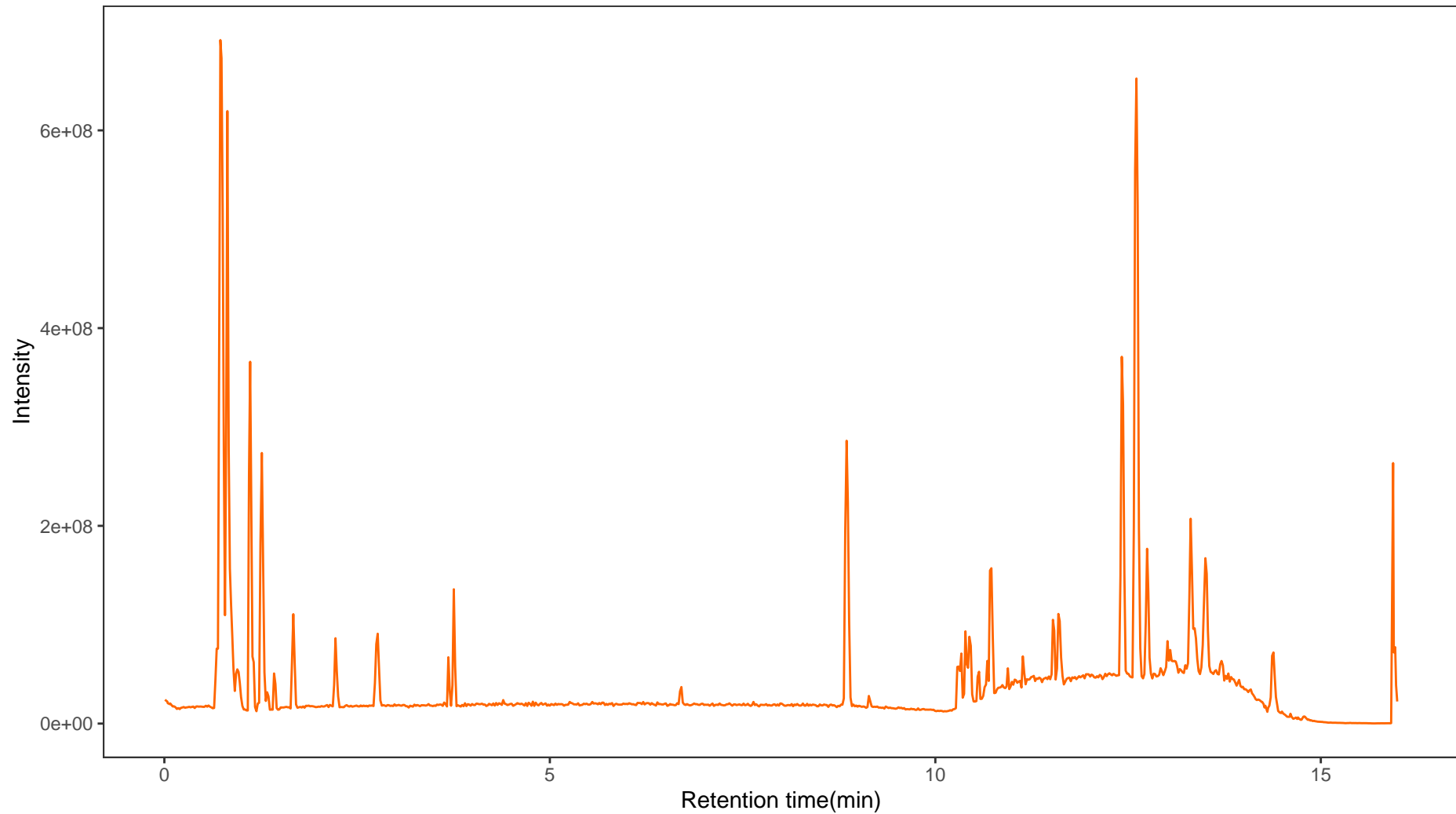

Supplement: Supplementary material S1 — The main instruments used during the LC-MS process, along with their models/specifications and manufacturers. [file Supplementary_file_1.zip › Metabolomics sequencing data FC1.2/1.基峰图/QC1-4-LCMS-neg-BPC.pdf]

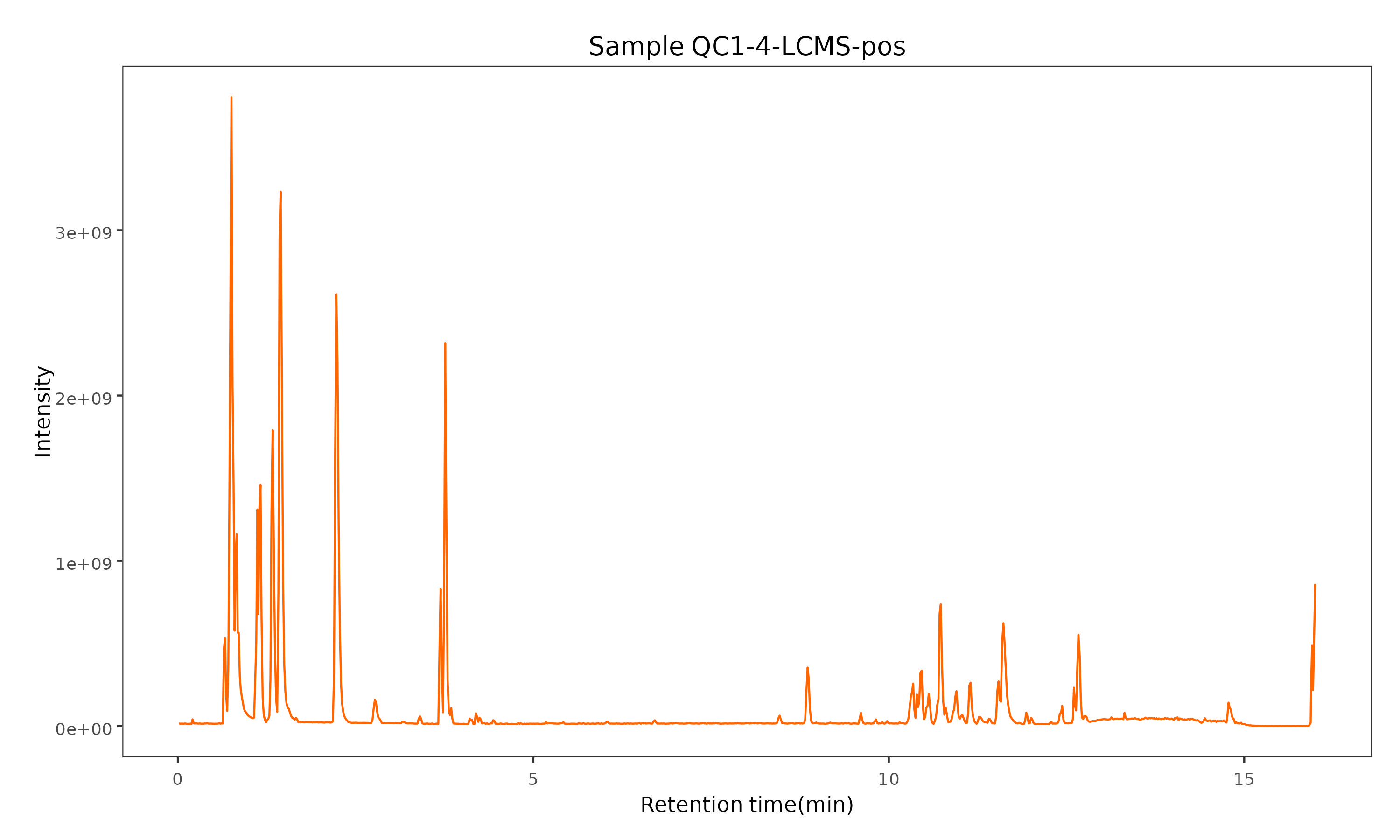

Supplement: Supplementary material S1 — The main instruments used during the LC-MS process, along with their models/specifications and manufacturers. [file Supplementary_file_1.zip › Metabolomics sequencing data FC1.2/1.基峰图/QC1-4-LCMS-pos-BPC.jpg]

Sample QC1-4-LCMS-pos

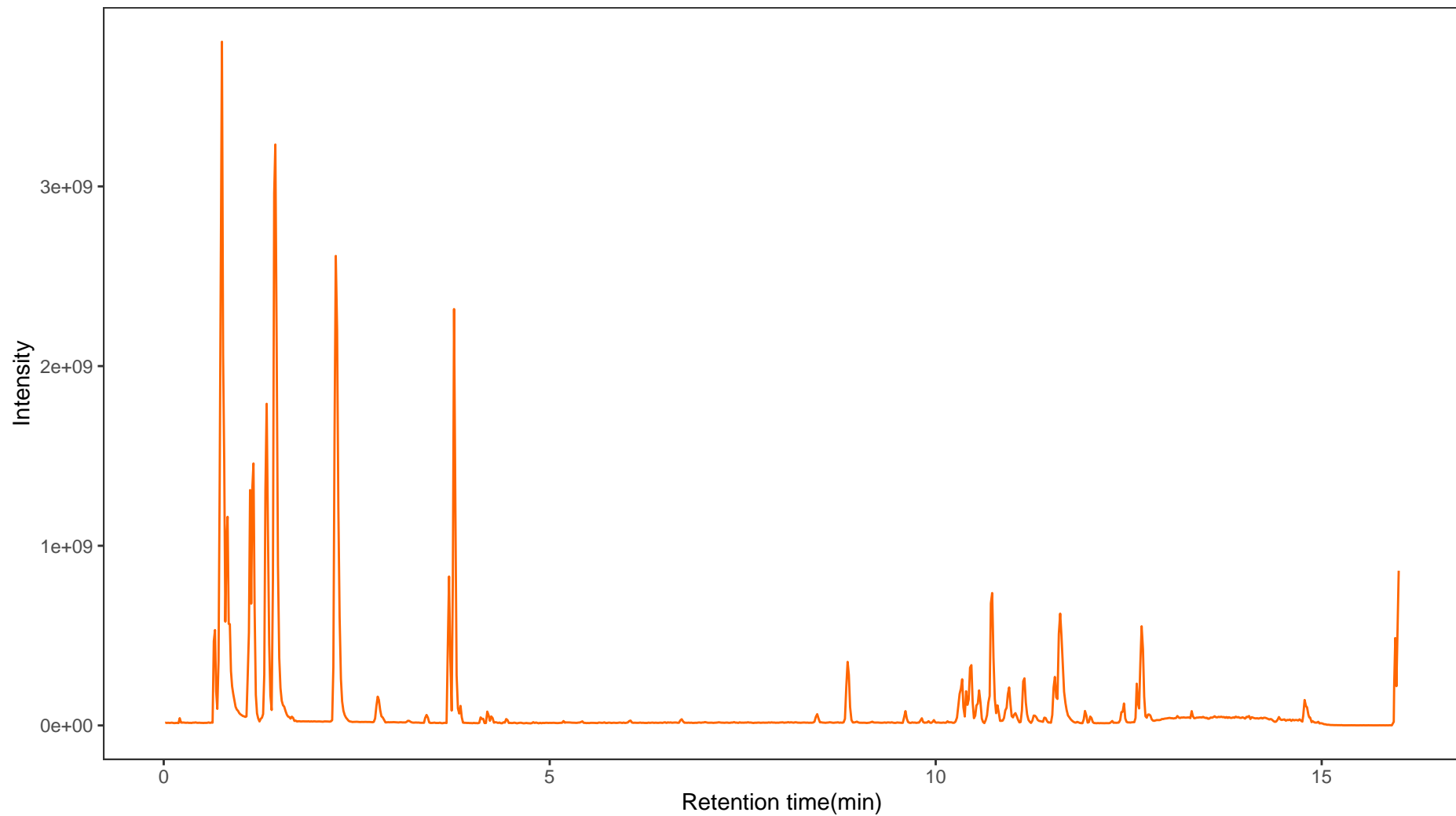

Supplement: Supplementary material S1 — The main instruments used during the LC-MS process, along with their models/specifications and manufacturers. [file Supplementary_file_1.zip › Metabolomics sequencing data FC1.2/1.基峰图/QC1-4-LCMS-pos-BPC.pdf]

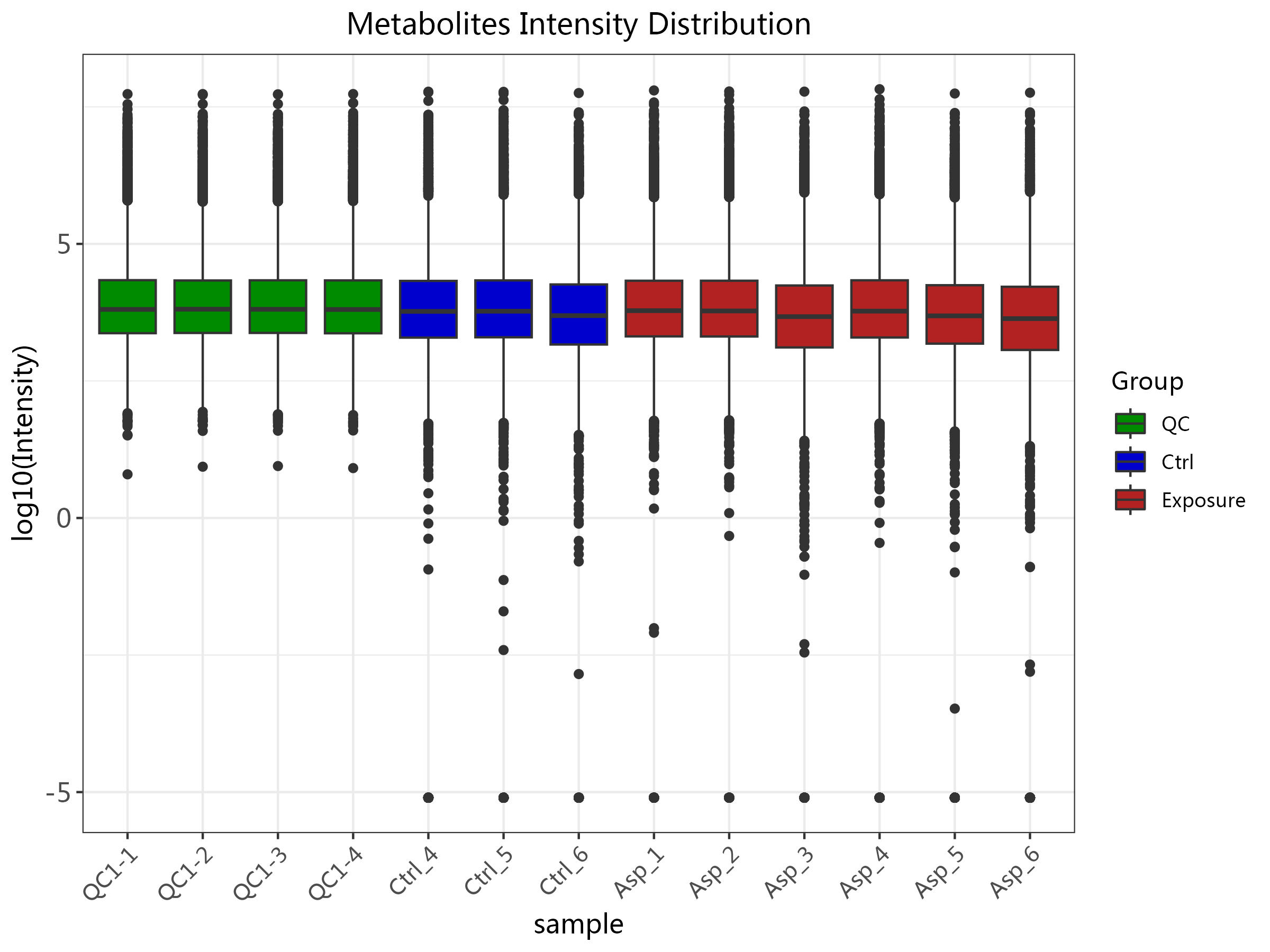

Supplement: Supplementary material S1 — The main instruments used during the LC-MS process, along with their models/specifications and manufacturers. [file Supplementary_file_1.zip › Metabolomics sequencing data FC1.2/2.质量控制/Intensity Distribution.jpg]

# Metabolites Intensity Distribution

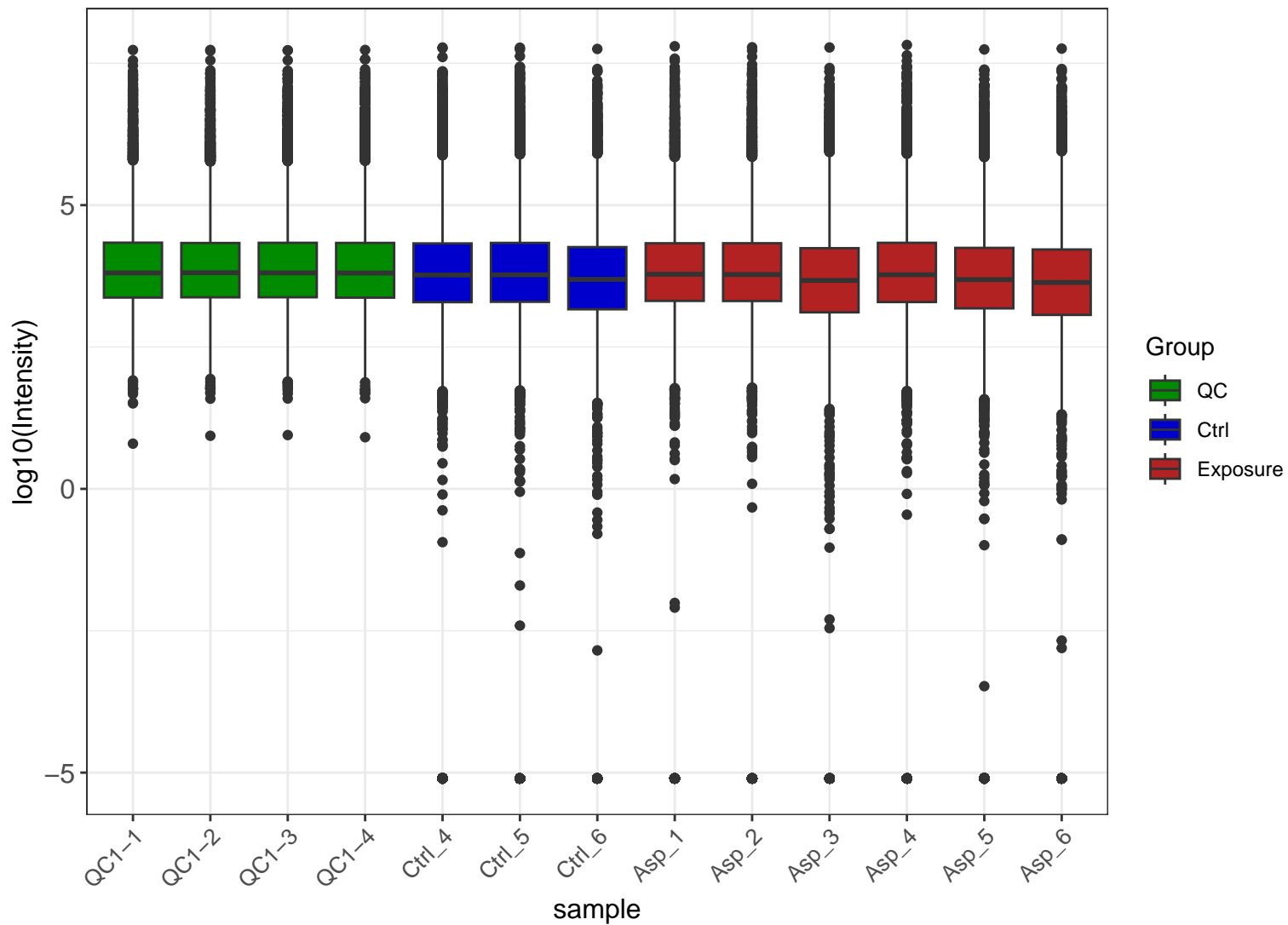

Supplement: Supplementary material S1 — The main instruments used during the LC-MS process, along with their models/specifications and manufacturers. [file Supplementary_file_1.zip › Metabolomics sequencing data FC1.2/2.质量控制/Intensity Distribution.pdf]

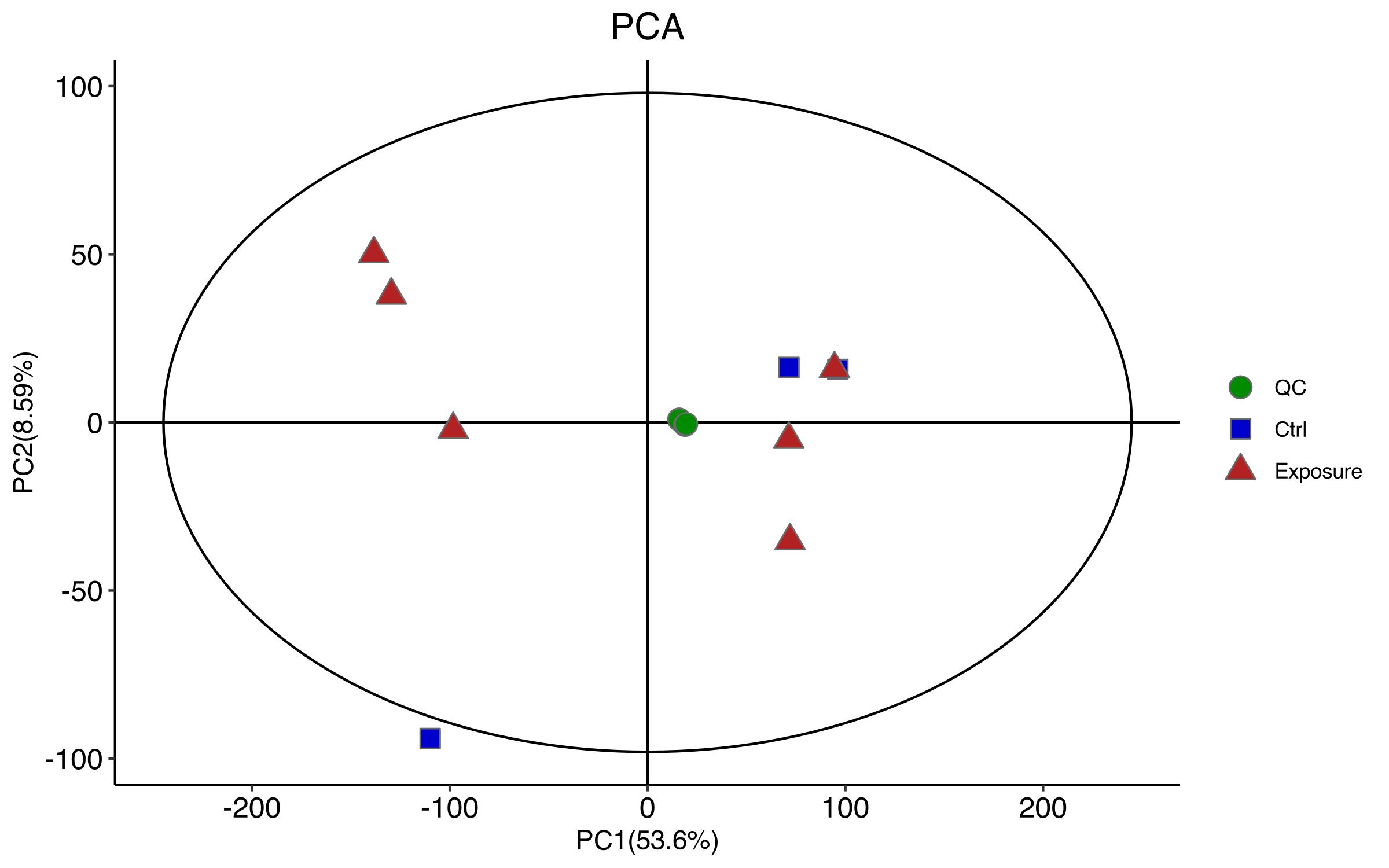

Supplement: Supplementary material S1 — The main instruments used during the LC-MS process, along with their models/specifications and manufacturers. [file Supplementary_file_1.zip › Metabolomics sequencing data FC1.2/2.质量控制/PCA(ALL).jpg]

# PCA

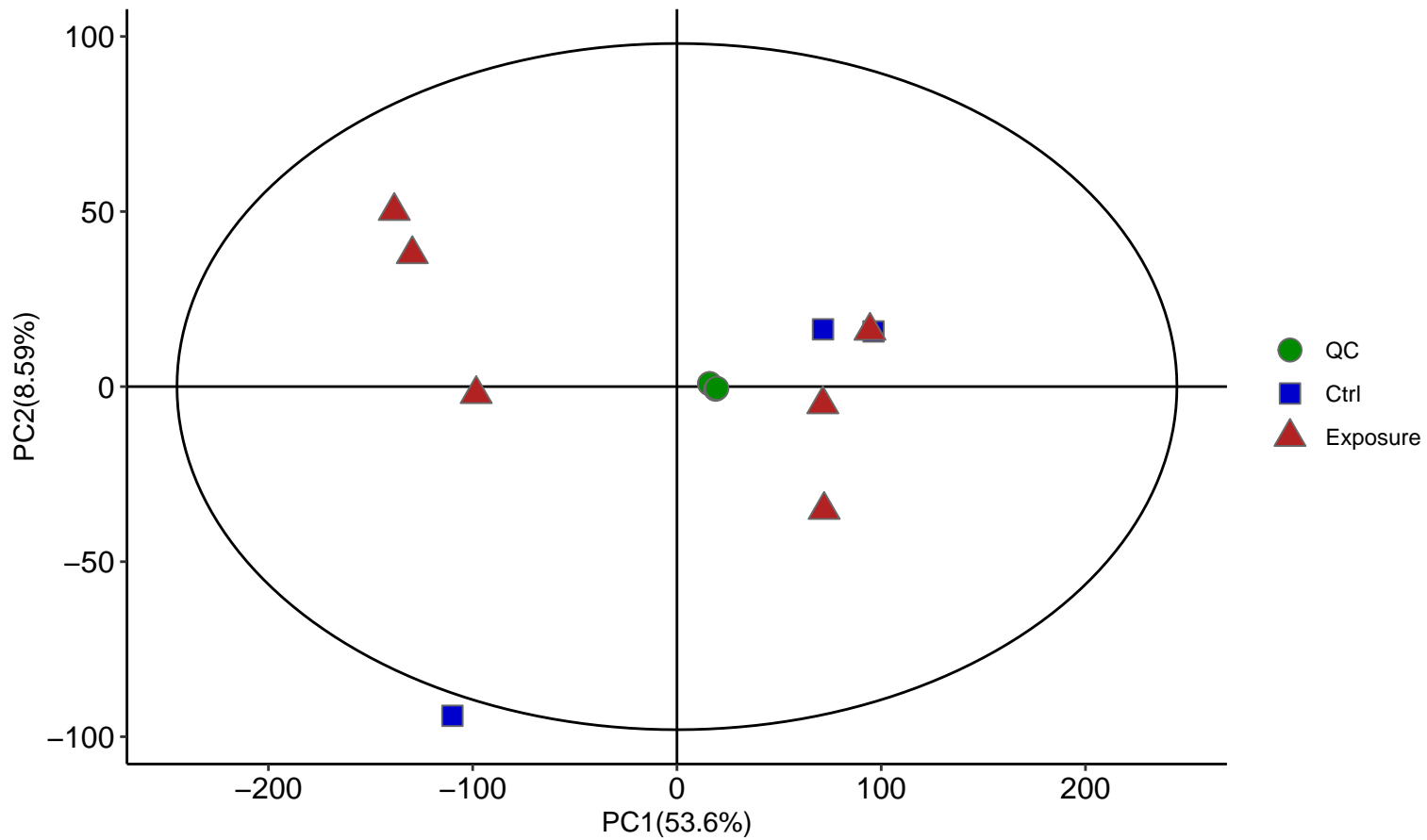

Supplement: Supplementary material S1 — The main instruments used during the LC-MS process, along with their models/specifications and manufacturers. [file Supplementary_file_1.zip › Metabolomics sequencing data FC1.2/2.质量控制/PCA(ALL).pdf]

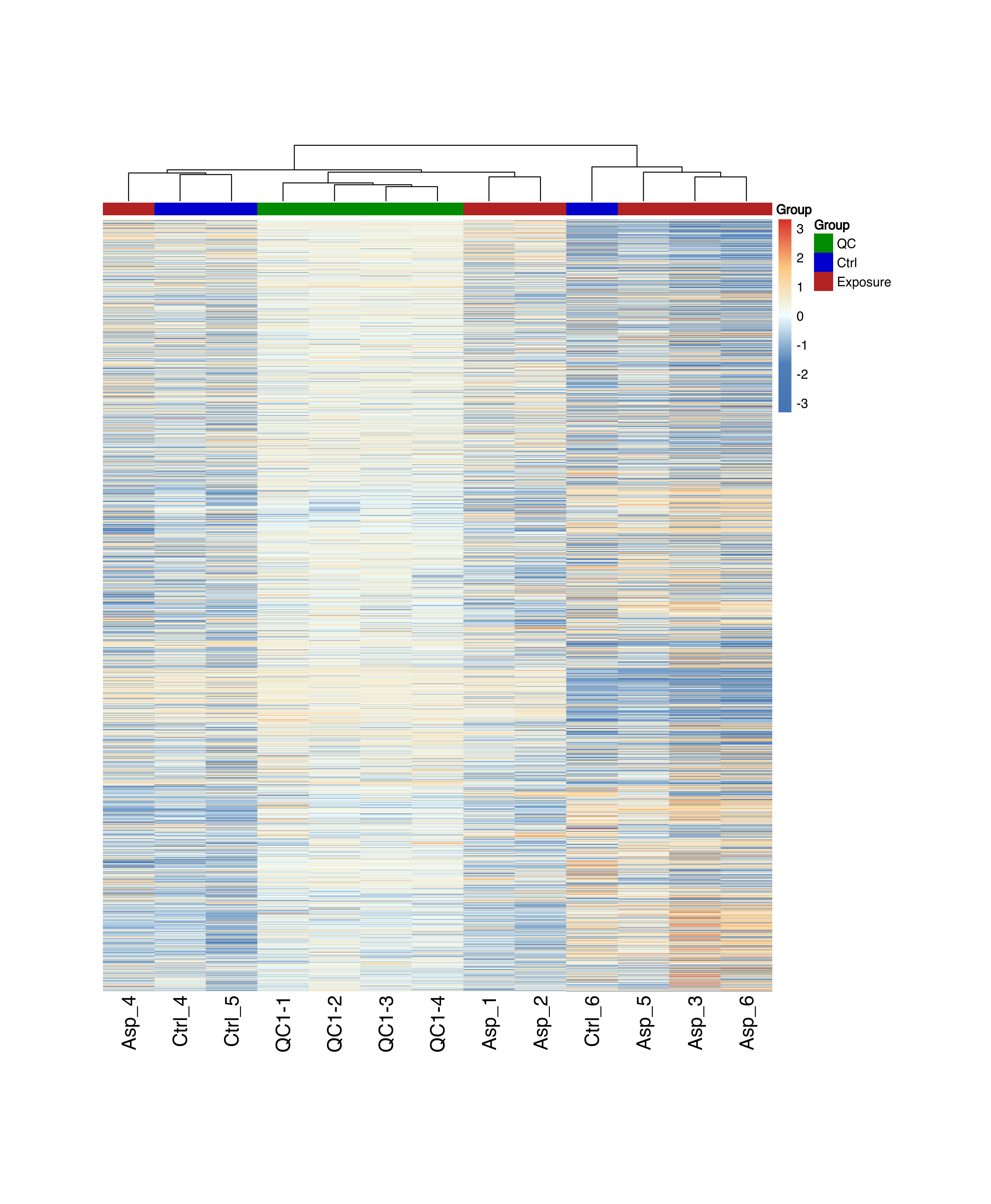

Supplement: Supplementary material S1 — The main instruments used during the LC-MS process, along with their models/specifications and manufacturers. [file Supplementary_file_1.zip › Metabolomics sequencing data FC1.2/2.质量控制/heatmap-qc control.jpg]

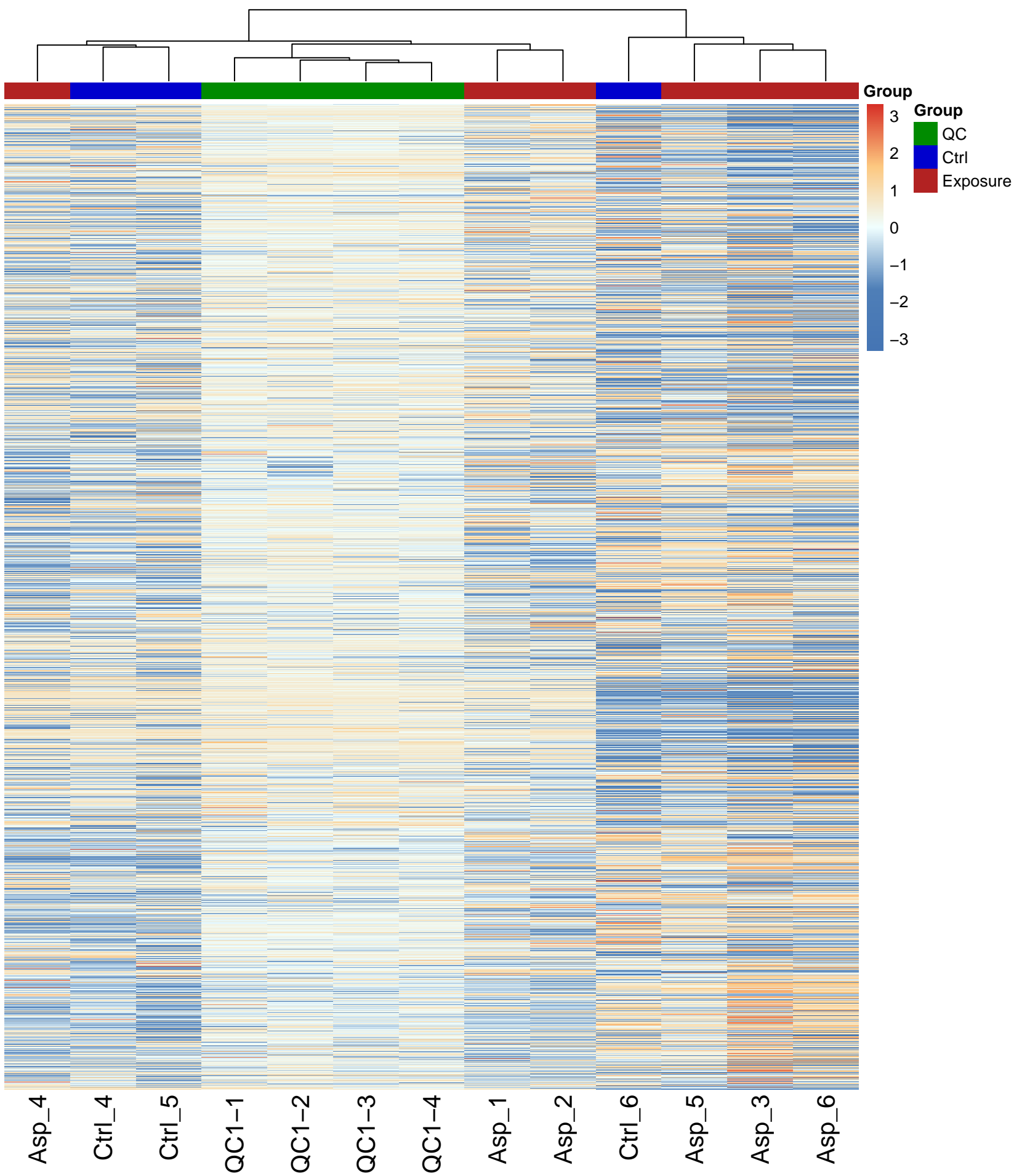

Supplement: Supplementary material S1 — The main instruments used during the LC-MS process, along with their models/specifications and manufacturers. [file Supplementary_file_1.zip › Metabolomics sequencing data FC1.2/2.质量控制/heatmap-qc control.pdf]

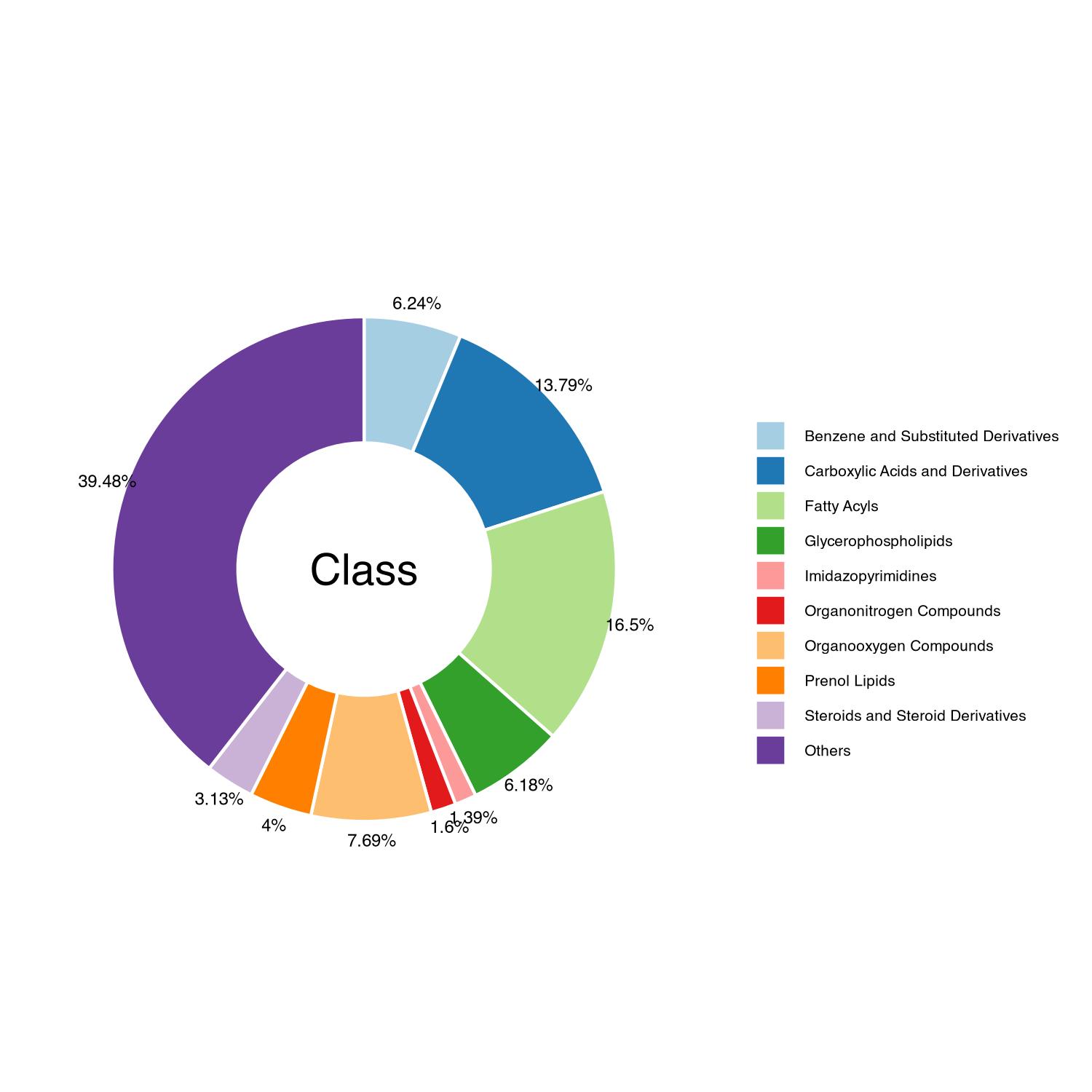

Supplement: Supplementary material S1 — The main instruments used during the LC-MS process, along with their models/specifications and manufacturers. [file Supplementary_file_1.zip › Metabolomics sequencing data FC1.2/3.数据矩阵/Pie_Graph/piechart-Class.jpg]

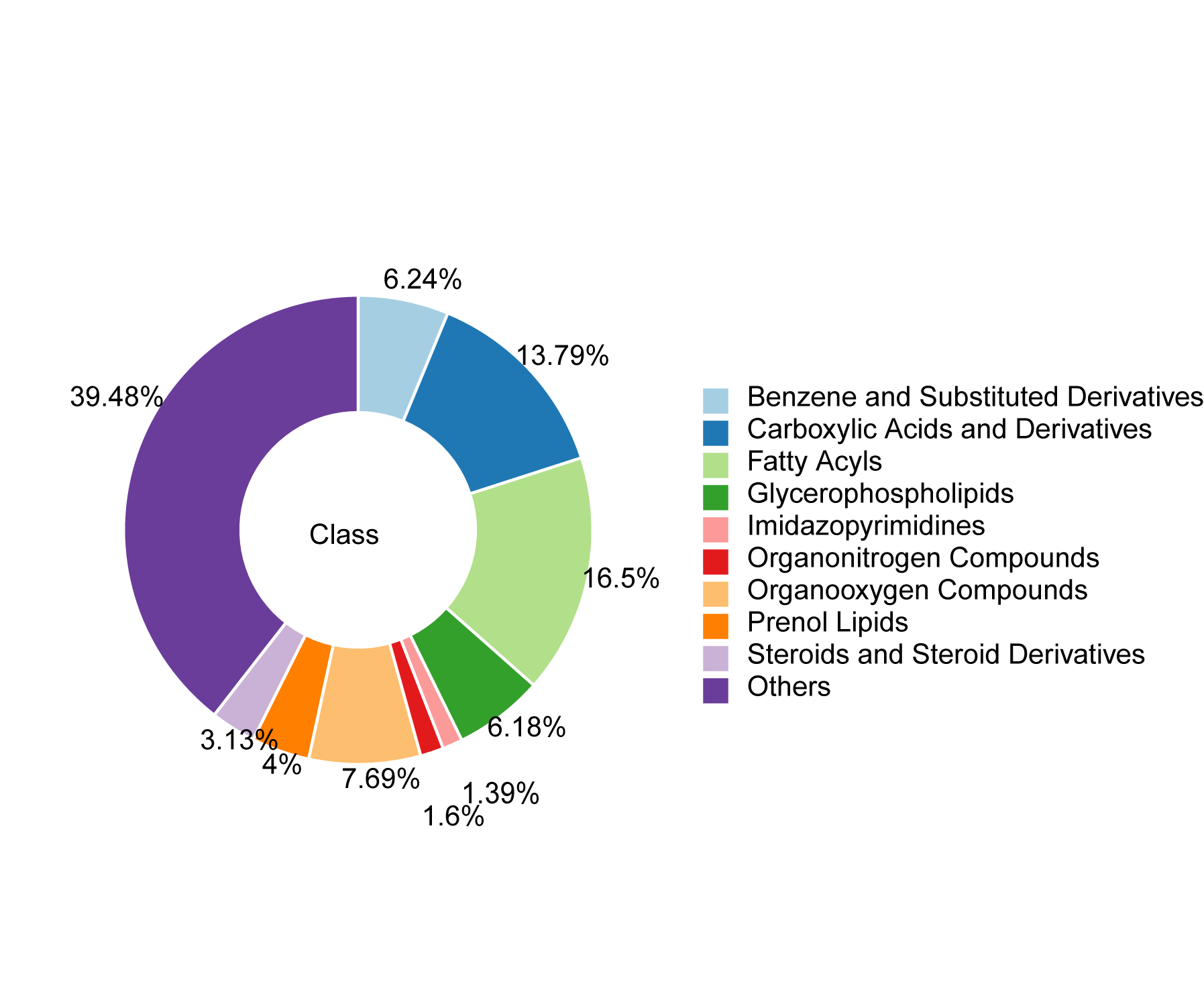

Supplement: Supplementary material S1 — The main instruments used during the LC-MS process, along with their models/specifications and manufacturers. [file Supplementary_file_1.zip › Metabolomics sequencing data FC1.2/3.数据矩阵/Pie_Graph/piechart-Class1.tif]

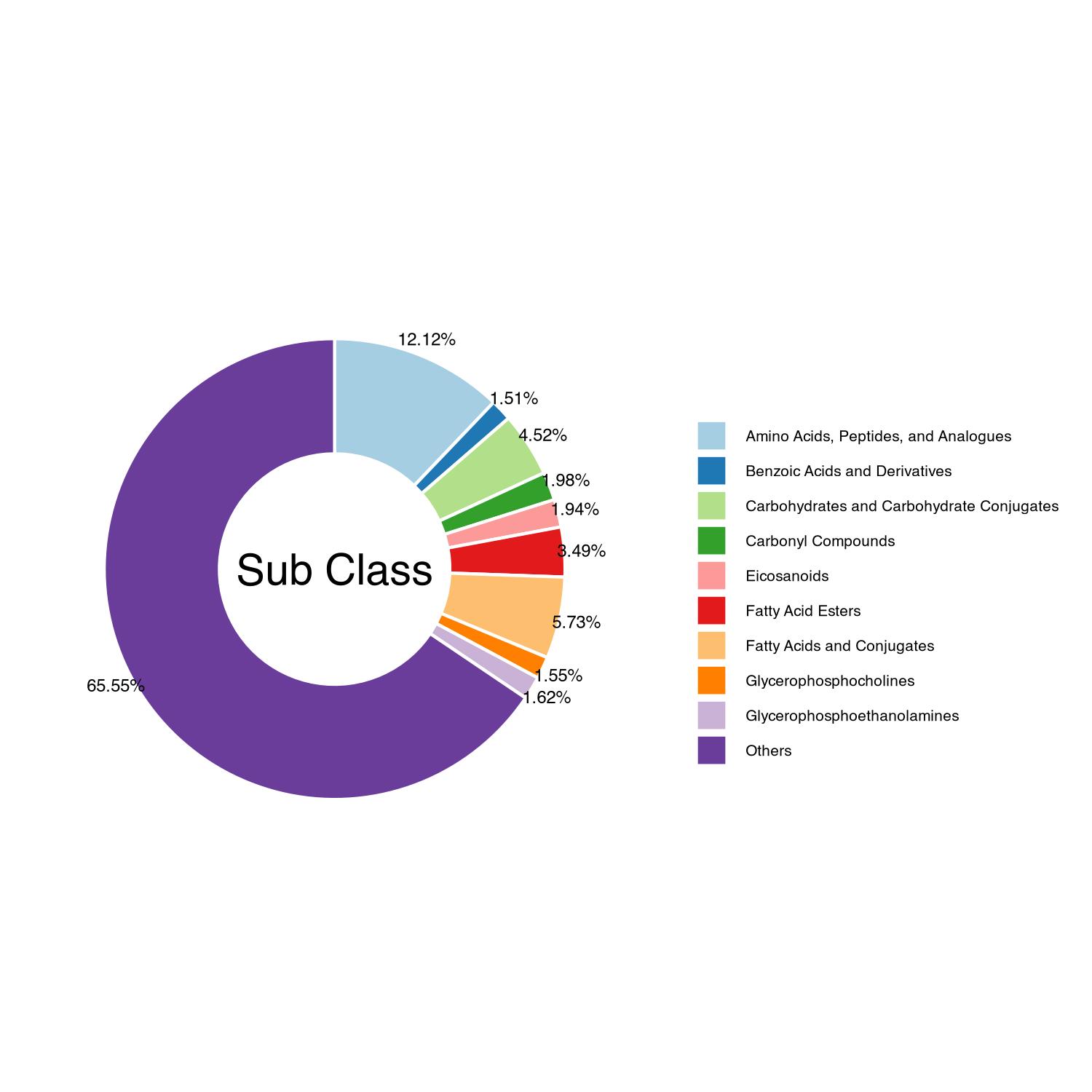

Supplement: Supplementary material S1 — The main instruments used during the LC-MS process, along with their models/specifications and manufacturers. [file Supplementary_file_1.zip › Metabolomics sequencing data FC1.2/3.数据矩阵/Pie_Graph/piechart-Sub Class.jpg]

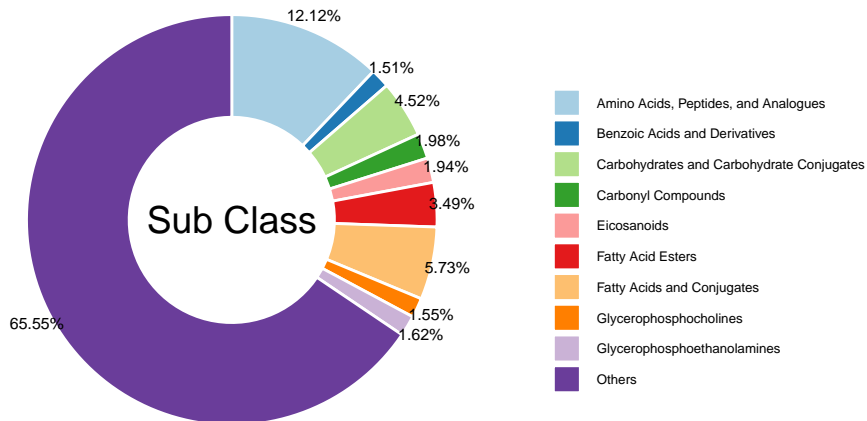

Supplement: Supplementary material S1 — The main instruments used during the LC-MS process, along with their models/specifications and manufacturers. [file Supplementary_file_1.zip › Metabolomics sequencing data FC1.2/3.数据矩阵/Pie_Graph/piechart-Sub Class.pdf]

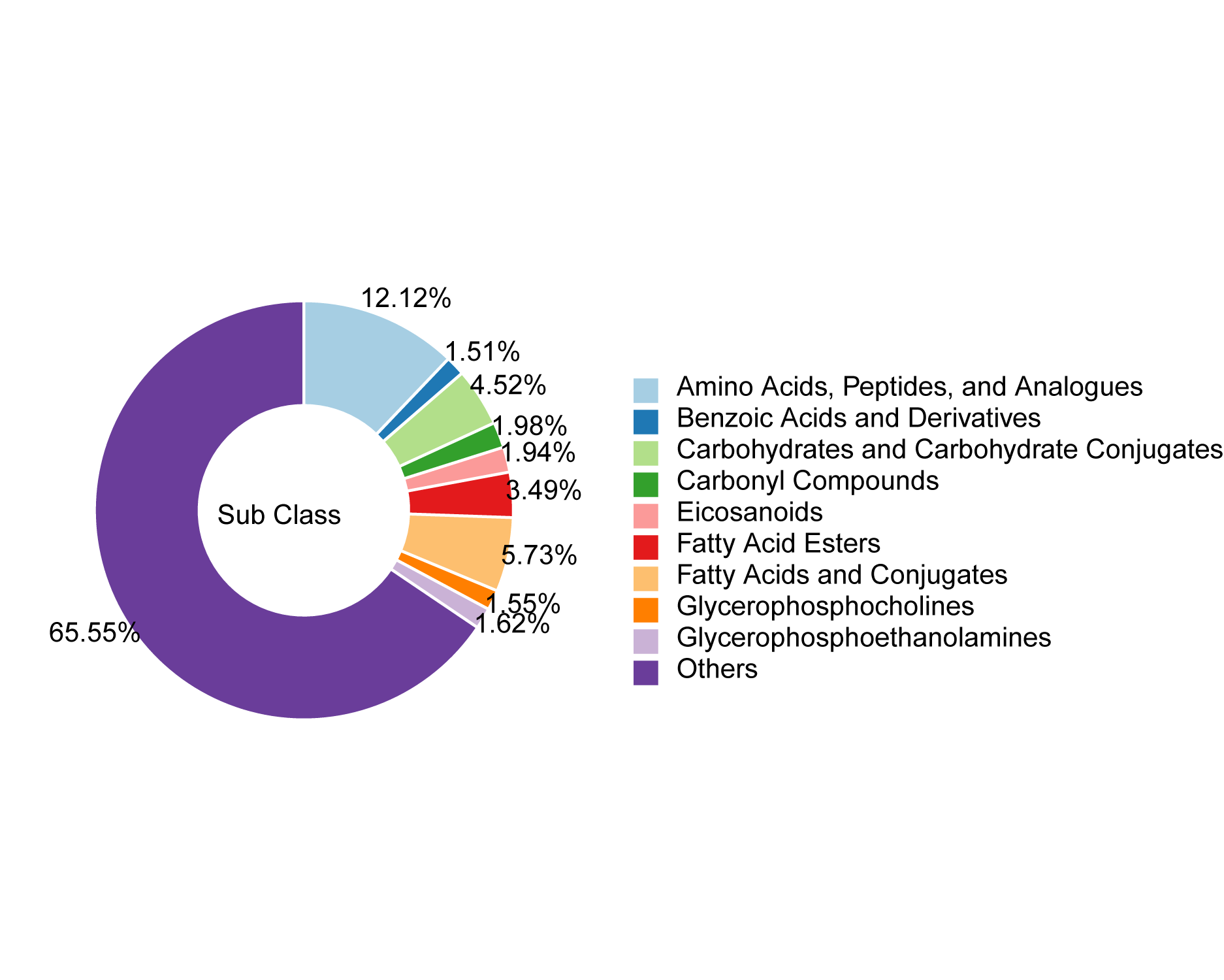

Supplement: Supplementary material S1 — The main instruments used during the LC-MS process, along with their models/specifications and manufacturers. [file Supplementary_file_1.zip › Metabolomics sequencing data FC1.2/3.数据矩阵/Pie_Graph/piechart-Sub Class.tif]

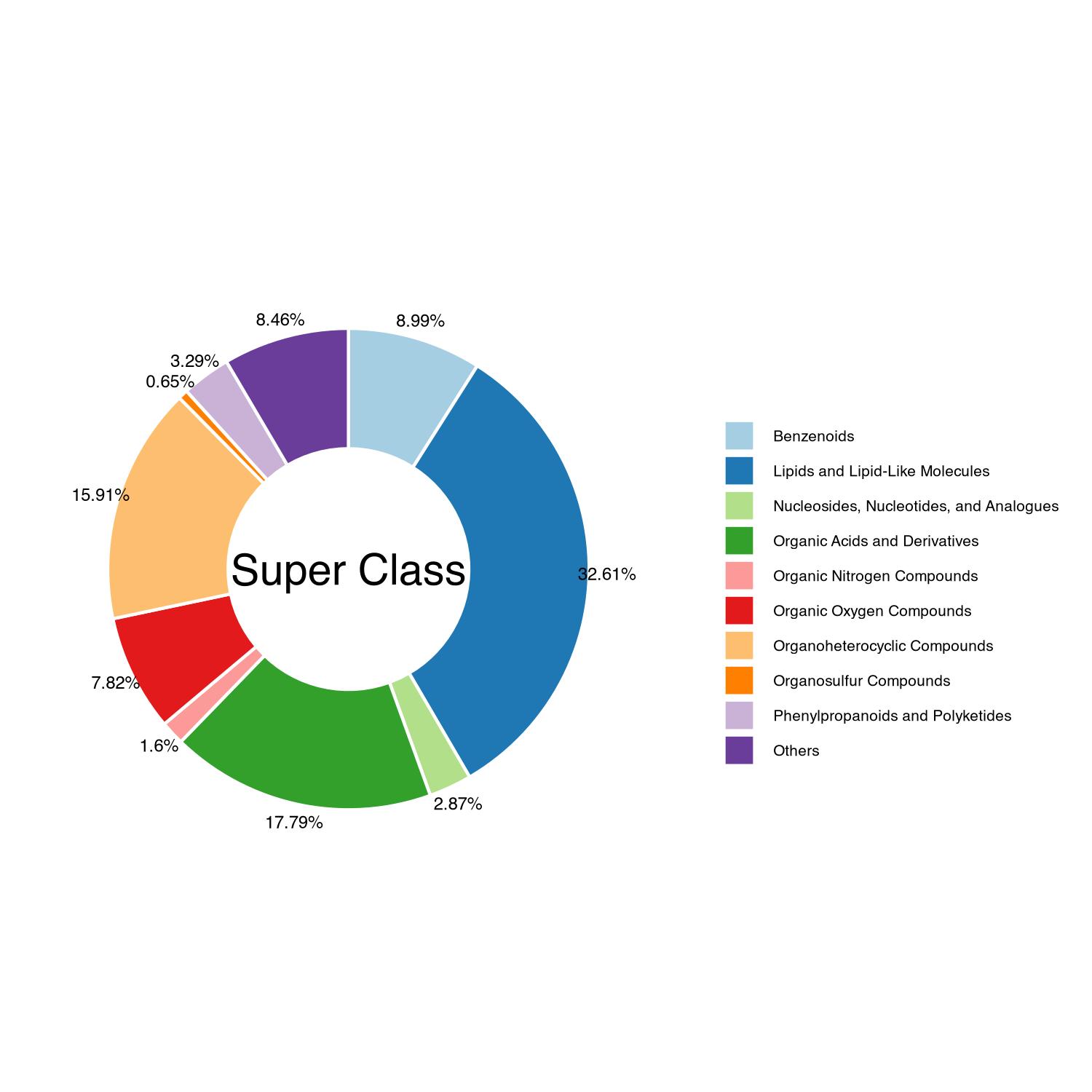

Supplement: Supplementary material S1 — The main instruments used during the LC-MS process, along with their models/specifications and manufacturers. [file Supplementary_file_1.zip › Metabolomics sequencing data FC1.2/3.数据矩阵/Pie_Graph/piechart-Super Class.jpg]

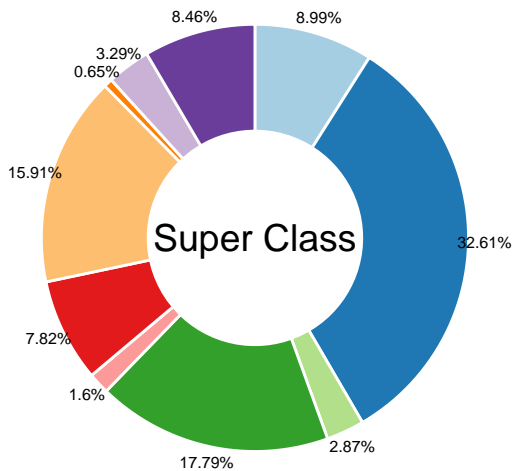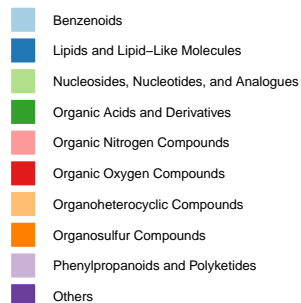

Supplement: Supplementary material S1 — The main instruments used during the LC-MS process, along with their models/specifications and manufacturers. [file Supplementary_file_1.zip › Metabolomics sequencing data FC1.2/3.数据矩阵/Pie_Graph/piechart-Super Class.pdf]

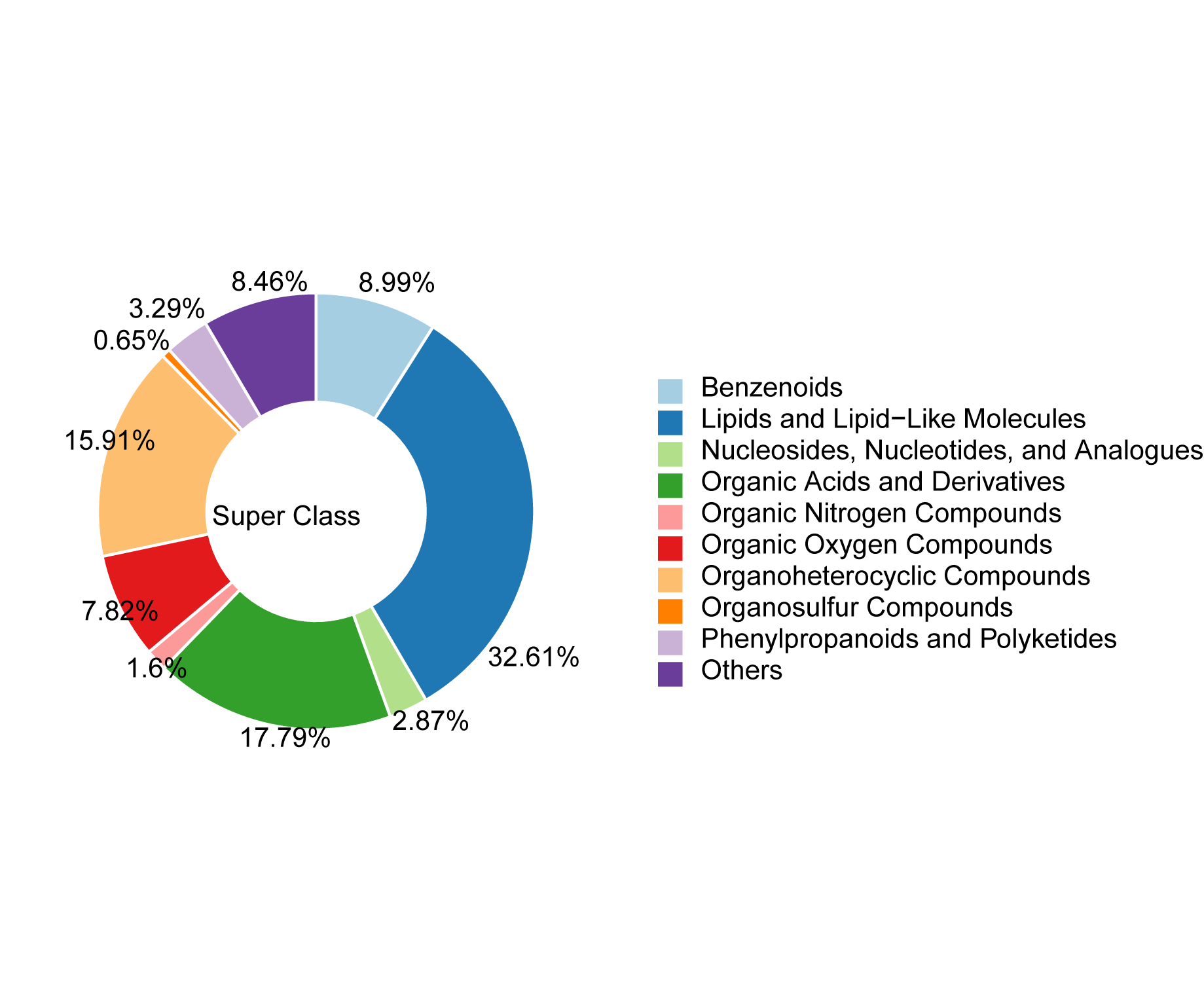

Supplement: Supplementary material S1 — The main instruments used during the LC-MS process, along with their models/specifications and manufacturers. [file Supplementary_file_1.zip › Metabolomics sequencing data FC1.2/3.数据矩阵/Pie_Graph/piechart-Super Class.tif]

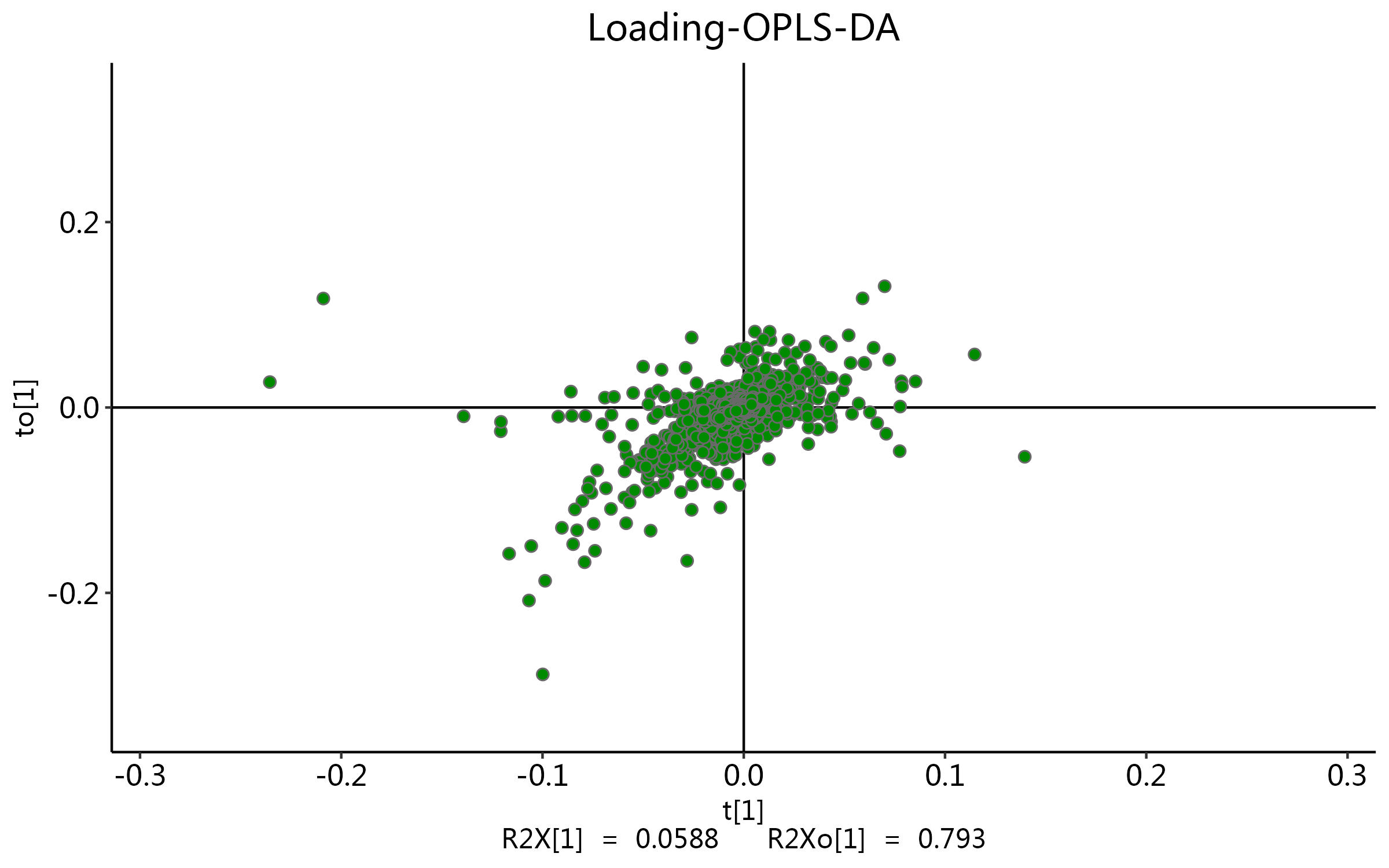

Supplement: Supplementary material S1 — The main instruments used during the LC-MS process, along with their models/specifications and manufacturers. [file Supplementary_file_1.zip › Metabolomics sequencing data FC1.2/4.多元统计分析/Loading_plot/Loading-OPLS-DA(Exposure_Ctrl).jpg]

# Loading-OPLS-DA

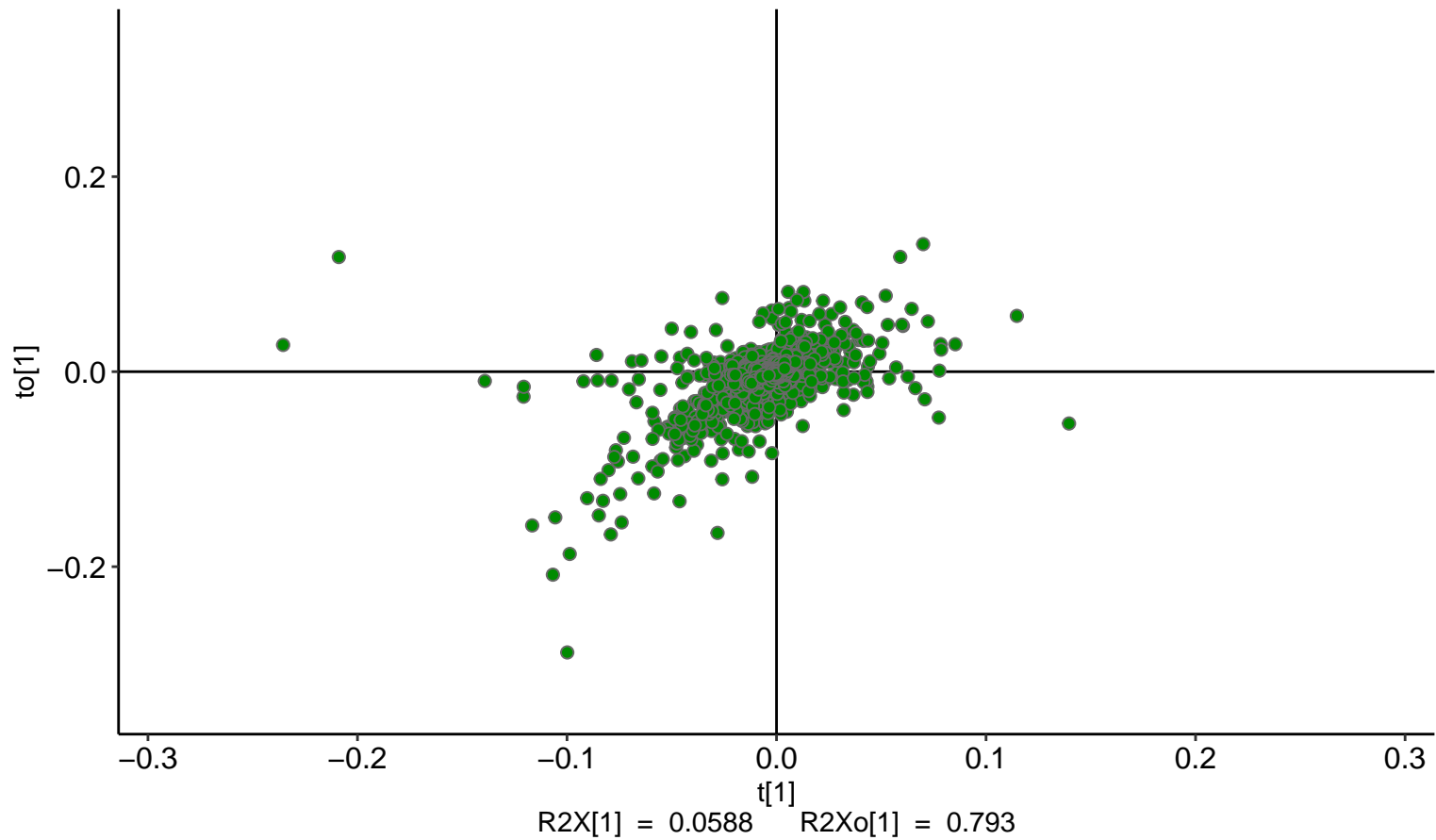

Supplement: Supplementary material S1 — The main instruments used during the LC-MS process, along with their models/specifications and manufacturers. [file Supplementary_file_1.zip › Metabolomics sequencing data FC1.2/4.多元统计分析/Loading_plot/Loading-OPLS-DA(Exposure_Ctrl).pdf]

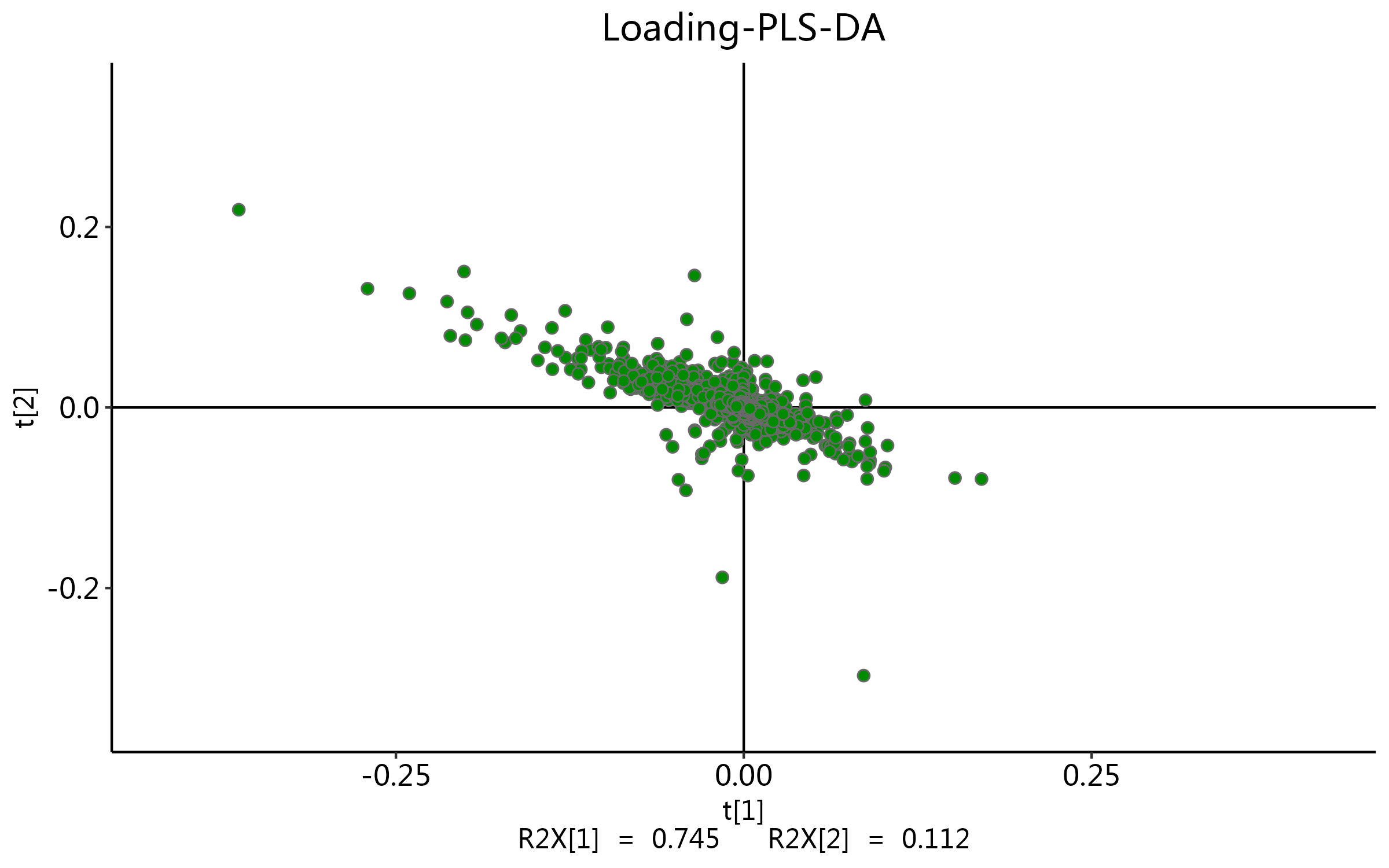

Supplement: Supplementary material S1 — The main instruments used during the LC-MS process, along with their models/specifications and manufacturers. [file Supplementary_file_1.zip › Metabolomics sequencing data FC1.2/4.多元统计分析/Loading_plot/Loading-PLS-DA(Allsample).jpg]

Loading-PLS-DA

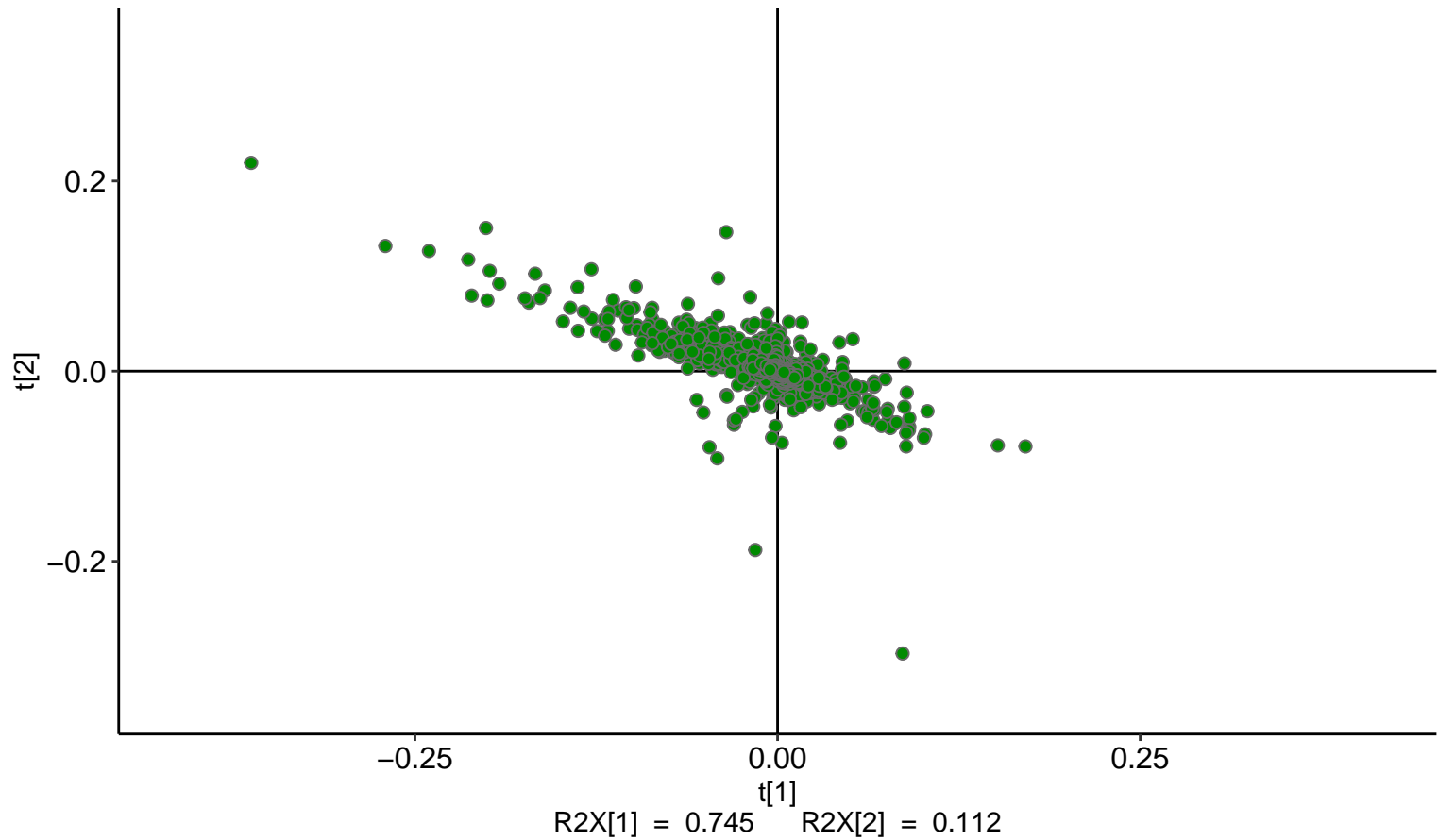

Supplement: Supplementary material S1 — The main instruments used during the LC-MS process, along with their models/specifications and manufacturers. [file Supplementary_file_1.zip › Metabolomics sequencing data FC1.2/4.多元统计分析/Loading_plot/Loading-PLS-DA(Allsample).pdf]

Loading-PLS-DA

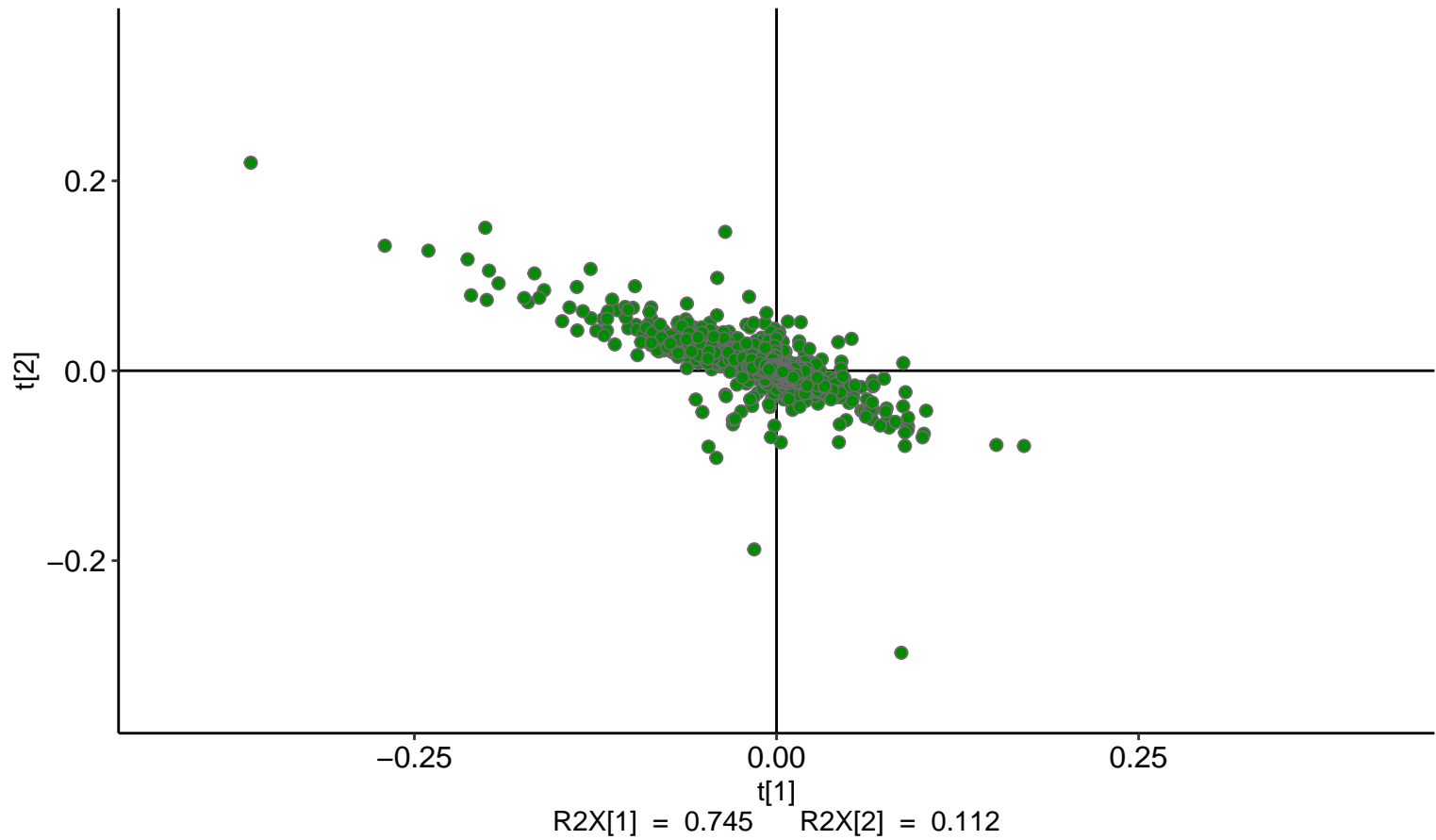

Supplement: Supplementary material S1 — The main instruments used during the LC-MS process, along with their models/specifications and manufacturers. [file Supplementary_file_1.zip › Metabolomics sequencing data FC1.2/4.多元统计分析/Loading_plot/Loading-PLS-DA(Exposure_Ctrl).pdf]

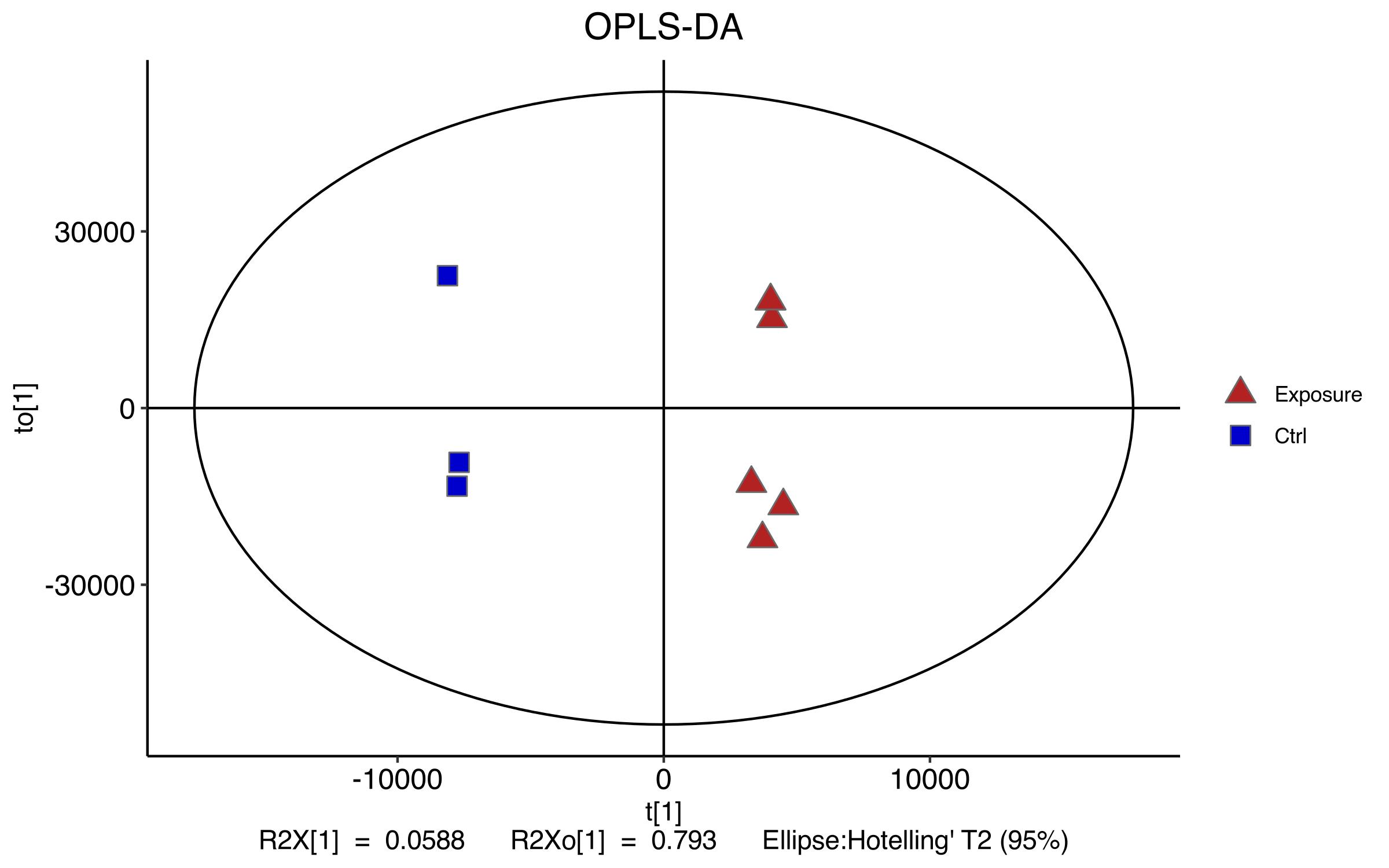

Supplement: Supplementary material S1 — The main instruments used during the LC-MS process, along with their models/specifications and manufacturers. [file Supplementary_file_1.zip › Metabolomics sequencing data FC1.2/4.多元统计分析/OPLS/OPLS-DA(Exposure_Ctrl).jpg]

# OPLS-DA

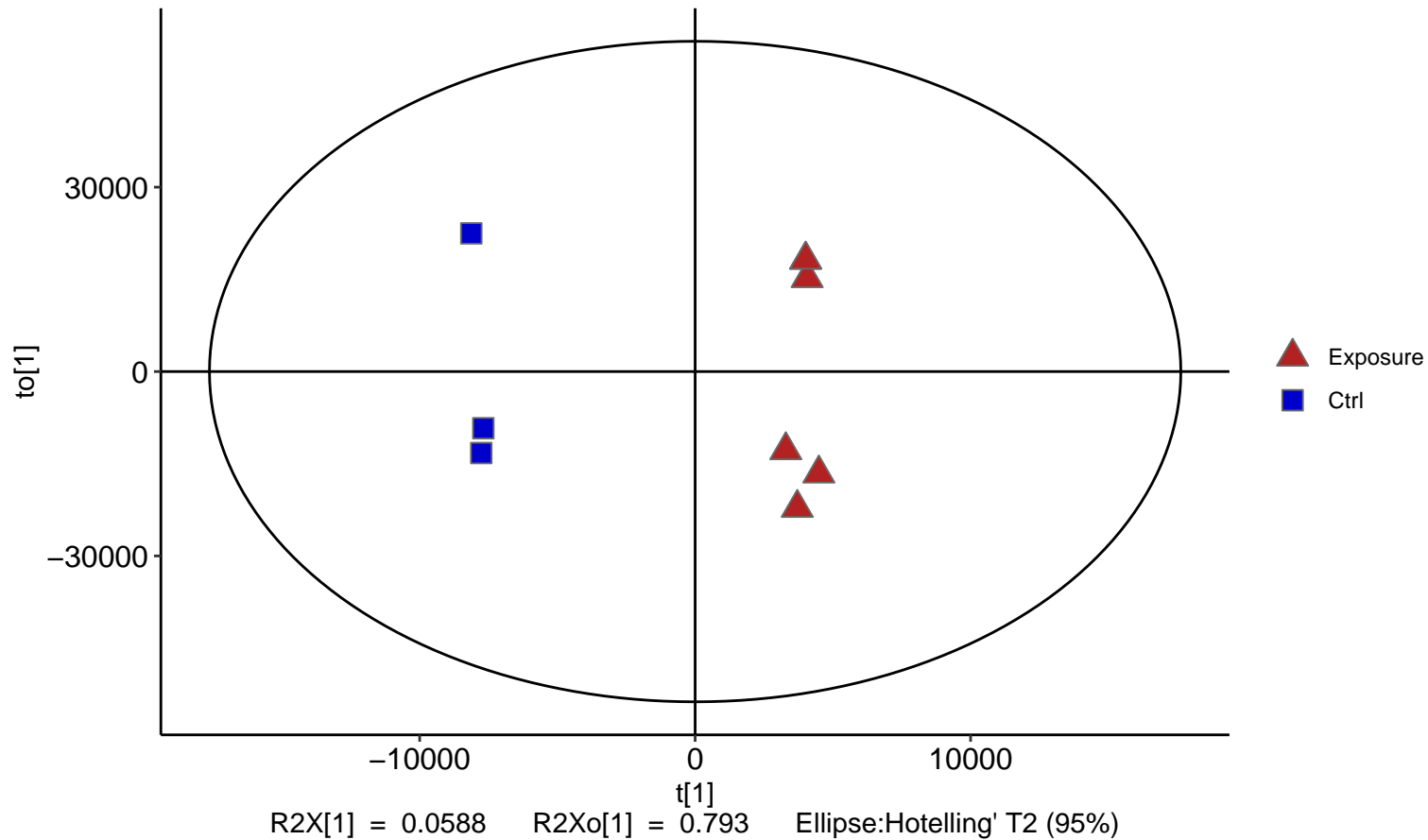

Supplement: Supplementary material S1 — The main instruments used during the LC-MS process, along with their models/specifications and manufacturers. [file Supplementary_file_1.zip › Metabolomics sequencing data FC1.2/4.多元统计分析/OPLS/OPLS-DA(Exposure_Ctrl).pdf]

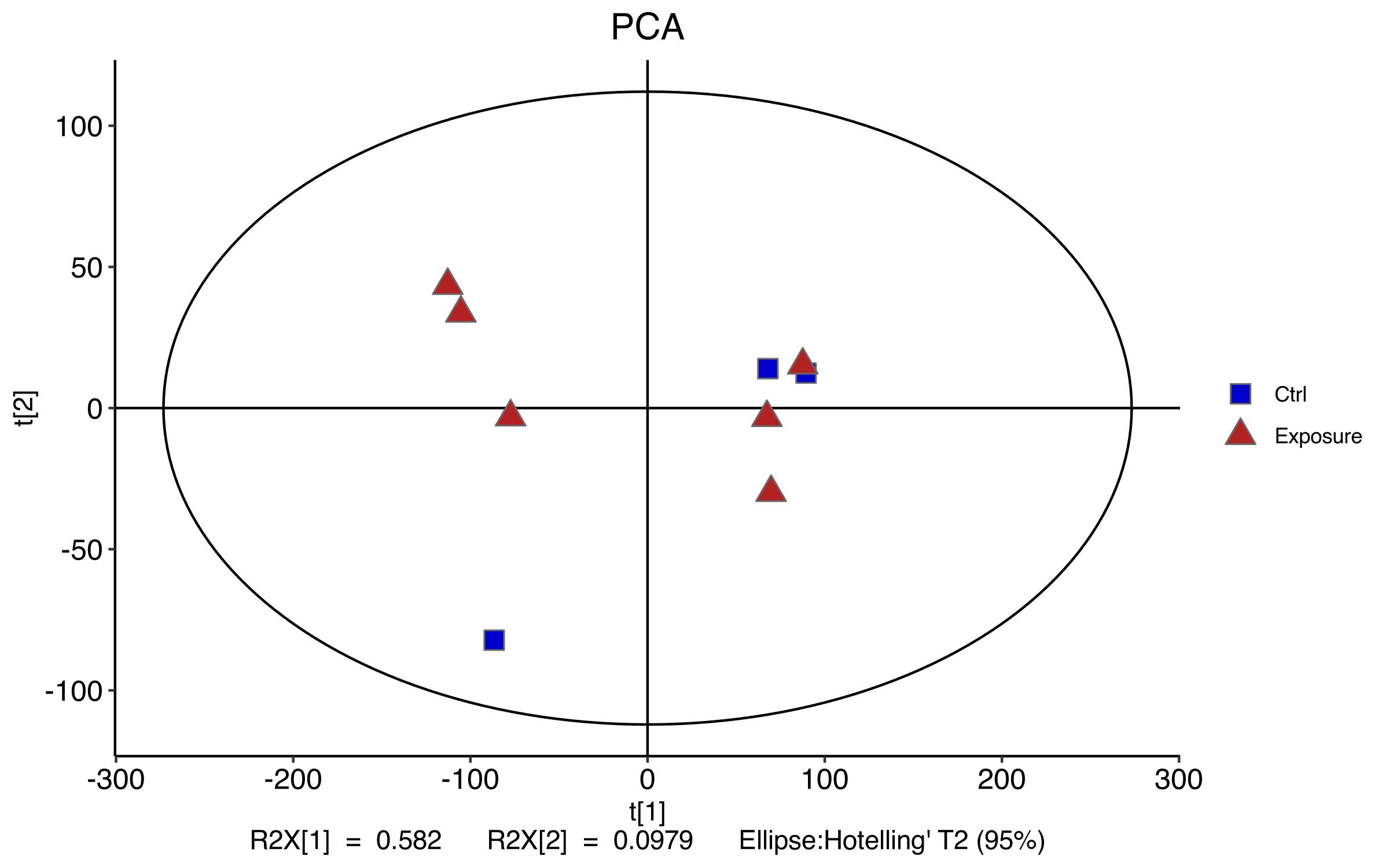

Supplement: Supplementary material S1 — The main instruments used during the LC-MS process, along with their models/specifications and manufacturers. [file Supplementary_file_1.zip › Metabolomics sequencing data FC1.2/4.多元统计分析/PCA/PCA(Allsample).jpg]

## PCA

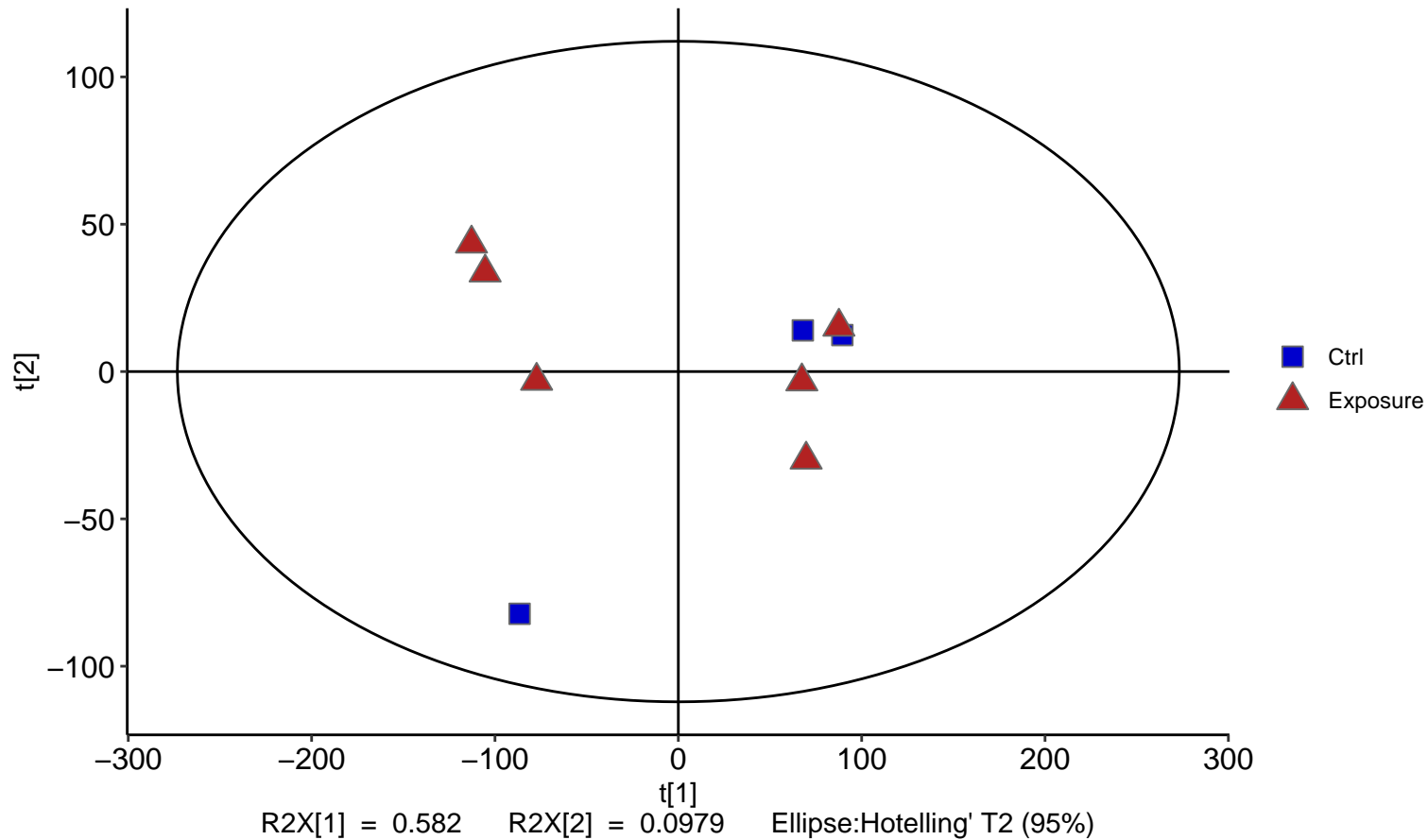

Supplement: Supplementary material S1 — The main instruments used during the LC-MS process, along with their models/specifications and manufacturers. [file Supplementary_file_1.zip › Metabolomics sequencing data FC1.2/4.多元统计分析/PCA/PCA(Allsample).pdf]

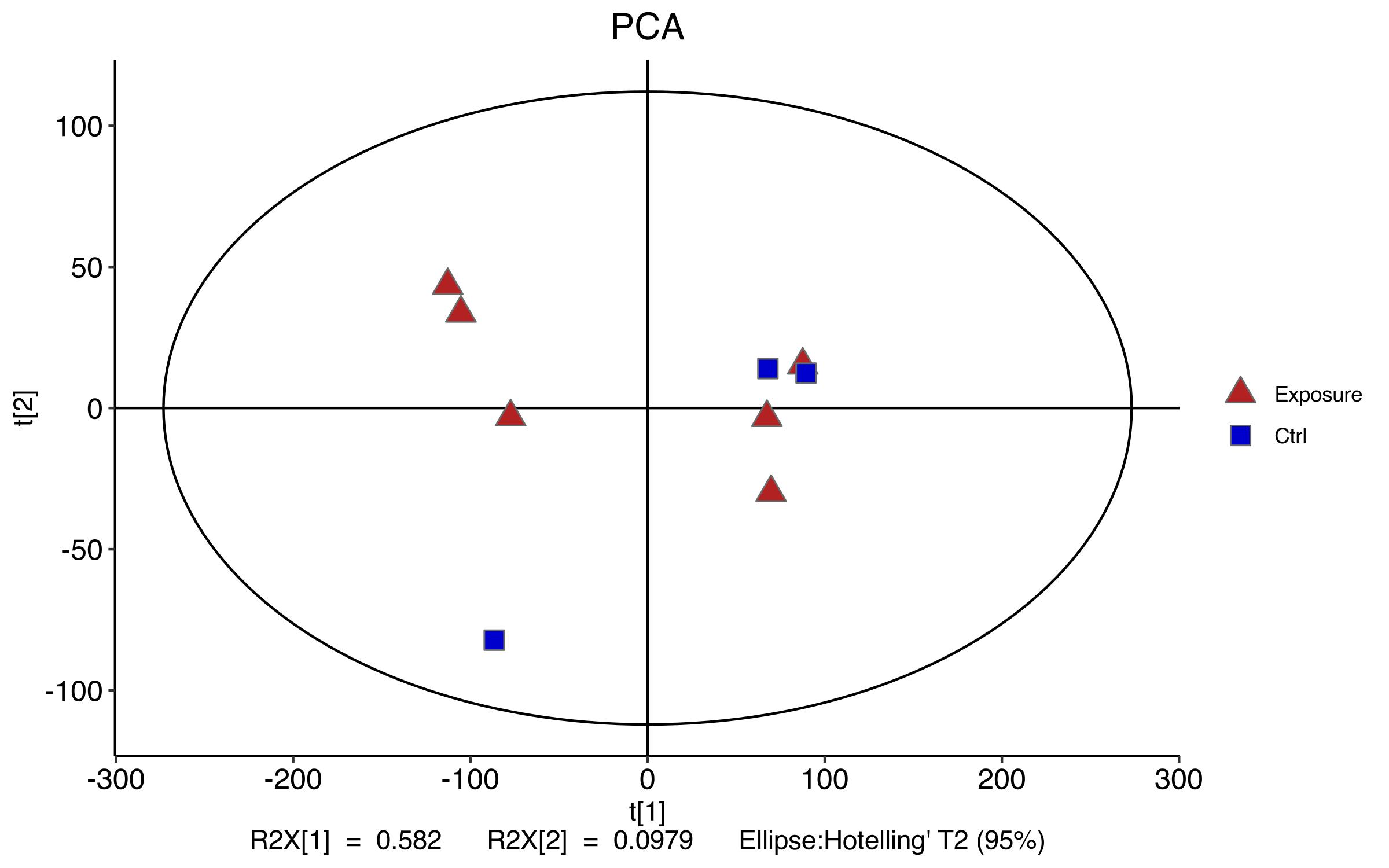

Supplement: Supplementary material S1 — The main instruments used during the LC-MS process, along with their models/specifications and manufacturers. [file Supplementary_file_1.zip › Metabolomics sequencing data FC1.2/4.多元统计分析/PCA/PCA(Exposure_Ctrl).jpg]

## PCA

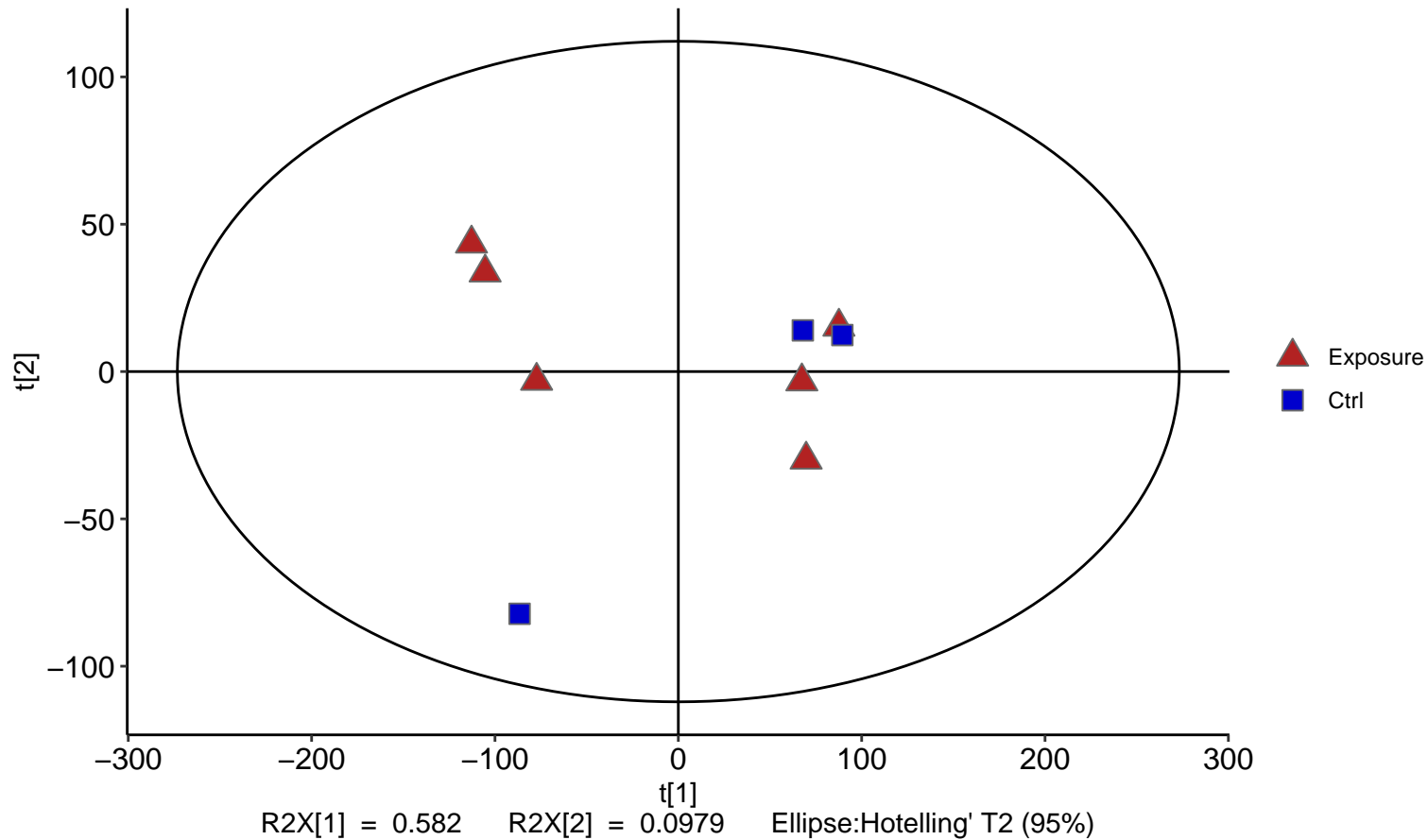

Supplement: Supplementary material S1 — The main instruments used during the LC-MS process, along with their models/specifications and manufacturers. [file Supplementary_file_1.zip › Metabolomics sequencing data FC1.2/4.多元统计分析/PCA/PCA(Exposure_Ctrl).pdf]

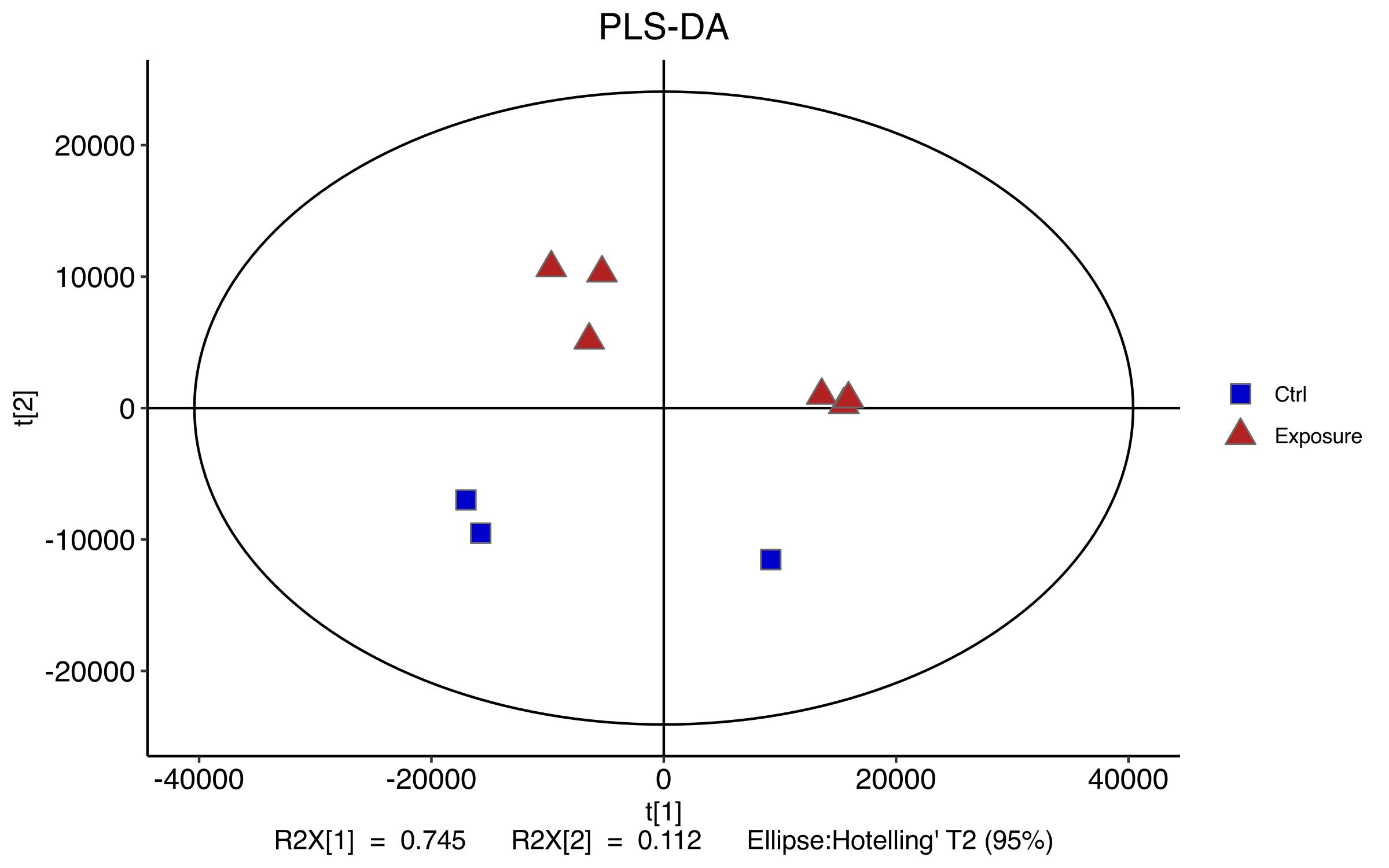

Supplement: Supplementary material S1 — The main instruments used during the LC-MS process, along with their models/specifications and manufacturers. [file Supplementary_file_1.zip › Metabolomics sequencing data FC1.2/4.多元统计分析/PLS/PLS-DA(Allsample).jpg]

# PLS-DA

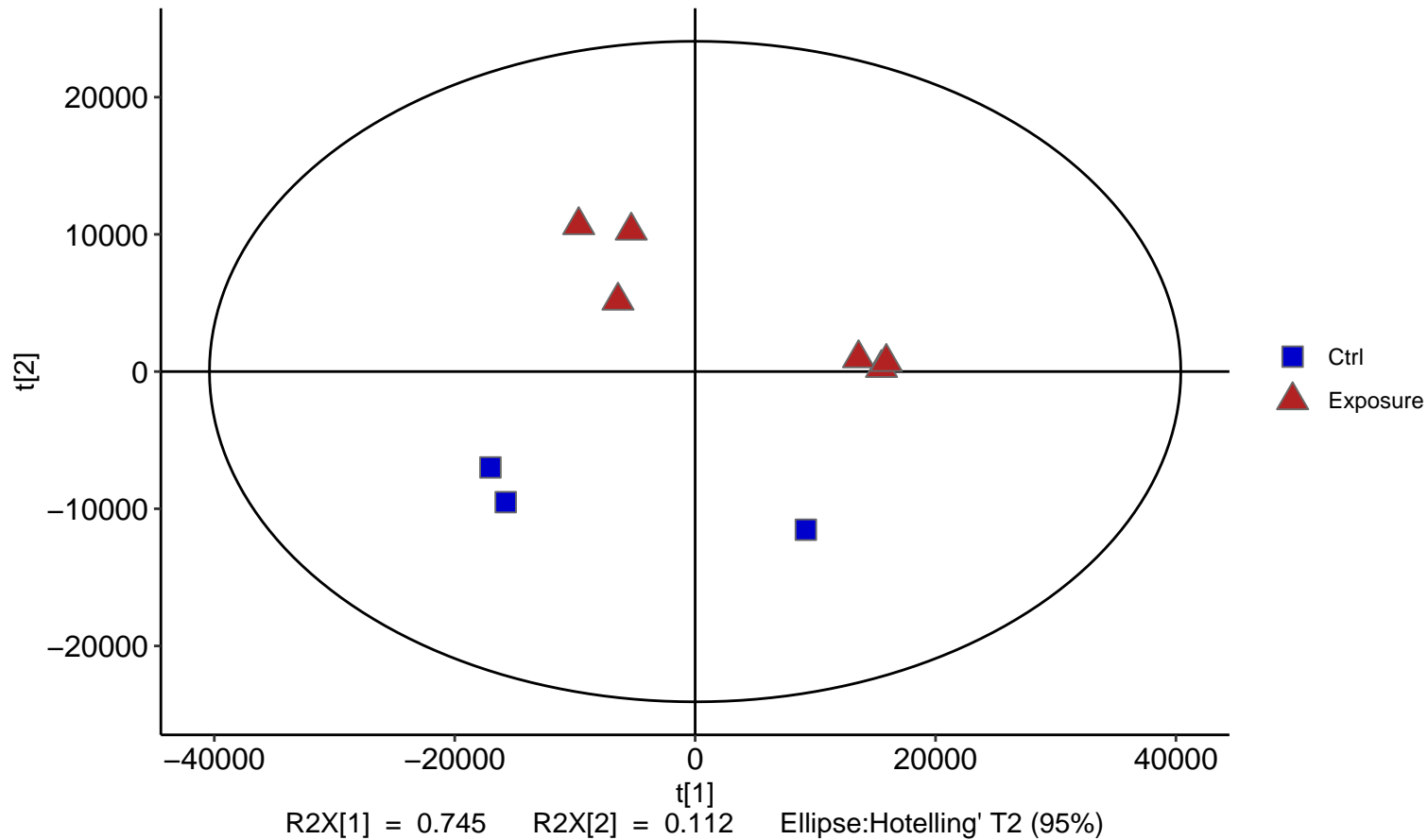

Supplement: Supplementary material S1 — The main instruments used during the LC-MS process, along with their models/specifications and manufacturers. [file Supplementary_file_1.zip › Metabolomics sequencing data FC1.2/4.多元统计分析/PLS/PLS-DA(Allsample).pdf]

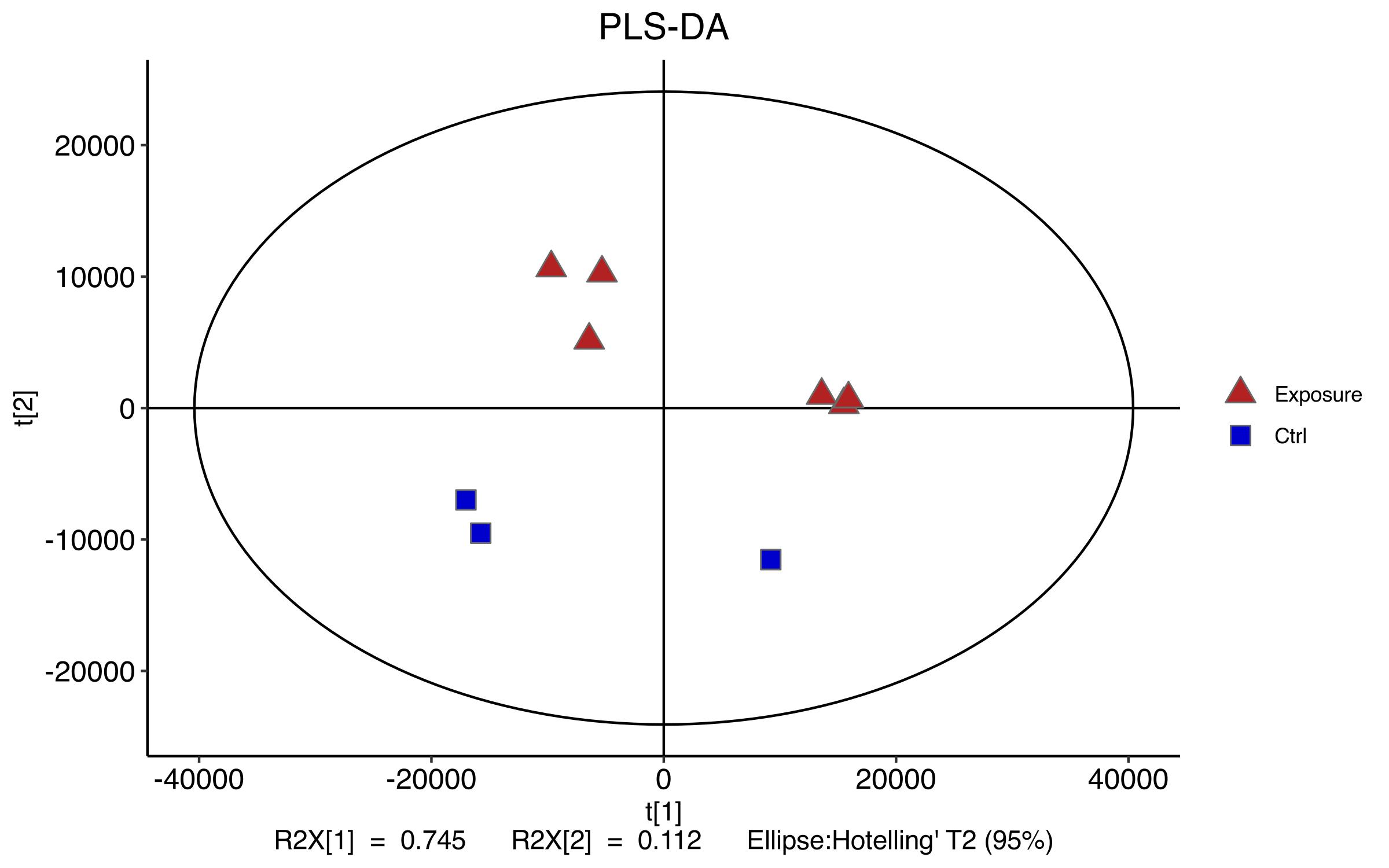

Supplement: Supplementary material S1 — The main instruments used during the LC-MS process, along with their models/specifications and manufacturers. [file Supplementary_file_1.zip › Metabolomics sequencing data FC1.2/4.多元统计分析/PLS/PLS-DA(Exposure_Ctrl).jpg]

## PLS-DA

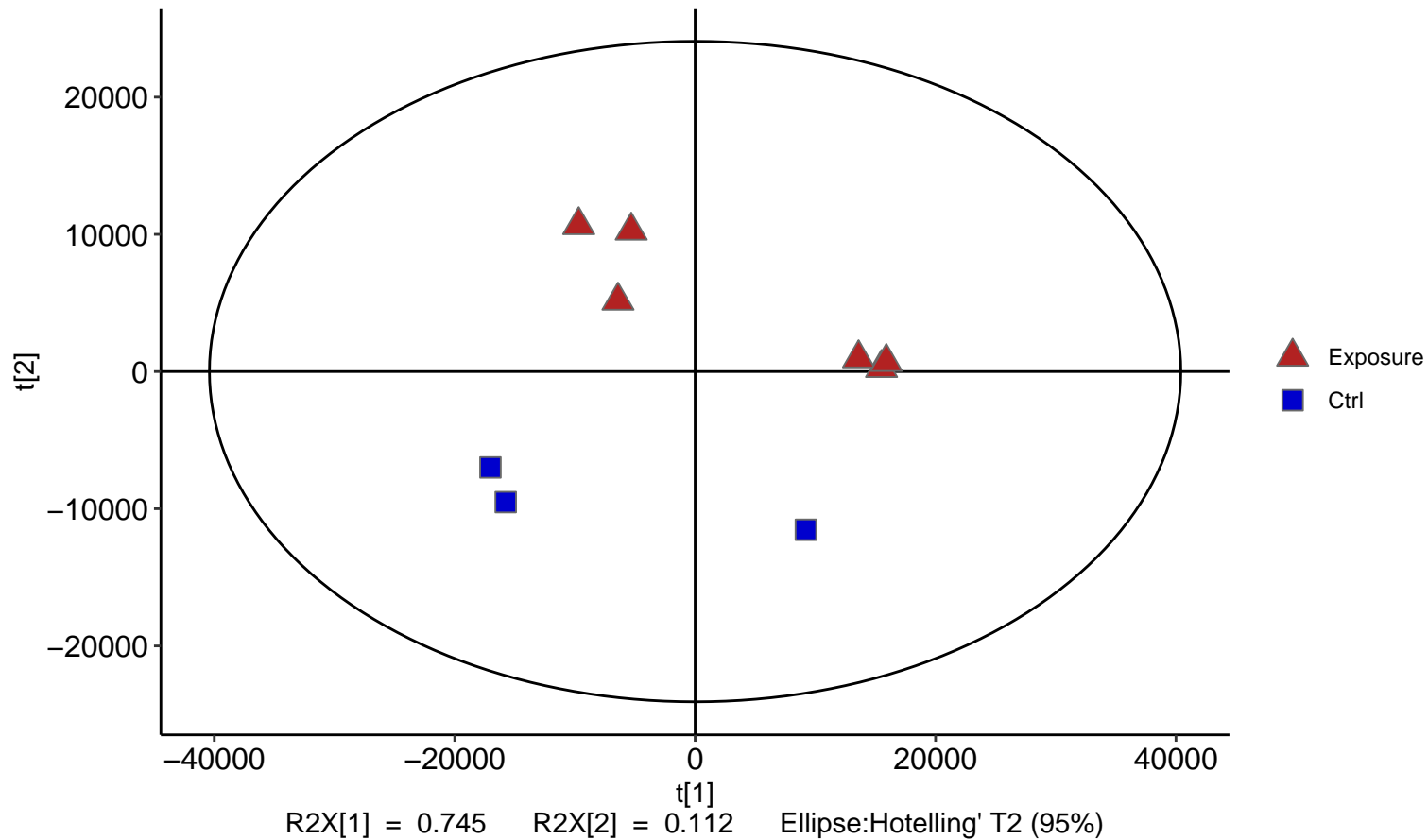

Supplement: Supplementary material S1 — The main instruments used during the LC-MS process, along with their models/specifications and manufacturers. [file Supplementary_file_1.zip › Metabolomics sequencing data FC1.2/4.多元统计分析/PLS/PLS-DA(Exposure_Ctrl).pdf]

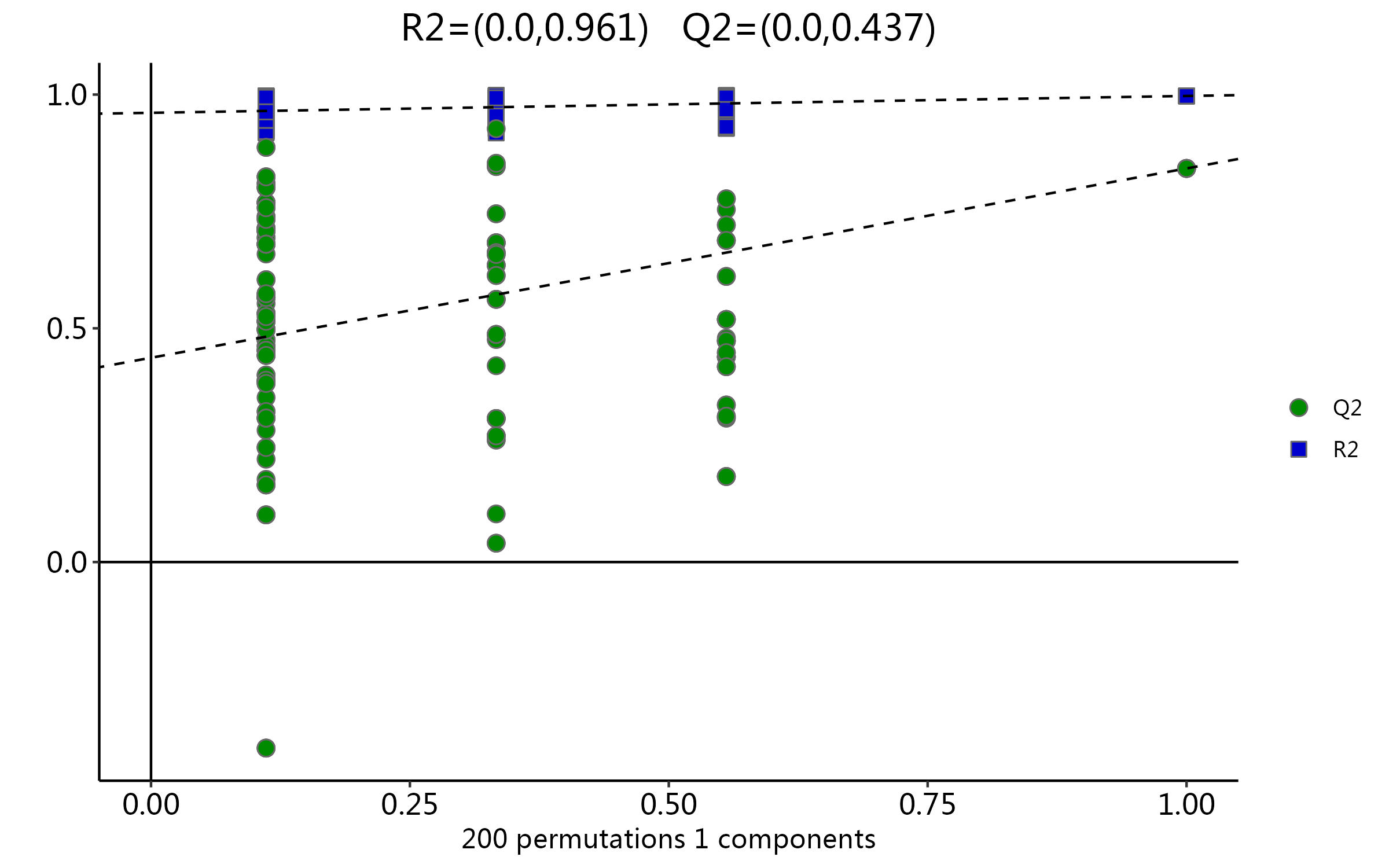

Supplement: Supplementary material S1 — The main instruments used during the LC-MS process, along with their models/specifications and manufacturers. [file Supplementary_file_1.zip › Metabolomics sequencing data FC1.2/4.多元统计分析/Permutation/Permutation(Allsample).jpg]

$R^2=(0.0,0.961)$   $Q^2=(0.0,0.437)$

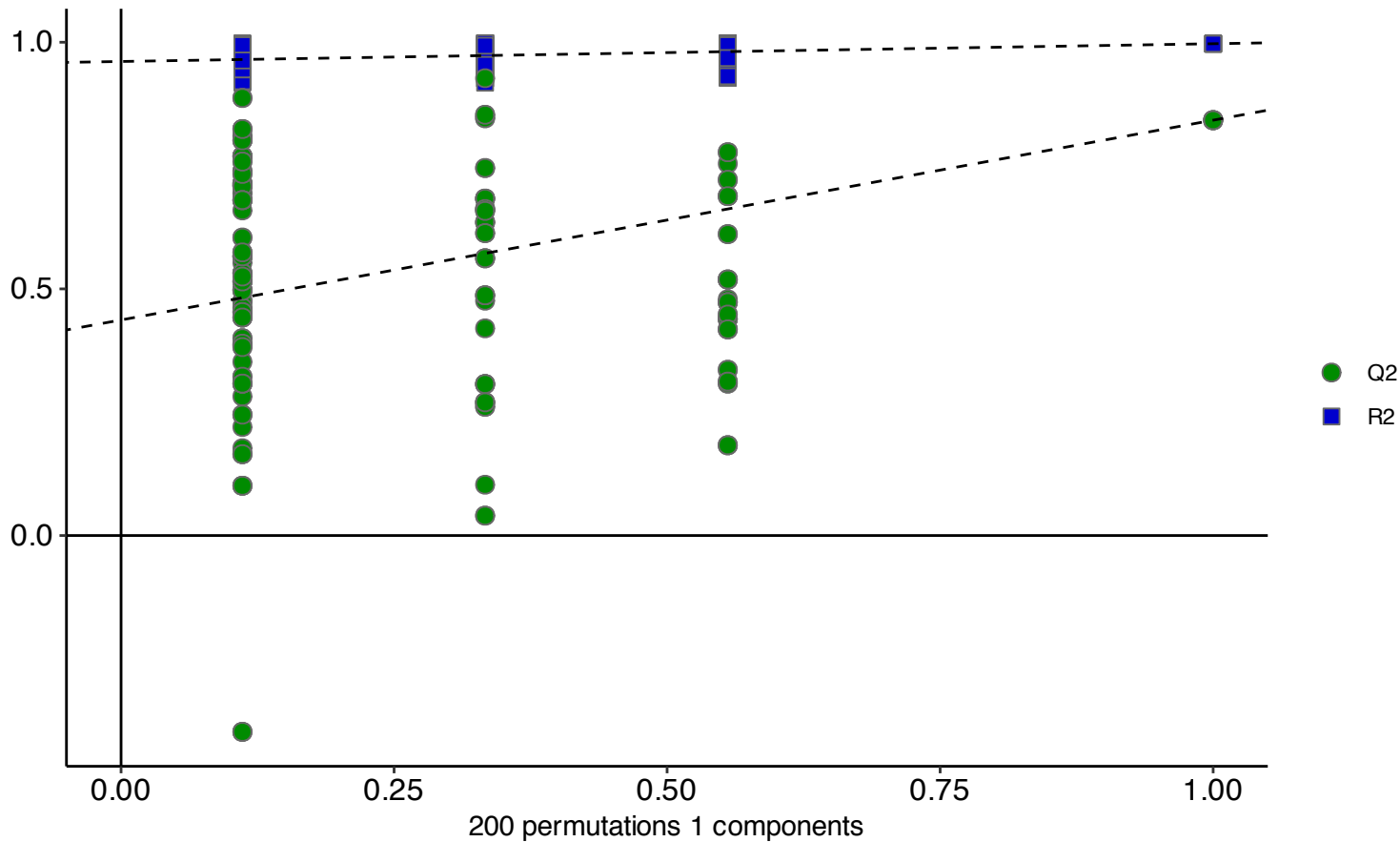

Supplement: Supplementary material S1 — The main instruments used during the LC-MS process, along with their models/specifications and manufacturers. [file Supplementary_file_1.zip › Metabolomics sequencing data FC1.2/4.多元统计分析/Permutation/Permutation(Allsample).pdf]

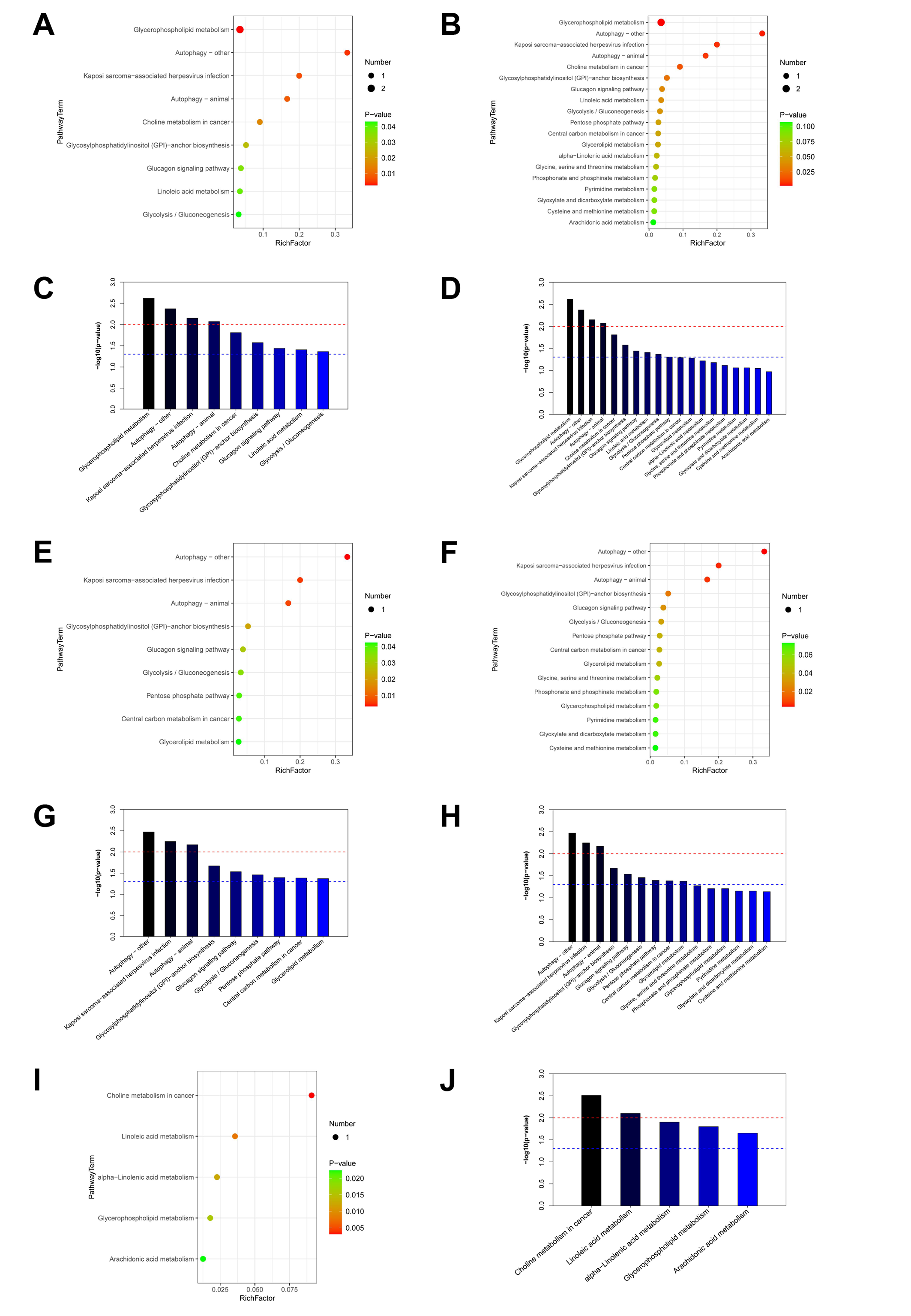

Supplement: Supplementary material S9 — Data matrix of classification pie charts. [file Image_1.TIF]

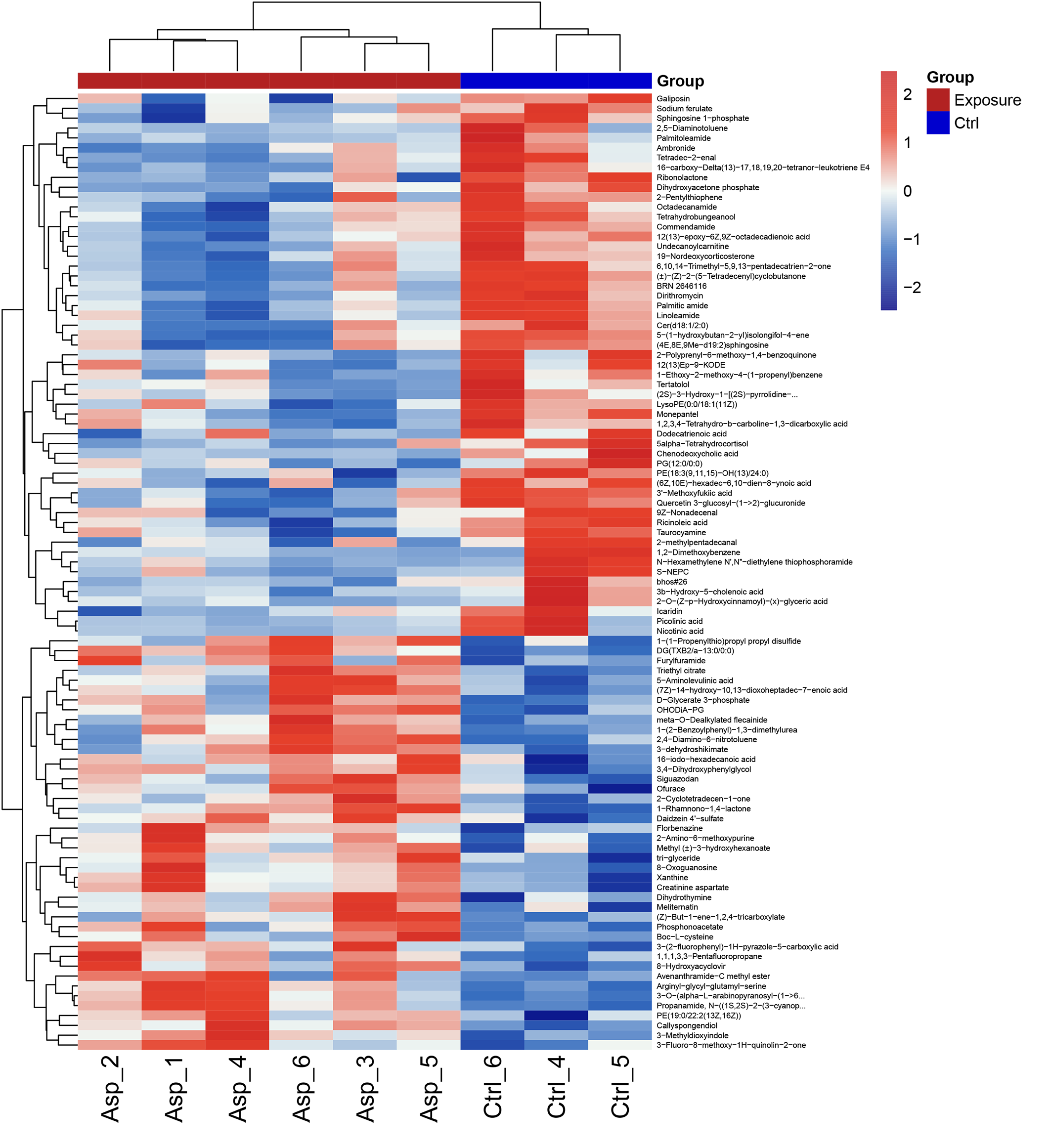

Supplement: Supplementary material S10 — Summary table of metabolite pathways. [file Image_2.TIF]

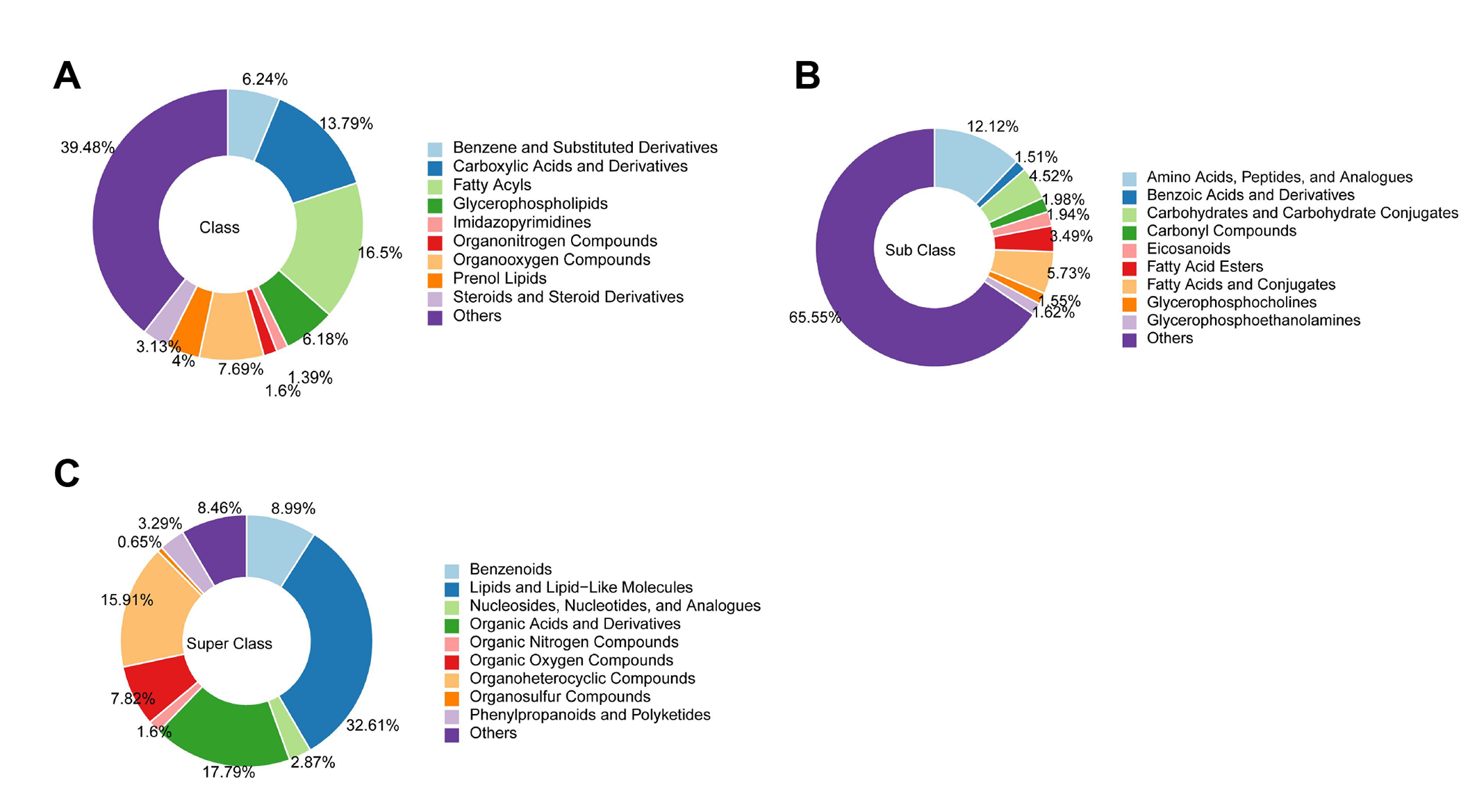

Supplement: Supplementary file 11 [file Image_3.TIF]
