# Supplementary material for: Angelica sinensis polysaccharide as potential protectants against recurrent spontaneous abortion: focus on autophagy regulation
Source: Front Med (Lausanne). 2025 Jan 15;12:1522503. doi: 10.3389/fmed.2025.1522503 (PMC11774876; doi:10.3389/fmed.2025.1522503)

**Supplementary material 2:** Calculation method for *P*-value in KEGG analysis. N represents the total number of metabolites; n represents the number of differentially expressed metabolites within N; M represents the number of metabolites annotated to a specific pathway; m represents the number of differentially expressed metabolites annotated to that specific pathway. A threshold of *P*-value ≤ 0.05 is used, and pathways meeting this criterion indicate significant enrichment of differential metabolites. The smaller the *P*-value, the more significant the differences in the metabolic pathway.


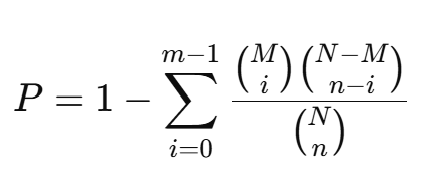

Supplement: Supplementary material S3 — Detailed clinical information of RSA group and NC group. [file Table_2.DOC]
